# Filling the gap - COI barcode resolution in eastern Palearctic birds

**DOI:** 10.1186/1742-9994-6-29

**Published:** 2009-12-09

**Authors:** Kevin CR Kerr, Sharon M Birks, Mikhail V Kalyakin, Yaroslav A Red'kin, Eugeny A Koblik, Paul DN Hebert

**Affiliations:** 1 Department of Integrative Biology, Biodiversity Institute of Ontario, University of Guelph, Guelph, Ontario, N1G 2W1, Canada; 2 Burke Museum of Natural History and Culture, University of Washington, Seattle, Washington 98195, USA; 3 Zoological Museum of Moscow Lomonosov University, Moscow, Russia

## Abstract

**Background:**

The Palearctic region supports relatively few avian species, yet recent molecular studies have revealed that cryptic lineages likely still persist unrecognized. A broad survey of cytochrome *c *oxidase I (COI) sequences, or DNA barcodes, can aid on this front by providing molecular diagnostics for species assignment. Barcodes have already been extensively surveyed in the Nearctic, which provides an interesting comparison to this region; faunal interchange between these regions has been very dynamic. We explored COI sequence divergence within and between species of Palearctic birds, including samples from Russia, Kazakhstan, and Mongolia. As of yet, there is no consensus on the best method to analyze barcode data. We used this opportunity to compare and contrast three different methods routinely employed in barcoding studies: clustering-based, distance-based, and character-based methods.

**Results:**

We produced COI sequences from 1,674 specimens representing 398 Palearctic species. These were merged with published COI sequences from North American congeners, creating a final dataset of 2,523 sequences for 599 species. Ninety-six percent of the species analyzed could be accurately identified using one or a combination of the methods employed. Most species could be rapidly assigned using the cluster-based or distance-based approach alone. For a few select groups of species, the character-based method offered an additional level of resolution. Of the five groups of indistinguishable species, most were pairs, save for a larger group comprising the herring gull complex. Up to 44 species exhibited deep intraspecific divergences, many of which corresponded to previously described phylogeographic patterns and endemism hotspots.

**Conclusion:**

COI sequence divergence within eastern Palearctic birds is largely consistent with that observed in birds from other temperate regions. Sequence variation is primarily congruent with taxonomic boundaries; deviations from this trend reveal overlooked biological patterns, and in some cases, overlooked species. More research is needed to further refine the taxonomic status of some Palearctic birds, but large genetic surveys such as this may facilitate this effort. DNA barcodes are a practical means for rapid species assignment, although efficient analytical methods will likely require a two-tiered approach to differentiate closely related pairs of species.

## Background

DNA barcoding employs sequences from a short standardized gene region to identify species [1]. The mitochondrial gene cytochrome *c *oxidase I (COI) has been firmly established as the core barcode region for animals [2] and its performance has been evaluated in birds from several regions, including North America [3], Brazil [4,5], Argentina [6], and Korea [7]. While most bird species are readily identifiable through morphological traits [8], their well-developed taxonomy makes them a valuable group to test the efficacy of barcoding. Additionally, avian taxonomy is not immune to change, and in recent decades DNA evidence has clarified many species boundaries. Broad surveys, such as DNA barcoding, can expedite this process by quickly spotlighting species that merit further taxonomic investigation [9-11]. This capacity is illustrated by several recently described species that were earlier revealed as divergent lineages during barcode surveys [12-14].

Although the avian diversity of the Palearctic is relatively depauperate [15] and its taxonomy was stable for decades, modern molecular techniques have spurred the recognition of overlooked species [16]. These new species were often hidden within morphologically cryptic assemblages, which impeded their discovery [e.g. [17,18]]. In other cases, biological species hypotheses could not be tested because divergent populations had allopatric distributions [19-21]. Molecular analyses continue to illuminate the phylogeographic structure of birds in this region [20,22-28]. A recent barcoding survey of Scandinavian birds by Johnsen *et al*. [29] revealed high species resolution plus a few divergent lineages, including some between European and North American populations of trans-Atlantic species. The Atlantic Ocean serves as a relatively impermeable barrier to dispersal for non-pelagic birds [15,30], but the situation is very different in the eastern Palearctic, where intercontinental exchange across the Bering Strait is more frequent [19,24,31]. Johnsen *et al*. [29] also highlighted sequence divergences within a few species that failed to correspond to known subspecies or logical geographical patterns - a pattern not observed in a comprehensive survey of Nearctic birds [3]. To determine if this pattern is recurrent, to highlight further cases of cryptic divergences, and to explore general patterns in sequence divergence, we advance COI barcode coverage in this study to include the breeding birds of the eastern Palearctic region, including Russia, Ukraine, Kazakhstan, and Mongolia.

Despite the growth of DNA barcode libraries, no consensus has yet emerged on the best method to analyze DNA barcode data [32]. Some of the original tools proposed to delimit species using COI sequences, such as neighbour-joining profiles [33] and distance thresholds [34], have been criticized by several authors for not realistically addressing the complexity of species boundaries [35-38]. More recent tools have gained complexity, incorporating coalescent theory and more elaborate statistical methods, though at the cost of computational time and power [38-40]. The situation is further complicated by the dual purposes proposed for barcoding: species identification and species discovery [41]. The majority of new generation tools require pre-defined species designations and consequently cannot be used to identify divergent genetic lineages within known groups. Although the use of DNA barcodes to "discover" species is contentious, it is generally accepted that barcode data can be used to flag potentially distinct taxa for further hypothesis testing [42]. Because the taxonomy of Holarctic birds is relatively mature [35], we take this opportunity to compare and contrast some of the more commonly used analytical methods.

## Methods

### Sampling

**Figure 1:**
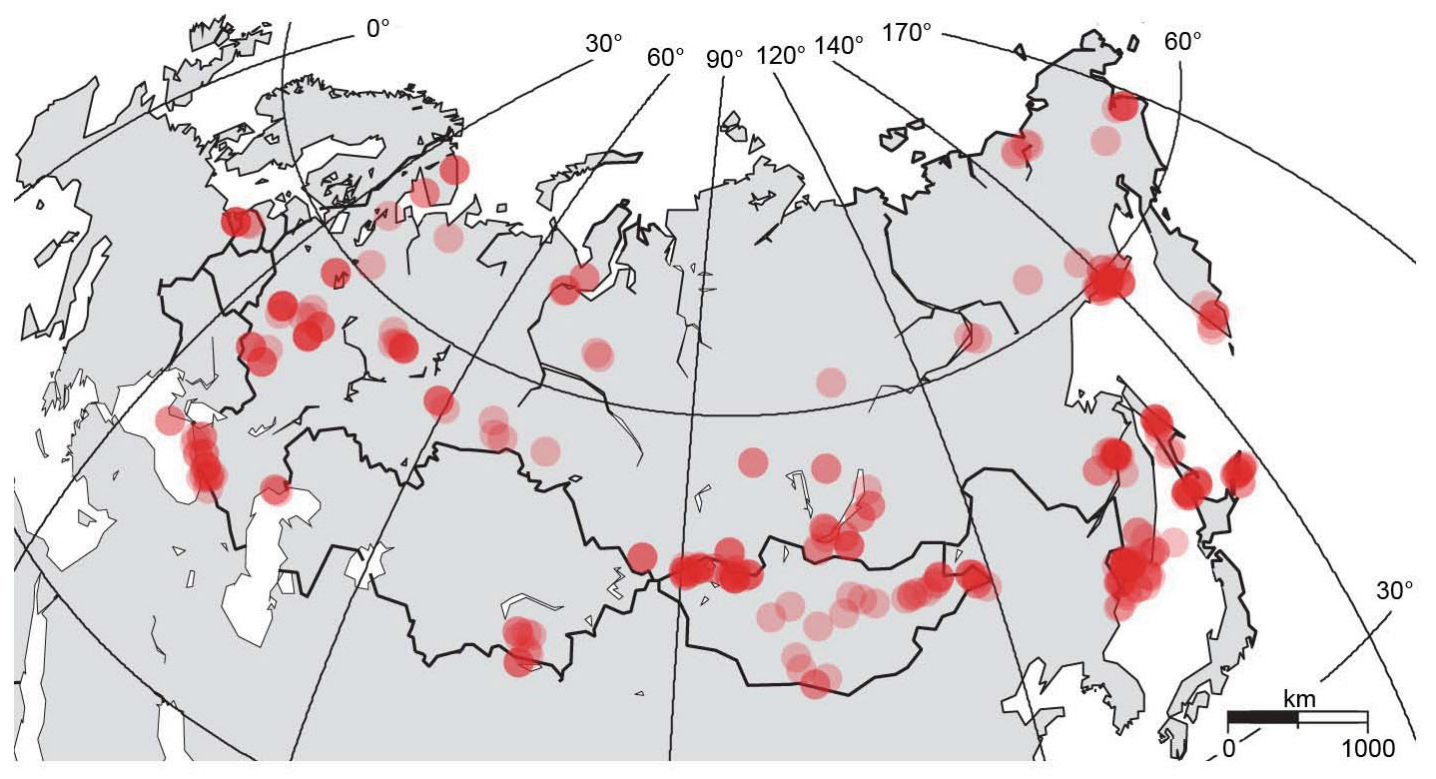
**Distribution of collecting sites**. Map of the eastern Palearctic region detailing the collecting sites for all specimens used in this study. Red circles indicate sampling sites. Sampling intensity is indicated by the brightness of each circle.

We examined 1,674 individuals representing 398 Palearctic species, with 83% of these taxa represented by multiple individuals. Species coverage was not uniformly distributed across orders and families due to specimen availability; nearly two-thirds of resident passerines were represented, versus less than 38% of non-passerine birds. We used frozen tissue (typically pectoral muscle) from museum specimens; all but six tissues were linked to vouchered specimens. All tissue specimens originated from either the ornithology collection at the Burke Museum of Natural History and Culture (87.5%) or from the Zoological Museum of Moscow University (12.5%), and were collected in the field during the past 20 years. To capture geographical variation, individuals collected from widely dispersed sites were preferentially sampled for each species whenever possible (see Figure 1 for distribution of collecting sites). Additional sequences from North American congeners were also contributed (see below). As a taxonomic reference, we followed Clements [43], including corrections and updates up to 8 October 2007 with the exception of treating *Corvus cornix *as conspecific with *C. corone *[*sensu *[44]].

### Laboratory methods

DNA extraction, PCR, and sequencing reactions follow the procedures described in Kerr et al. [6]. Only sequences greater than 500 bp and containing fewer than 10 ambiguous base calls were included in analyses. The sequence from one *Anas crecca *specimen was omitted from analysis due to suspicion that it was actually an *A. crecca *× *A. carolinensis *hybrid based on morphology and molecular results. Collection data, sequences, and trace files are available from the project 'Birds of the eastern Palearctic' at http://www.barcodinglife.org. All sequences have also been deposited in GenBank (Accession nos GQ481247 - GQ482920). A complete list of the museum catalog numbers, BOLD process identification numbers, and GenBank accession numbers for each specimen analyzed is included in Additional file 1.

We supplemented the data gathered in this study with sequences from North American congeners (accessible from the "Birds of North America - Phase II" project folder at http://www.barcodinglife.org) to examine divergences within transcontinental species and between sister species pairs. This added 849 sequences from 227 species, of which 66 species were shared with the Palearctic dataset. A list of BOLD process identification numbers and GenBank accession numbers for these sequences are listed in the Additional file 2. In total, 2,523 sequences from 559 species were included in the analyses.

### Data analysis

To assess the discriminatory power of COI barcodes, we compared three different methods commonly deployed in DNA barcoding studies: neighbour-joining (NJ) clusters, distance-based thresholds, and character-based assignment. We avoided more computationally intensive methods in favour of programs that could be executed in real time. For the clustering method, we used MEGA version 3.1 [45] to construct an NJ tree using the Kimura 2 parameter distance model (K2P). More sophisticated tree-building methods exist, but since we are concerned about terminal branches, not deeper branching patterns, this method is sufficient. Support for monophyletic clusters was determined using 500 bootstrap replicates. Species were accepted as being monophyletic providing they comprised the smallest diagnosable cluster with greater than 95% bootstrap support [46]. Though bootstrap support cannot be determined for species represented by a single sequence, they were included in the analysis to observe if they created paraphyly in neighbouring taxa. Species that could be divided into two or more well-supported clusters were flagged as potentially cryptic taxa.

**Figure 2:**
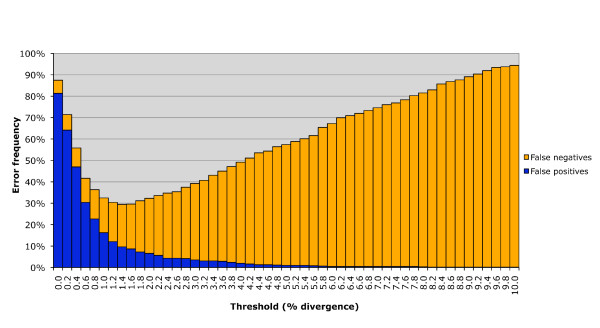
**Cumulative error plots**. Cumulative error plots of type I (false positive) and type II (false negative) errors for different divergence thresholds. Plot is based on 979 Holarctic bird species. The optimum threshold occurs at 1.6% divergence.

For the threshold-based approach, we blindly grouped sequences into provisional species clusters using a molecular operational taxonomic unit (MOTU) assignment program originally developed for nematodes [47]. The program, 'MOTU_define.pl' v2.07 (R. Floyd and M. Blaxter, unpublished; available from http://www.nematodes.org/bioinformatics/MOTU/index.shtml), clusters sequences together based on BLAST similarity using a user-defined base difference cut-off. Rather than use an arbitrary cut-off value, we determined the optimum threshold, or OT [36], by pooling our new data with the published North American bird dataset [3] and generating a cumulative error plot using all species with multiple representatives (see Figure 2). We adopted a liberal threshold of 11 base differences based on this result, which approximately equates to 1.6% divergence. Program parameters only included sequences greater than 500 bp with a minimum alignment overlap of 400 bp; however, this did not exclude any sequences from analysis.

For the character-based identification method, we used the character assignment system CAOS, which automates the identification of conserved character states (in this case, different nucleotides) from a tree of pre-defined species [48]. The system comprises two programs: P-Gnome and P-Elf [48]. P-Gnome is used to identify the diagnostic sequence characters that separate species and uses them to generate a rule set for species identification; P-Elf classifies new sequences to species using the rule set. We used the programs PAUP v4.0b10 [49] and MESQUITE v2.6 [50] respectively to produce the input NJ trees and nexus files for P-Gnome in accordance with the CAOS manual. We executed P-Gnome using several subsets of our data. First, we tried all of the Palearctic species included in this study to determine if diagnostic characters could be identified to separate a wide range of species. The input tree for P-Gnome requires that all species nodes be collapsed to single polytomies, which is an arduous task for large numbers of species. We only used a single representative from each species to circumvent this issue with the drawback that intraspecific variation is ignored during rule generation. To test the character-based method on a finer scale, we ran the program independently on the three largest genera sampled: *Emberiza *(n = 23), *Phylloscopus *(n = 13), and *Turdus *(n = 13). For species with multiple representatives, the shortest sequence was omitted from rule generation and used later to test species assignment.

For the first two tests (NJ and MOTU), all species exhibiting type I error, wherein a single species produced two or more discernable clusters of sequences, were compiled. Additional lines of evidence (e.g. alternative genes, morphological differences, song differences, etc.) were sought from previous studies to support or refute the likelihood of species differences in such cases. However, no formal recommendations are made here. We also performed the two-cluster test using Lintre [51] to determine if sequences from these species had evolved in a clock-like manner. For type II errors, wherein multiple species grouped together to form one well-supported cluster, sequences from each cluster were run through P-Gnome to ascertain if diagnostic characters could be identified that distinguish these close species.

## Results

### Neighbour-joining clusters

**Figure 3:**
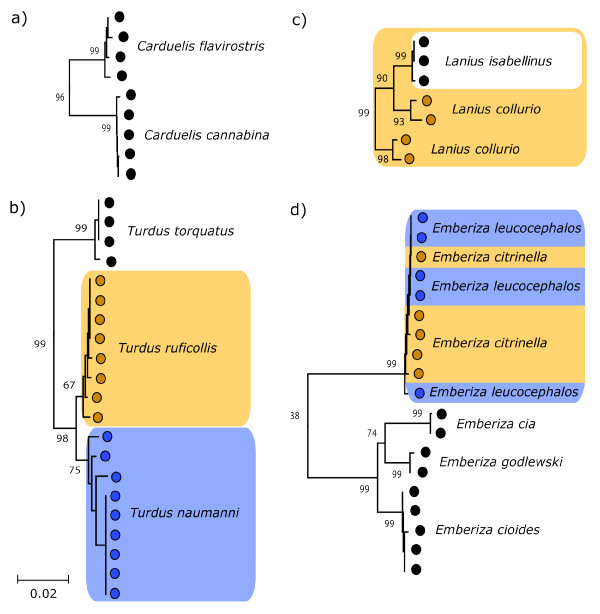
**Divergence patterns of closely related species**. Examples of divergence patterns illustrated in the NJ tree a) Species are monophlyletic with > 95% bootstrap support, b) Species are monophyletic, but is support is weak, c) Species are not monophyletic (i.e. paraphyly occurs), d) Multiple species form a single monophyletic group.

Of the 559 species analyzed, 72 had only a single representative and thus no bootstrap support could be calculated. However, all of these formed independent branches on the NJ tree that did not compromise the identification of other species. The remaining species were categorized into four patterns (Figure 3). Ninety percent formed well-supported (> 95% bootstrap) monophyletic groups (Figure 3a), and an additional 4% were monophyletic but with less than 95% bootstrap support (Figure 3b). Ten species, 2% of the total, were paraphyletic (*Larus canus, Thalasseus sandviciensis, Motacilla citreola, M. flava, Saxicola maurus, Sitta europaea, Certhia familiaris, Lanius collurio, L. excubitor*, and *Pica pica*)(Figure 3c). The remaining taxa (4%) formed monophyletic clusters that contained two or more species (Figure 3d; Table 1). These were mostly limited to pairs of sister taxa, with the notable exception of one cluster containing 10 species in the Herring gull complex (*Larus californicus, L. fuscus, L. glaucescens, L. glaucoides, L. heuglini, L. hyperboreus, L. occidentalis, L. smithsonianus, L. thayeri*, and *L. vegae*).

**Table 1:** Species with limited COI divergence

	Family	Species	n	NJ	Bootstrap	Inter sp	CAOS
1	Gaviidae	*Gavia adamsii*	6	b	38	0.77	Yes
		*Gavia immer*	3		67		

2	Phalacrocoracidae	*Phalacrocorax pelagicus*	9	b	61	0.78	Yes
		*Phalacrocorax urile*	1		n/a		

3	Ardeidae	*Ardea cinerea*	1	b	n/a	1.90	Yes
		*Ardea herodias*	4		99		

4	Anatidae	*Anas falcata*	1	b	n/a	1.46	Yes
		*Anas strepera*	9		50		

5		*Aythya affinis*	9	b	24	1.58	Yes
		*Aythya americana*	10		61		
		*Aythya collaris*	10		81		
		*Aythya fuligula*	3		90		
		*Aythya marila*	11		12		
		*Aythya valisineria*	6		87		

6		*Bucephala clangula*	7	b	55	1.58	Yes
		*Bucephala islandica*	10		87		

7		*Somateria fishcheri*	7	b	94	0.96	Yes
		*Somateria mollisima*	10	d	nm		
		*Somateria spectabilis*	3		nm		

8	Phasianidae	*Coturnix coturnix*	2	a	99	1.50	Yes
		*Coturnix japonica*	4		99		

9	Accipitridae	*Buteo buteo*	3	b	85	1.92	Yes
		*Buteo lagopus*	2		92		

10	Scolopacidae	*Gallinago delicata*	6	d	nm	0.15	No
		*Gallinago gallinago*	4		nm		

11		*Gallinago megala*	2	b	93	0.61	Yes
		*Gallinago stenura*	5		98		

12	Glareolidae	*Glareola pratincola*	2	a	99	1.61	Yes
		*Glareola nordmanni*	3		99		

13	Laridae	*Larus canus*	5	b	89	0.65	Yes
		*Larus canus "brachyrhynchus"*	4		77		
		*Larus delawarensis*	3		50		
		*Larus marinus*	3		87		
		*Larus spp*.†	34	d	nm	0.24	No

14	Alcidae	*Cepphus carbo*	3	a	99	0.97	Yes
		*Cepphus columba*	2		99		

15	Cuculidae	*Cuculus canorus*	5	d	nm	0.71	No
		*Cuculus optatus*	5		nm		

16	Motacillidae	*Motacilla flava "taivana"*	2	b	99	1.16	Yes
		*Motacilla citreola "citreola"*	2		87		
		*Motacilla citreola "werae"*	4		98		

17	Turdidae	*Turdus naumanni*	9	b	75	1.10	Yes
		*Turdus ruficollis*	8		67		

18		*Turdus chrysolaus*	9	b	97	1.35	Yes
		*Turdus obscurus*	5		67		
		*Turdus pallidus*	4		51		

19	Laniidae	*Lanius isabellinus*	3	b	99	1.71	Yes
		*Lanius collurio*‡	2		93		

20	Fringillidae	*Carduelis flammea*	10	d	nm	0.40	No
		*Carduelis hornemanni*	6		nm		

21		*Carduelis pinus*	6	a	99	2.01	Yes
		*Carduelis spinus*	15		99		

22	Emberizidae	*Emberiza citrinella*	5	d	nm	0.09	No
		*Emberiza leucocephalos*	5		nm		

List of all groups of species that failed recognition via MOTU analysis. Additionally, species with aberrant NJ profiles are indicated; profile designations (a-d) refer to Figure 3. Bootstrap support is given for each species ("nm" denotes that the species is not monophyletic) and the average interspecific distance is given for each group of species, both as percentages. Whether groups could be distinguished via CAOS is also indicated.

† Represents the ten members of the Herring gull complex listed in the text.

‡ Only two of four specimens of the paraphyletic *Lanius collurio *exhibited limited divergence from *L. isabellinus*.

Forty-two species showed evidence of having divergent lineages (Table 2). Twenty-two species formed two or more well-supported (> 95% bootstrap) monophyletic clusters. Another four species formed two distinct clusters, but with one cluster possessing only 90-94% bootstrap support. These cases included 7 of the 10 paraphyletic species. In an additional 16 species, a single specimen was divergent from the rest, but further sampling is necessary to adequately evaluate these cases. Table 2 lists all species with divergent lineages. The total number of species recognized via this method is difficult to gauge due to inclusion of single representatives for some species and divergent lineages.

**Table 2:** Species bearing divergent COI lineages

Species	NJ	MOTU	n	Bootstrap	Dist	Phyl	Bio	Ref
*Falco columbarius*	?	*	1/4	-/99	2.29	P/N	A	
*Gallinula chloropus*	?	*	1/6	-/99	3.45	P/N	A	[29]
*Charadrius alexandrinus*	*	*	4/3	99/99	7.53	P/N	A	
*Tringa totanus*	*		3/3	99/99	0.87	E/W	A	[35]
*Numenius phaeopus*	?	*	5/1	99/-	3.57	P/N	A	[19]
*Limosa limosa*	?	*	4/1	99/-	2.27	E/W	P	
*Thalasseus sandvicensis*	*	*	2/6	98/99	3.78	P/N	A	[81]
*Streptopelia orientalis*	?	*	5/2	99/94	2.14	Sak	P	
*Asio otus*	?		4/5	99/94	1.10	P/N	A	
*Aegolius funereus*	?	*	1/3	-/99	4.13	P/N	A	[82]
*Caprimulgus europaeus*	?	*	3/1	99/-	2.97	Cau	A	
*Dendrocopos major*	?	*	4/1	99/-	2.71	Sak	A	[66]
*Alauda arvensis*	*	*	1/4/5	99/99/99	6.02	E/W, Sak	A/P	
*Delichon dasypus*	?	*	1/1/2	-/-/99	3.58		S	
*Anthus rubescens*	*	*	6/2	99/99	2.46	P/N	A	[19]
*Motacilla flava*		*	2/1	87/-	5.57	E/W	A	[23]
*Troglodytes troglodytes*	*	*	3/8/1/5/2	99/99/-/99/99	3.70	E/W, Cau P/N	A	[22]
*Erithacus rubecula*	?	*	6/1	99/-	4.66	Cau	A	
*Luscinia megarhynchos*	?	*	1/2	-/99	2.56	Cau	A	
*Muscicapa sibirica*	?	*	6/1	99/-	2.85	Sak	A	
*Phoenicurus auroreus*	*	*	2/3	99/99	2.36	E/W	A	
*Phoenicurus ochruros*	*	*	3/2/1	99/99/-	3.66	E/W, Cau	A	
*Phoenicurus phoenicurus*	*	*	2/4	99/99	5.20		S	[29]
*Saxicola maurus*	?	*	7/1	99/-	7.91	E/W	A	[83]
*Cettia diphone*	*	*	10/2	99/97	3.03	Sak	A	
*Phylloscopus borealis*	*	*	8/6	99/99	3.59	Sak	A	[31]
*Phylloscopus trochiloides*	*	*	4/4	99/99	4.39	E/W	A	[84]
*Sylvia curruca*	*	*	6/3	99/99	5.56	E/W	A	
*Urosphena squameiceps*	?	*	4/1	99/-	2.09	Sak	A	
*Regulus regulus*	*	*	7/3	99/99	3.69	E/W	A	[85]
*Parus major*	*	*	6/7	99/99	2.59	E/W	A	[28,86]
*Periparus ater*	*	*	8/3	99/99	4.43	Cri	A	[29]
*Sitta europaea*	?	*	1/10/1/1	-/99/-/-	2.91	E/W, Cau, Yak	A	[26]
*Certhia familiaris*	?	*	6/3	93/99	1.93	E/W	A	
*Lanius excubitor*	*	*	2/4	99/99	3.60	P/N	P	
*Lanius collurio*	?	*	2/2	93/98	2.29	E/W	A	
*Corvus corone*		*	1/7	-/83	2.15	E/W	A	
*Corvus frugilegus*	*	*	2/2	99/99	2.94	E/W	A	[44]
*Garrulus glandarius*	*	*	4/3	99/99	2.63	E/W	A	[87]
*Pica pica*	?	*	1/9	-/99	3.59	E/W	A	[44]
*Sturnus vulgaris*	?	*	5/1	-/96	1.85	Kaz	A	
*Pinicola enucleator*	*	*	12/2	99/99	4.54	P/N	A	[29]
*Emberiza pallasi*	*	*	4/2	99/99	3.10	Mog	A	
*Emberiza spodocephala*	*	*	8/6	99/99	3.36	Sak	A	

List of all species containing divergent COI lineages. An asterisk in the respective column indicates that lineages were supported via the NJ or MOTU method (a question mark indicates undetermined cases). The number of specimens and bootstrap support (%) for each cluster is indicated, as is the mean distance (%) between all clusters within each species.

Phyl: Phylogeographic patterns (P/N = Palearctic/Nearctic, E/W = east/west, Sak = Sakhalin region, Cau = Caucasus region, Cri = Crimean region, Kaz = Kazakhstan, Mog = Mongolia, Yak = Sakha (Yakutia) region)

Bio: Biogeographic patterns (A = allopatric, P = parapatric, S = sympatric)

Additional references detailing more comprehensive studies are supplied where available.

### Distance-based assignment

The MOTU analysis identified 570 clusters, or taxonomic units, versus the 559 recognized by traditional taxonomy. The similarity of these numbers disguises discrepancies in species assignment. Poor resolution occurred in 22 groups representing 61 species (Table 1). These lumped taxa, as with the NJ clustering method, were mostly limited to pairs of species, save for two triplets (*Somateria spp*. and *Turdus spp*.) and thirteen large white-headed gulls (*Larus canus, L. delawarensis, L. marinus*, and the aforementioned members of the Herring gull complex). Divergent groups were recognized in 42 species (Table 2); 95% of these overlapped with those recognized via NJ. Most were divided into two clusters, though three or more clusters were detected in five species. In two of the paraphyletic species (*Motacilla flava, Lanius collurio*), one lineage was lumped with a closely related species while the other lineage was divergent.

### Character-based assignment

P-Gnome failed to produce a diagnostic rule set that that could distinguish all 398 species sequenced in this study. Results using subsets of the data were more successful. Complete diagnostic rule sets were generated and successfully tested for both *Phylloscopus *and *Turdus*. The rule set for *Emberiza *could not distinguish between sequences of E. *leucocephalos *and *E. citrinella *due to their near congruence. In addition, P-Elf failed to correctly identify single sequences from the species *E. chrysophrys *and *E. elegans*. The former sequence was short (594 bp) and might have lacked important diagnostic characters. However, the latter sequence was of typical length (694 bp) and only exceptional in that it contained 5 polymorphic sites from the sequence used to generate the rule set. Both of these species were incorrectly identified as *E. aureola*, though this identification would vary if the input tree were altered.

Of 22 groups of lumped species, all but five could be resolved using diagnostic characters (see Table 1). For example, the species pair *Coturnix coturnix *and *C. japonica *possessed 10 diagnostic nucleotide sites, two short of recognition by the MOTU threshold but still easily distinguishable. More complex rule sets were required when more species were involved (e.g. *Aythya *ducks). The remaining groups featured virtually no variation between species. These include 10 members of the herring gull complex (*Larus *spp.) and the species pairs *Gallinago gallinago/G. delicata*, *Cuculus canorus*/*C. optatus*, *Carduelis flammea*/*C. hornemanni*, and *Emberiza citrinella/E. leucocephalos*.

## Discussion

### Species boundaries in Palearctic Birds

Divergence levels between closely related species were highly variable, ranging from approximately 0-16%; however, some of these values may be inflated for under-sampled genera and families. Recent studies have detached rate variation in the mitochondrial genome from factors such as population size, body size, and other life-history traits [52-54]. While some authors contend that rate variation in birds is highly irregular [53], a recent thorough review demonstrated relatively minor variation and upheld the occurrence of clock-like evolution [55]. Consequently, we attribute the limited divergence between some sister species to recent speciation events. Studies documenting recent and rapid diversifications often address subspecific variants rather than full species [56,57]. Still, low sequence divergence does not necessarily indicate that species should be synonymised [58]. Low sequence divergence is particularly common in superspecies complexes, including those divided between continents, but the species within them remain valid units for both ecological studies and conservation.

Four species pairs and the large white-headed gulls included in this study featured virtually no variation for COI and could not be distinguished using any of the approaches employed in this study. Low divergence in mitochondrial markers had been previously demonstrated in each of these cases. Lumping has been considered for some, including *Carduelis flammea/hornemanni *[59] and the recently split *Gallinago gallinago/delicata *[35], but more evidence is required. The cause of shared mitochondrial haplotypes between *Cuculus canorus *and *C. optatus *has not been resolved (hybrids have never been documented [60]), but their taxonomic distinction has been asserted based on song differences [61]. *Emberiza citrinella *and *E. leucocephalos *are exceptionally interesting in that they are the most phenotypically distinct of these pairs and a survey of nuclear markers revealed genetic divergence [62]. They are known to hybridize extensively and introgression is a likely explanation [62]. Species boundaries in the large white-headed gulls may have also been confused by contemporary hybridization, though shallow history and slowed rates of evolution have also been implicated [63,64].

Nearly one tenth of the species (7.5%) analyzed in this study contained divergent mitochondrial lineages, with divergences averaging 3.6%. While divergence at a single mitochondrial gene alone is insufficient evidence to define new species boundaries, it is cause for new hypothesis testing. Several recently split species that are morphologically similar to their nearest relative, such as the swallow *Riparia diluta *and the warbler *Locustella amnicola*, represent taxa that barcodes would flag for closer scrutiny. Distributions of most of the divergent lineages in this study conform to one of four previously documented phylogeographic trends (summarized in Table 2): a unique lineage in the Caucasus region [65]; a unique lineage in the Sakhalin region [66]; divergent lineages divided into eastern and western populations [25]; divergent lineages on either side of the Bering Strait [19]. Species with multiple lineages can display more than one of these patterns. A few lineages appear to be parapatric, which could indicate areas of overlap or hybrid zones [67]. Past climate change and its effect on historical habitat distribution is likely responsible for shaping patterns of genetic divergence in modern populations, but whether or not these populations were divided by the same historical events is difficult to determine without dating divergence times. While the COI sequences mostly appear to be evolving in a clocklike fashion, dating is risky given the absence of adequate calibration points and the reliance on various assumptions [24,55].

Most species exhibited surprisingly limited variation between Old World and New World populations. Of the approximately 140 species with Holarctic distributions, 43% are represented in this study. Only 11 of these 61 species (18%) possessed intraspecific divergences great enough to signal likely species-level differences by either the NJ or MOTU method. The Bering Sea has served a variable but clear role as a barrier to gene flow for birds, particularly non-marine species. Several trans-Beringian species have already been split in recent years, due partly to molecular evidence (e.g. *Brachyramphus marmoratus/B. perdix *[21], *Picoides tridactylus/P. dorsalis *[19], *Pica pica/P. hudsoni *[68]). Still, caution must be exercised when identifying species boundaries between allopatric populations. For example, one of the Palearctic *Lanius excubitor *specimens from this study appears to belong to the North American clade, suggesting that some modern exchange might occur between the continents. Though it is more common for Palearctic species to invade the Nearctic, the reverse pattern has also been observed [69]. Correct interpretation of this result requires further study with additional specimens.

This survey has identified a number of species that demand further taxonomic scrutiny (see Table 2). It is likely that some of the divergent lineages identified here represent distinct species. Of course, genetic distances do not always correspond to species limits [19,69]. Alternative explanations for the divergent lineages observed include historical phylogeographic isolation, female-restricted dispersal, or male-biased gene flow [35]. The common phylogeographic patterns observed in many of the divergent lineages support the idea of historical isolation. Areas of secondary contact must be further studied to evaluate the gene flow between lineages [70]. In a few exceptional cases genetic lineages appear largely sympatric, including within *Alauda arvensis*, *Delichon dasypus*, and *Phoenicurus phoenicurus*. Nuclear copies of mitochondrial sequences (numts) are an unlikely explanation given the absence of stop codons and heterozygous peaks. *Phoenicurus phoenicurus *was also noted by Johnsen *et al*. [29], who attributed the aberrant phylogeographic pattern to admixture of historically separated lineages. This situation is paradoxical compared to suspected introgressed genomes used to explain limited divergence in sister species. Selective sweeps are frequently invoked to explain the limited variation observed in mitochondrial markers [6,71], which raises the question of how two mtDNA lineages manage to persist in one species but not another. Ongoing research of species limits and evolutionary histories is clearly still necessary in the Palearctic.

### Methods comparison

The MOTU assignment program used in this study was originally developed for meiofauna with few morphological characters [47]. Applying it to a group with better-established taxonomy allows more conclusive tests of its performance. Our results indicated a type II error rate of 10.9%, but this is inflated by the diversity of named white-headed gull species (*Larus spp*.); with these species eliminated, error is reduced to 8.8%. At this point, we don't consider type I errors a fault of this method since these cases are biologically interesting, do not necessarily impair identification, and may represent over-looked species [34,35]. The major drawback to the program in its current form is the difficulty in associating any level of statistical support with species assignments, which may differ slightly depending on the input order of sequences. Although the program does allow a random re-sampling scheme, the output is not summarized, making statistical inference on the stability of taxonomic units virtually impossible. The major impediment now for biologists applying this method to microscopic invertebrates still lies in determining an operational threshold.

The use of a distance-based threshold technique has been a major point of contention in the DNA barcoding endeavour [37,72,73]. While COI variation represents a product of evolution, an arbitrary cut-off value does not reflect what is known about the evolutionary processes responsible for this variation. The threshold approach depends on the existence of a gap between levels of intraspecific variation and interspecific divergence, which opponents argue does not exist. Early success in identifying a "barcoding gap" in North American birds was attributed to insufficient sampling of closely related species [35,37]. We found the original "10× rule" proposed by Hebert et al. [34] to be too conservative to recognize recently diverged species and opted for a more liberal threshold of 1.6%. While this value was more effective at species identification, some sister species exhibited little or no variation, which eliminates the possibility of identifying a gap. However, invalidating the use of distance-based methods based on the failure of thresholds might be going too far. Identifying the nearest matches to a query sequence is still useful, even if a conclusive assignment is not provided [74].

The development of an NJ profile for identification depends on the coalescence of species and not an arbitrary level of divergence [36]; in theory, species that failed recognition via the threshold approach may still be recognized. However, we found that the same species were typically problematic for both approaches (see Table 1). This is not surprising: high bootstrap support is unlikely when a slight aberration in the data would alter the results [75], which is the case when sequences are highly similar. Critics have argued that the bootstrap test for monophyly is simply too conservative and incorrectly rejects monophyly in too many cases [76]. This is apparent from the 4% of species that appear monophyletic but with limited support. Alternative forms of statistical support based on coalescent theory suggest that increased sampling decreases the risk of monophyly by chance, which would support the reality of these patterns despite low bootstrap values [77]. A modified NJ algorithm with non-parametric bootstrapping has been proposed to offer fast barcode-based identifications, but success still depends on the completeness of the reference database and weakly divergent species remain problematic [78].

The character-based method was effective, but did not feature the same scalability as the previous two methods. We found that the CAOS system was severely constrained by limits on the number of species that could be included for rule generation. More thorough benchmarking is necessary to determine the upper limits of the program, but at this point in time they are unclear. We also found that comprehensive sampling for each taxon is vital for accurate rules that account for intraspecific polymorphisms. When operating with smaller sets of taxa, the programs were successful in both identifying diagnostic characters and in subsequently identifying new sequences to species. However, we did find P-Elf to be highly susceptible to erroneous identifications for unrepresented species, counter to previous claims [79]. When using smaller datasets, sequences introduced from novel taxa were typically given a species level identification, even when those taxa derived from a different order (*data not shown*).

Both distance-based and clustering-based methods appear to share the same computational strengths, handling even large datasets quickly. However, both methods are also impaired by the same issues: limited divergence between sister taxa. The results of the character-based method appear to complement the former two methods. While it is precise and able to detect minor differences in closely related taxa [80], it is unable to handle large numbers of sequences. It is also susceptible to errors when the appropriate taxa have not been comprehensively sampled. When it comes to species identification, we propose that the best method might actually be a multi-tiered approach, where an initial method is used to narrow the identification to a select group of taxa and an alternate method is used to differentiate similar taxa. Similarly, Munch *et al*. [78] recommend incorporating methods that model population level variation to distinguish between closely allied species. For cases of limited divergence, sampling a longer stretch of COI or even alternative genes would increase support for identifications.

## Conclusion

The utility of DNA barcodes in avian research is two-fold. Preliminary investigations, such as this, offer fresh insight to aid the ongoing effort to refine avian taxonomy. And secondly, a comprehensive library of COI sequences provides an invaluable tool for species assignment when differences in morphology are difficult to measure or otherwise assess. This includes species with cryptic morphological differences (e.g. *Phylloscopus *warblers, *Calandrella *larks, and *Empidonax *flycatchers) but also scenarios where identification is desired but only fragmentary remains are available (e.g. air strikes, nest contents, diet analysis, etc.). This study reaffirms these possibilities, demonstrating that COI sequence variation is largely congruent with species boundaries. Departures from this congruence are typically indicative of overlooked biological processes; historically separated lineages in the case of within species divergence, and recent or historical gene flow in the case of shared haplotypes between species. Molecular analysis is novel for some of these taxonomic groups or geographic areas, and the resultant observations highlight areas in need of further taxonomic study.

The efficacy of DNA barcodes for use in species assignment is dependent on two factors: the construction of thorough COI libraries and efficient tools to assign sequences to species. This study substantiates the need for dense taxonomic sampling. It further demonstrates that standardized gene libraries are easily amalgamated to examine geographically broad areas or taxonomically diverse groups. Current analytical methods for barcode data appear insufficient for handling recently evolved species. Though less of a problem for known cases of shallow divergence, where pairs of species may often be further scrutinized using a multi-tiered approach, these cases may be more problematic for those who wish to use barcodes as a tool to accelerate species discovery in poorly studied groups.

## Competing interests

The authors declare that they have no competing interests.

## Authors' contributions

KCRK coordinated the study, carried out the molecular and statistical analyses, and drafted the manuscript. SMB and MVK provided specimens for the study, participated in its coordination, and helped with the manuscript. YAR and EAK contributed to the interpretation of the results and helped with the manuscript. PDNH conceived of the study, participated in its design and coordination, and helped with the manuscript. All authors read and approved the final manuscript.

## Supplementary Material

Additional file 1 **List of sampled specimens**. Complete list of museum accession numbers, BOLD process identification numbers, and GenBank accession numbers for each specimen analyzed in this study. Click here for file

Additional file 2 **List of sequences acquired from BOLD**. Complete list of BOLD process identification numbers and GenBank accession numbers for all sequences used in this study from the "Birds of North America - Phase II" project in BOLD. Click here for file
