# Supplementary material for: Filling the gap - COI barcode resolution in eastern Palearctic birds
Source: Front Zool. 2009 Dec 9;6:29. doi: 10.1186/1742-9994-6-29 (PMC2796652; doi:10.1186/1742-9994-6-29)
Supplement: Additional file 1 — List of sampled specimens. Complete list of museum accession numbers, BOLD process identification numbers, and GenBank accession numbers for each specimen analyzed in this study. [file 1742-9994-6-29-S1.DOC]

Table S1.

| **Species** | **Museum Catalog #** | **BOLD Process ID** | **GenBank Accession** |
| --- | --- | --- | --- |
| *Accipiter nisus* | UWBM 51182 | KBPBU065-06 | GQ481247 |
| *Accipiter nisus* | UWBM 66742 | KBPBU067-06 | GQ481248 |
| *Accipiter nisus* | UWBM 64662 | KBPBU066-06 | GQ481249 |
| *Accipiter nisus* | UWBM 59777 | KBPBU069-06 | GQ481250 |
| *Accipiter nisus* | UWBM 46858 | KBPBU068-06 | GQ481251 |
| *Accipiter virgatus* | UWBM 59914 | KBPBU072-06 | GQ481252 |
| *Accipiter virgatus* | UWBM 59853 | KBPBU071-06 | GQ481253 |
| *Acrocephalus agricola* | UWBM 46500 | KBPBR247-07 | GQ481254 |
| *Acrocephalus agricola* | UWBM 61230 | KBPBR279-07 | GQ481255 |
| *Acrocephalus bistrigiceps* | UWBM 47551 | KBPBR515-07 | GQ481256 |
| *Acrocephalus bistrigiceps* | UWBM 59967 | KBPBR189-07 | GQ481257 |
| *Acrocephalus bistrigiceps* | UWBM 74817 | KBPBR408-07 | GQ481258 |
| *Acrocephalus bistrigiceps* | UWBM 47067 | KBPBR164-07 | GQ481259 |
| *Acrocephalus dumetorum* | UWBM 49487 | KBPBR112-07 | GQ481260 |
| *Acrocephalus dumetorum* | UWBM 46319 | KBPBR154-07 | GQ481261 |
| *Acrocephalus dumetorum* | UWBM 75611 | KBPBR305-07 | GQ481262 |
| *Acrocephalus dumetorum* | UWBM 49254 | KBPBR081-07 | GQ481263 |
| *Acrocephalus dumetorum* | UWBM 49522 | KBPBR115-07 | GQ481264 |
| *Acrocephalus dumetorum* | UWBM 46384 | KBPBR200-07 | GQ481265 |
| *Acrocephalus dumetorum* | UWBM 73740 | KBPBR512-07 | GQ481266 |
| *Acrocephalus melanopogon* | UWBM 61476 | KBPBR455-07 | GQ481267 |
| *Acrocephalus orientalis* | UWBM 74814 | KBPBR407-07 | GQ481268 |
| *Acrocephalus orientalis* | UWBM 75559 | KBPBR372-07 | GQ481269 |
| *Acrocephalus orientalis* | UWBM 71718 | KBPBR240-07 | GQ481270 |
| *Acrocephalus orientalis* | UWBM 59985 | KBPBR190-07 | GQ481271 |
| *Acrocephalus orientalis* | UWBM 59966 | KBPBR188-07 | GQ481272 |
| *Acrocephalus palustris* | UWBM 49604 | KBPBR120-07 | GQ481273 |
| *Acrocephalus palustris* | UWBM 61165 | KBPBR276-07 | GQ481274 |
| *Acrocephalus palustris* | UWBM 49380 | KBPBR093-07 | GQ481275 |
| *Acrocephalus palustris* | UWBM 74205 | KBPBR308-07 | GQ481276 |
| *Acrocephalus palustris* | UWBM 64698 | KBPBR468-07 | GQ481277 |
| *Acrocephalus palustris* | UWBM 56670 | KBPBR176-07 | GQ481278 |
| *Acrocephalus schoenobaenus* | UWBM 59638 | KBPBR437-07 | GQ481279 |
| *Acrocephalus schoenobaenus* | UWBM 64789 | KBPBR476-07 | GQ481280 |
| *Acrocephalus schoenobaenus* | UWBM 82146 | KBPBR281-07 | GQ481281 |
| *Acrocephalus scirpaceus* | UWBM 56699 | KBPBR179-07 | GQ481282 |
| *Acrocephalus scirpaceus* | UWBM 56686 | KBPBR177-07 | GQ481283 |
| *Actitis hypoleucos* | UWBM 60137 | KBPBU263-06 | GQ481284 |
| *Actitis hypoleucos* | UWBM 46392 | KBPBU262-06 | GQ481285 |
| *Actitis hypoleucos* | UWBM 43824 | KBPBU260-06 | GQ481286 |
| *Actitis hypoleucos* | UWBM 49579 | KBPBU259-06 | GQ481287 |
| *Actitis hypoleucos* | UWBM 46623 | KBPBU261-06 | GQ481288 |
| *Aegithalos caudatus* | ZMMU 46a | KBPZM021-06 | GQ481289 |
| *Aegithalos caudatus* | ZMMU RYA 1776 | KBPZM202-07 | GQ481290 |
| *Aegithalos caudatus* | ZMMU RYA 1775 | KBPZM201-07 | GQ481291 |
| *Aegithalos caudatus* | ZMMU RYA 1774 | KBPZM200-07 | GQ481292 |
| *Aegithalos caudatus* | ZMMU 57a | KBPZM025-06 | GQ481293 |
| *Aegithalos caudatus* | ZMMU RYA 1780 | KBPZM204-07 | GQ481294 |
| *Aegolius funereus* | UWBM 52674 | KBPBU404-06 | GQ481295 |
| *Aix galericulata* | UWBM 72196 | KBPBU034-06 | GQ481296 |
| *Alauda arvensis* | ZMMU RYA 1277 | KBPZM107-07 | GQ481297 |
| *Alauda arvensis* | ZMMU RYA 1276 | KBPZM106-07 | GQ481298 |
| *Alauda arvensis* | ZMMU RYA 1180 | KBPZM098-07 | GQ481299 |
| *Alauda arvensis* | UWBM 47584 | KBPBU508-06 | GQ481300 |
| *Alauda arvensis* | UWBM 47555 | KBPBU507-06 | GQ481301 |
| *Alauda arvensis* | UWBM 47394 | KBPBU506-06 | GQ481302 |
| *Alauda arvensis* | UWBM 60017 | KBPBU502-06 | GQ481303 |
| *Alauda arvensis* | UWBM 49547 | KBPBU505-06 | GQ481304 |
| *Alauda arvensis* | UWBM 44324 | KBPBU504-06 | GQ481305 |
| *Alauda arvensis* | UWBM 51731 | KBPBU503-06 | GQ481306 |
| *Alcedo atthis* | ZMMU 77a | KBPZM039-06 | GQ481307 |
| *Alcedo atthis* | UWBM 46394 | KBPBU438-06 | GQ481308 |
| *Alcedo atthis* | UWBM 47192 | KBPBU440-06 | GQ481309 |
| *Alcedo atthis* | UWBM 61142 | KBPBU441-06 | GQ481310 |
| *Alcedo atthis* | UWBM 59855 | KBPBU439-06 | GQ481311 |
| *Alcedo atthis* | UWBM 51169 | KBPBU442-06 | GQ481312 |
| *Alectoris chukar* | UWBM 46402 | KBPBU126-06 | GQ481313 |
| *Alectoris chukar* | UWBM 46516 | KBPBU127-06 | GQ481314 |
| *Alectoris chukar* | UWBM 57857 | KBPBU125-06 | GQ481315 |
| *Alectoris chukar* | UWBM 57853 | KBPBU124-06 | GQ481316 |
| *Anas acuta* | UWBM 59540 | KBPBU035-06 | GQ481317 |
| *Anas crecca* | UWBM 59546 | KBPBU039-06 | GQ481318 |
| *Anas crecca* | UWBM 56971 | KBPBU038-06 | GQ481319 |
| *Anas crecca* | UWBM 56970 | KBPBU037-06 | GQ481320 |
| *Anas crecca* | UWBM 61324 | KBPBU040-06 | GQ481321 |
| *Anas falcata* | UWBM 46872 | KBPBU041-06 | GQ481322 |
| *Anas penelope* | UWBM 44651 | KBPBU043-06 | GQ481323 |
| *Anas penelope* | UWBM 59450 | KBPBU042-06 | GQ481324 |
| *Anas platyrhynchos* | UWBM 47160 | KBPBU044-06 | GQ481325 |
| *Anas querquedula* | UWBM 47172 | KBPBU045-06 | GQ481326 |
| *Anas strepera* | UWBM 56676 | KBPBU047-06 | GQ481327 |
| *Anas strepera* | UWBM 56499 | KBPBU046-06 | GQ481328 |
| *Anser albifrons* | UWBM 43977 | KBPBU023-06 | GQ481329 |
| *Anthus campestris* | UWBM 66449 | KBPBU565-06 | GQ481330 |
| *Anthus campestris* | UWBM 61361 | KBPBU562-06 | GQ481331 |
| *Anthus campestris* | UWBM 67582 | KBPBU563-06 | GQ481332 |
| *Anthus campestris* | UWBM 66596 | KBPBU564-06 | GQ481333 |
| *Anthus campestris* | UWBM 66466 | KBPBU566-06 | GQ481334 |
| *Anthus cervinus* | UWBM 44314 | KBPBU569-06 | GQ481335 |
| *Anthus cervinus* | UWBM 44498 | KBPBU567-06 | GQ481336 |
| *Anthus cervinus* | UWBM 51551 | KBPBU568-06 | GQ481337 |
| *Anthus godlewskii* | UWBM 46331 | KBPBU576-06 | GQ481338 |
| *Anthus godlewskii* | UWBM 66704 | KBPBU575-06 | GQ481339 |
| *Anthus godlewskii* | UWBM 67633 | KBPBU574-06 | GQ481340 |
| *Anthus godlewskii* | UWBM 57998 | KBPBU573-06 | GQ481341 |
| *Anthus godlewskii* | UWBM 59902 | KBPBU572-06 | GQ481342 |
| *Anthus gustavi* | UWBM 75486 | KBPBU769-06 | GQ481343 |
| *Anthus gustavi* | UWBM 43951 | KBPBU578-06 | GQ481344 |
| *Anthus gustavi* | UWBM 43958 | KBPBU577-06 | GQ481345 |
| *Anthus hodgsoni* | UWBM 47552 | KBPBU582-06 | GQ481346 |
| *Anthus hodgsoni* | UWBM 60056 | KBPBU579-06 | GQ481347 |
| *Anthus hodgsoni* | UWBM 56521 | KBPBU583-06 | GQ481348 |
| *Anthus hodgsoni* | UWBM 59812 | KBPBU580-06 | GQ481349 |
| *Anthus pratensis* | UWBM 59611 | KBPBU588-06 | GQ481350 |
| *Anthus pratensis* | UWBM 49662 | KBPBU584-06 | GQ481351 |
| *Anthus pratensis* | UWBM 56759 | KBPBU587-06 | GQ481352 |
| *Anthus pratensis* | UWBM 56714 | KBPBU586-06 | GQ481353 |
| *Anthus pratensis* | UWBM 49677 | KBPBU585-06 | GQ481354 |
| *Anthus richardi* | UWBM 59819 | KBPBU589-06 | GQ481355 |
| *Anthus richardi* | UWBM 57822 | KBPBU593-06 | GQ481356 |
| *Anthus rubescens* | UWBM 47533 | KBPBU602-06 | GQ481357 |
| *Anthus rubescens* | UWBM 44206 | KBPBU598-06 | GQ481358 |
| *Anthus rubescens* | UWBM 44079 | KBPBU595-06 | GQ481359 |
| *Anthus rubescens* | UWBM 51828 | KBPBU594-06 | GQ481360 |
| *Anthus rubescens* | UWBM 46907 | KBPBU596-06 | GQ481361 |
| *Anthus rubescens* | UWBM 51550 | KBPBU597-06 | GQ481362 |
| *Anthus spinoletta* | UWBM 67639 | KBPBU603-06 | GQ481363 |
| *Anthus spinoletta* | UWBM 46523 | KBPBU599-06 | GQ481364 |
| *Anthus spinoletta* | UWBM 57991 | KBPBU600-06 | GQ481365 |
| *Anthus spinoletta* | UWBM 51827 | KBPBU601-06 | GQ481366 |
| *Anthus trivialis* | UWBM 46527 | KBPBU604-06 | GQ481367 |
| *Anthus trivialis* | UWBM 57886 | KBPBU605-06 | GQ481368 |
| *Anthus trivialis* | UWBM 49488 | KBPBU607-06 | GQ481369 |
| *Anthus trivialis* | UWBM 51790 | KBPBU606-06 | GQ481370 |
| *Apus pacificus* | UWBM 46961 | KBPBU427-06 | GQ481371 |
| *Ardea cinerea* | UWBM 47173 | KBPBU013-06 | GQ481372 |
| *Ardeola bacchus* | ZMMU EAK 117 | KBPZM082-07 | GQ481373 |
| *Ardeola ralloides* | UWBM 61365 | KBPBU014-06 | GQ481374 |
| *Arenaria interpres* | UWBM 51166 | KBPBU196-06 | GQ481375 |
| *Arenaria interpres* | UWBM 44093 | KBPBU195-06 | GQ481376 |
| *Arenaria interpres* | UWBM 44328 | KBPBU197-06 | GQ481377 |
| *Arenaria interpres* | UWBM 43962 | KBPBU194-06 | GQ481378 |
| *Asio flammeus* | ZMMU RYA 1901 | KBPZM231-07 | GQ481379 |
| *Asio flammeus* | UWBM 47187 | KBPBU398-06 | GQ481380 |
| *Asio flammeus* | UWBM 51664 | KBPBU399-06 | GQ481381 |
| *Asio otus* | UWBM 59694 | KBPBU403-06 | GQ481382 |
| *Asio otus* | UWBM 66592 | KBPBU402-06 | GQ481383 |
| *Asio otus* | UWBM 67755 | KBPBU401-06 | GQ481384 |
| *Asio otus* | UWBM 57801 | KBPBU400-06 | GQ481385 |
| *Athene noctua* | UWBM 66569 | KBPBU405-06 | GQ481386 |
| *Aythya marila* | UWBM 59568 | KBPBU048-06 | GQ481387 |
| *Aythya nyroca* | UWBM 46236 | KBPBU049-06 | GQ481388 |
| *Bombycilla garrulus* | UWBM 56928 | KBPBU630-06 | GQ481389 |
| *Bombycilla garrulus* | UWBM 56735 | KBPBU629-06 | GQ481390 |
| *Bombycilla garrulus* | UWBM 49779 | KBPBU631-06 | GQ481391 |
| *Bombycilla garrulus* | UWBM 49780 | KBPBU632-06 | GQ481392 |
| *Bombycilla garrulus* | UWBM 52673 | KBPBU628-06 | GQ481393 |
| *Bonasa bonasia* | UWBM 51765 | KBPBU111-06 | GQ481394 |
| *Bonasa bonasia* | UWBM 52631 | KBPBU112-06 | GQ481395 |
| *Bonasa bonasia* | UWBM 60114 | KBPBU113-06 | GQ481396 |
| *Bonasa bonasia* | UWBM 47407 | KBPBU109-06 | GQ481397 |
| *Bonasa bonasia* | UWBM 51152 | KBPBU110-06 | GQ481398 |
| *Brachyramphus perdix* | UWBM 44434 | KBPBU357-06 | GQ481399 |
| *Brachyramphus perdix* | UWBM 44405 | KBPBU356-06 | GQ481400 |
| *Bradypterus tacsanowskius* | UWBM 51699 | KBPBR422-07 | GQ481401 |
| *Bradypterus tacsanowskius* | UWBM 58063 | KBPBR222-07 | GQ481402 |
| *Bradypterus tacsanowskius* | UWBM 59993 | KBPBR192-07 | GQ481403 |
| *Bucephala clangula* | UWBM 56566 | KBPBU050-06 | GQ481404 |
| *Burhinus oedicnemus* | UWBM 56911 | KBPBU134-06 | GQ481405 |
| *Buteo buteo* | UWBM 57802 | KBPBU064-06 | GQ481406 |
| *Buteo buteo* | UWBM 57797 | KBPBU063-06 | GQ481407 |
| *Buteo buteo* | ZMMU 31a | KBPZM019-06 | GQ481408 |
| *Butorides striata* | UWBM 75428 | KBPBU755-06 | GQ481409 |
| *Butorides striata* | UWBM 75441 | KBPBU757-06 | GQ481410 |
| *Butorides striata* | UWBM 75440 | KBPBU756-06 | GQ481411 |
| *Calandrella acutirostris* | UWBM 46245 | KBPBU519-06 | GQ481412 |
| *Calandrella brachydactyla* | UWBM 64797 | KBPBU527-06 | GQ481413 |
| *Calandrella cheleensis* | UWBM 59838 | KBPBU520-06 | GQ481414 |
| *Calandrella rufescens* | UWBM 75928 | KBPBU518-06 | GQ481415 |
| *Calandrella rufescens* | UWBM 60043 | KBPBU525-06 | GQ481416 |
| *Calandrella rufescens* | UWBM 67676 | KBPBU516-06 | GQ481417 |
| *Calandrella rufescens* | UWBM 75927 | KBPBU517-06 | GQ481418 |
| *Calandrella rufescens* | UWBM 66590 | KBPBU522-06 | GQ481419 |
| *Calandrella rufescens* | UWBM 60033 | KBPBU526-06 | GQ481420 |
| *Calandrella rufescens* | UWBM 59938 | KBPBU524-06 | GQ481421 |
| *Calandrella rufescens* | UWBM 57828 | KBPBU528-06 | GQ481422 |
| *Calcarius lapponicus* | UWBM 43906 | KBPBK070-08 | GQ481423 |
| *Calcarius lapponicus* | UWBM 49663 | KBPBK033-08 | GQ481424 |
| *Calcarius lapponicus* | UWBM 44321 | KBPBK175-08 | GQ481425 |
| *Calcarius lapponicus* | UWBM 44514 | KBPBK254-08 | GQ481426 |
| *Calcarius lapponicus* | UWBM 51685 | KBPBK281-08 | GQ481427 |
| *Calcarius lapponicus* | UWBM 59410 | KBPBK212-08 | GQ481428 |
| *Calidris alba* | UWBM 44650 | KBPBU214-06 | GQ481429 |
| *Calidris alba* | UWBM 61348 | KBPBU217-06 | GQ481430 |
| *Calidris alba* | UWBM 59596 | KBPBU215-06 | GQ481431 |
| *Calidris alba* | UWBM 61347 | KBPBU216-06 | GQ481432 |
| *Calidris alpina* | UWBM 49659 | KBPBU203-06 | GQ481433 |
| *Calidris alpina* | UWBM 51106 | KBPBU204-06 | GQ481434 |
| *Calidris canutus* | UWBM 44173 | KBPBU220-06 | GQ481435 |
| *Calidris canutus* | UWBM 43967 | KBPBU218-06 | GQ481436 |
| *Calidris canutus* | UWBM 43968 | KBPBU219-06 | GQ481437 |
| *Calidris ferruginea* | UWBM 73192 | KBPBU781-06 | GQ481438 |
| *Calidris minuta* | UWBM 59575 | KBPBU206-06 | GQ481439 |
| *Calidris subminuta* | UWBM 51103 | KBPBU209-06 | GQ481440 |
| *Calidris temminckii* | UWBM 49687 | KBPBU211-06 | GQ481441 |
| *Calidris tenuirostris* | UWBM 44469 | KBPBU223-06 | GQ481442 |
| *Calidris tenuirostris* | UWBM 44219 | KBPBU222-06 | GQ481443 |
| *Calidris tenuirostris* | UWBM 43884 | KBPBU221-06 | GQ481444 |
| *Caprimulgus europaeus* | UWBM 51766 | KBPBU414-06 | GQ481445 |
| *Caprimulgus europaeus* | UWBM 51775 | KBPBU415-06 | GQ481446 |
| *Caprimulgus europaeus* | UWBM 66730 | KBPBU413-06 | GQ481447 |
| *Caprimulgus europaeus* | UWBM 61355 | KBPBU412-06 | GQ481448 |
| *Caprimulgus indicus* | UWBM 47094 | KBPBU409-06 | GQ481449 |
| *Caprimulgus indicus* | UWBM 47117 | KBPBU410-06 | GQ481450 |
| *Carduelis cannabina* | UWBM 57390 | KBPBK352-08 | GQ481451 |
| *Carduelis cannabina* | UWBM 64643 | KBPBK312-08 | GQ481452 |
| *Carduelis cannabina* | UWBM 49372 | KBPBK018-08 | GQ481453 |
| *Carduelis cannabina* | UWBM 46520 | KBPBK147-08 | GQ481454 |
| *Carduelis cannabina* | UWBM 74306 | KBPBK199-08 | GQ481455 |
| *Carduelis carduelis* | UWBM 61402 | KBPBK303-08 | GQ481456 |
| *Carduelis carduelis* | UWBM 49329 | KBPBK016-08 | GQ481457 |
| *Carduelis carduelis* | UWBM 49589 | KBPBK030-08 | GQ481458 |
| *Carduelis carduelis* | UWBM 60994 | KBPBK157-08 | GQ481459 |
| *Carduelis carduelis* | UWBM 49521 | KBPBK026-08 | GQ481460 |
| *Carduelis carduelis* | UWBM 67559 | KBPBK180-08 | GQ481461 |
| *Carduelis chloris* | UWBM 49581 | KBPBK029-08 | GQ481462 |
| *Carduelis chloris* | UWBM 49325 | KBPBK013-08 | GQ481463 |
| *Carduelis chloris* | UWBM 74318 | KBPBK206-08 | GQ481464 |
| *Carduelis chloris* | UWBM 61434 | KBPBK305-08 | GQ481465 |
| *Carduelis flammea* | UWBM 74305 | KBPBK198-08 | GQ481466 |
| *Carduelis flammea* | UWBM 43933 | KBPBK071-08 | GQ481467 |
| *Carduelis flammea* | UWBM 49661 | KBPBK032-08 | GQ481468 |
| *Carduelis flammea* | UWBM 82171 | KBPBK169-08 | GQ481469 |
| *Carduelis flammea* | UWBM 52605 | KBPBK272-08 | GQ481470 |
| *Carduelis flammea* | UWBM 59576 | KBPBK297-08 | GQ481471 |
| *Carduelis flammea* | UWBM 82288 | KBPBK335-08 | GQ481472 |
| *Carduelis flammea* | UWBM 51893 | KBPBK290-08 | GQ481473 |
| *Carduelis flammea* | UWBM 56958 | KBPBK294-08 | GQ481474 |
| *Carduelis flammea* | UWBM 73128 | KBPBK004-08 | GQ481475 |
| *Carduelis flavirostris* | UWBM 66340 | KBPBK208-08 | GQ481476 |
| *Carduelis flavirostris* | UWBM 66632 | KBPBK317-08 | GQ481477 |
| *Carduelis flavirostris* | UWBM 66350 | KBPBK209-08 | GQ481478 |
| *Carduelis flavirostris* | UWBM 66571 | KBPBK223-08 | GQ481479 |
| *Carduelis hornemanni* | UWBM 44485 | KBPBK253-08 | GQ481480 |
| *Carduelis sinica* | UWBM 47484 | KBPBK341-08 | GQ481481 |
| *Carduelis sinica* | UWBM 44022 | KBPBK076-08 | GQ481482 |
| *Carduelis sinica* | UWBM 74952 | KBPBK238-08 | GQ481483 |
| *Carduelis sinica* | UWBM 60123 | KBPBK130-08 | GQ481484 |
| *Carduelis sinica* | ZMMU RYA 1799 | KBPZM215-07 | GQ481485 |
| *Carduelis sinica* | ZMMU RYA 1801 | KBPZM216-07 | GQ481486 |
| *Carduelis sinica* | ZMMU RYA 1725 | KBPZM186-07 | GQ481487 |
| *Carduelis sinica* | ZMMU RYA 1633 | KBPZM137-07 | GQ481488 |
| *Carduelis sinica* | ZMMU RYA 1620 | KBPZM135-07 | GQ481489 |
| *Carduelis spinus* | UWBM 74976 | KBPBK239-08 | GQ481490 |
| *Carduelis spinus* | ZMMU RYA 1744 | KBPZM193-07 | GQ481491 |
| *Carduelis spinus* | ZMMU RYA 1571 | KBPZM120-07 | GQ481492 |
| *Carduelis spinus* | ZMMU RYA 1735 | KBPZM189-07 | GQ481493 |
| *Carduelis spinus* | UWBM 59685 | KBPBK058-08 | GQ481494 |
| *Carduelis spinus* | UWBM 44001 | KBPBK074-08 | GQ481495 |
| *Carduelis spinus* | UWBM 49490 | KBPBK023-08 | GQ481496 |
| *Carduelis spinus* | UWBM 49740 | KBPBK036-08 | GQ481497 |
| *Carduelis spinus* | UWBM 73107 | KBPBK002-08 | GQ481498 |
| *Carduelis spinus* | UWBM 61520 | KBPBK310-08 | GQ481499 |
| *Carduelis spinus* | UWBM 47508 | KBPBK342-08 | GQ481500 |
| *Carduelis spinus* | UWBM 82185 | KBPBK171-08 | GQ481501 |
| *Carduelis spinus* | UWBM 47133 | KBPBK260-08 | GQ481502 |
| *Carduelis spinus* | ZMMU RYA 1758 | KBPZM196-07 | GQ481503 |
| *Carduelis spinus* | ZMMU RYA 1812 | KBPZM219-07 | GQ481504 |
| *Carpodacus erythrinus* | UWBM 51703 | KBPBK282-08 | GQ481505 |
| *Carpodacus erythrinus* | UWBM 46369 | KBPBK109-08 | GQ481506 |
| *Carpodacus erythrinus* | UWBM 60115 | KBPBK129-08 | GQ481507 |
| *Carpodacus erythrinus* | UWBM 49436 | KBPBK020-08 | GQ481508 |
| *Carpodacus erythrinus* | UWBM 51563 | KBPBK277-08 | GQ481509 |
| *Carpodacus erythrinus* | UWBM 46298 | KBPBK068-08 | GQ481510 |
| *Carpodacus erythrinus* | UWBM 71722 | KBPBK140-08 | GQ481511 |
| *Carpodacus erythrinus* | UWBM 57894 | KBPBK052-08 | GQ481512 |
| *Carpodacus erythrinus* | UWBM 78449 | KBPBK236-08 | GQ481513 |
| *Carpodacus erythrinus* | UWBM 64699 | KBPBK313-08 | GQ481514 |
| *Carpodacus erythrinus* | UWBM 82295 | KBPBK336-08 | GQ481515 |
| *Carpodacus erythrinus* | UWBM 60009 | KBPBK106-08 | GQ481516 |
| *Carpodacus erythrinus* | UWBM 74301 | KBPBK202-08 | GQ481517 |
| *Carpodacus erythrinus* | UWBM 66445 | KBPBK215-08 | GQ481518 |
| *Carpodacus erythrinus* | UWBM 73101 | KKBNA420-05 | GQ481519 |
| *Carpodacus erythrinus* | UWBM 49445 | KKBNA310-05 | GQ481520 |
| *Carpodacus erythrinus* | UWBM 47013 | KKBNA288-05 | GQ481521 |
| *Carpodacus erythrinus* | UWBM 44603 | KBPBK257-08 | GQ481522 |
| *Carpodacus roseus* | UWBM 57890 | KBPBK051-08 | GQ481523 |
| *Carpodacus roseus* | UWBM 74835 | KBPBK356-08 | GQ481524 |
| *Carpodacus roseus* | UWBM 60180 | KBPBK132-08 | GQ481525 |
| *Carpodacus roseus* | UWBM 52655 | KBPBK275-08 | GQ481526 |
| *Carpodacus roseus* | UWBM 52654 | KBPBK274-08 | GQ481527 |
| *Carpodacus rubicilla* | UWBM 66530 | KBPBK220-08 | GQ481528 |
| *Carpodacus rubicilla* | UWBM 76129 | KBPBK221-08 | GQ481529 |
| *Cecropis daurica* | UWBM 47217 | KBPBU540-06 | GQ481530 |
| *Cecropis daurica* | UWBM 75470 | KBPBU768-06 | GQ481531 |
| *Cecropis daurica* | UWBM 75469 | KBPBU767-06 | GQ481532 |
| *Cecropis daurica* | UWBM 46926 | KBPBU539-06 | GQ481533 |
| *Cepphus carbo* | UWBM 44430 | KBPBU354-06 | GQ481534 |
| *Cepphus carbo* | UWBM 44404 | KBPBU352-06 | GQ481535 |
| *Cepphus carbo* | UWBM 44429 | KBPBU353-06 | GQ481536 |
| *Certhia brachydactyla* | UWBM 61534 | KBPBR460-07 | GQ481537 |
| *Certhia familiaris* | ZMMU RYA 1601 | KBPZM131-07 | GQ481538 |
| *Certhia familiaris* | ZMMU RYA 1787 | KBPZM207-07 | GQ481539 |
| *Certhia familiaris* | ZMMU RYA 1808 | KBPZM217-07 | GQ481540 |
| *Certhia familiaris* | ZMMU RYA 1602 | KBPZM132-07 | GQ481541 |
| *Certhia familiaris* | UWBM 49598 | KBPBR119-07 | GQ481542 |
| *Certhia familiaris* | UWBM 61095 | KBPBR271-07 | GQ481543 |
| *Certhia familiaris* | UWBM 72210 | KBPBR491-07 | GQ481544 |
| *Certhia familiaris* | UWBM 47062 | KBPBR163-07 | GQ481545 |
| *Certhia familiaris* | UWBM 51750 | KBPBR423-07 | GQ481546 |
| *Cettia cetti* | UWBM 56601 | KBPBR173-07 | GQ481547 |
| *Cettia diphone* | ZMMU RYA 1737 | KBPZM190-07 | GQ481548 |
| *Cettia diphone* | UWBM 83173 | KBPBR376-07 | GQ481549 |
| *Cettia diphone* | ZMMU EAK 101 | KBPZM074-07 | GQ481550 |
| *Cettia diphone* | ZMMU RYA 1097 | KBPZM094-07 | GQ481551 |
| *Cettia diphone* | ZMMU RYA 1161 | KBPZM096-07 | GQ481552 |
| *Cettia diphone* | ZMMU RYA 1688 | KBPZM160-07 | GQ481553 |
| *Cettia diphone* | ZMMU RYA 1726 | KBPZM187-07 | GQ481554 |
| *Cettia diphone* | ZMMU RYA 1176 | KBPZM097-07 | GQ481555 |
| *Cettia diphone* | ZMMU RYA 1200 | KBPZM101-07 | GQ481556 |
| *Cettia diphone* | UWBM 74811 | KBPBR405-07 | GQ481557 |
| *Cettia diphone* | UWBM 47550 | KBPBR514-07 | GQ481558 |
| *Cettia diphone* | UWBM 75317 | KBPBR367-07 | GQ481559 |
| *Charadrius alexandrinus* | UWBM 60141 | KBPBU176-06 | GQ481560 |
| *Charadrius alexandrinus* | UWBM 60140 | KBPBU175-06 | GQ481561 |
| *Charadrius alexandrinus* | UWBM 59943 | KBPBU173-06 | GQ481562 |
| *Charadrius alexandrinus* | UWBM 60131 | KBPBU174-06 | GQ481563 |
| *Charadrius dubius* | UWBM 47418 | KBPBU181-06 | GQ481564 |
| *Charadrius dubius* | UWBM 66650 | KBPBU180-06 | GQ481565 |
| *Charadrius dubius* | UWBM 61031 | KBPBU179-06 | GQ481566 |
| *Charadrius dubius* | UWBM 46242 | KBPBU178-06 | GQ481567 |
| *Charadrius dubius* | UWBM 59714 | KBPBU177-06 | GQ481568 |
| *Charadrius leschenaultii* | UWBM 66641 | KBPBU186-06 | GQ481569 |
| *Charadrius mongolus* | UWBM 44445 | KBPBU185-06 | GQ481570 |
| *Charadrius mongolus* | UWBM 51110 | KBPBU184-06 | GQ481571 |
| *Charadrius mongolus* | UWBM 51109 | KBPBU183-06 | GQ481572 |
| *Charadrius morinellus* | UWBM 66722 | KBPBU182-06 | GQ481573 |
| *Chlidonias hybrida* | UWBM 56920 | KBPBU334-06 | GQ481574 |
| *Chlidonias hybrida* | UWBM 59947 | KBPBU337-06 | GQ481575 |
| *Chlidonias hybrida* | UWBM 61480 | KBPBU335-06 | GQ481576 |
| *Chlidonias hybrida* | UWBM 59704 | KBPBU336-06 | GQ481577 |
| *Chlidonias leucopterus* | UWBM 60147 | KBPBU332-06 | GQ481578 |
| *Chlidonias leucopterus* | UWBM 61238 | KBPBU331-06 | GQ481579 |
| *Chlidonias leucopterus* | UWBM 61237 | KBPBU330-06 | GQ481580 |
| *Chlidonias niger* | UWBM 61479 | KBPBU329-06 | GQ481581 |
| *Chlidonias niger* | UWBM 56906 | KBPBU328-06 | GQ481582 |
| *Chlidonias niger* | UWBM 56893 | KBPBU326-06 | GQ481583 |
| *Cinclus cinclus* | UWBM 61537 | KBPBU634-06 | GQ481584 |
| *Cinclus cinclus* | UWBM 61539 | KBPBU635-06 | GQ481585 |
| *Cinclus cinclus* | UWBM 64772 | KBPBU637-06 | GQ481586 |
| *Cinclus cinclus* | UWBM 64752 | KBPBU636-06 | GQ481587 |
| *Cinclus cinclus* | UWBM 46430 | KBPBU633-06 | GQ481588 |
| *Cinclus pallasii* | ZMMU EAK 097 | KBPZM073-07 | GQ481589 |
| *Cinclus pallasii* | UWBM 51144 | KBPBU638-06 | GQ481590 |
| *Circus aeruginosus* | UWBM 56488 | KBPBU075-06 | GQ481591 |
| *Circus aeruginosus* | UWBM 56450 | KBPBU073-06 | GQ481592 |
| *Clangula hyemalis* | UWBM 59523 | KBPBU053-06 | GQ481593 |
| *Clangula hyemalis* | UWBM 43894 | KBPBU051-06 | GQ481594 |
| *Clangula hyemalis* | UWBM 43917 | KBPBU052-06 | GQ481595 |
| *Coccothraustes coccothraustes* | UWBM 60125 | KBPBK131-08 | GQ481596 |
| *Coccothraustes coccothraustes* | UWBM 49326 | KBPBK014-08 | GQ481597 |
| *Coccothraustes coccothraustes* | UWBM 75179 | KBPBK243-08 | GQ481598 |
| *Coccothraustes coccothraustes* | UWBM 47236 | KBPBK263-08 | GQ481599 |
| *Coccothraustes coccothraustes* | UWBM 83260 | KBPBK250-08 | GQ481600 |
| *Coccothraustes coccothraustes* | UWBM 74326 | KBPBK194-08 | GQ481601 |
| *Coccothraustes coccothraustes* | UWBM 58089 | KBPBK124-08 | GQ481602 |
| *Coccothraustes personatus* | UWBM 75152 | KBPBK240-08 | GQ481603 |
| *Coccothraustes personatus* | UWBM 75166 | KBPBK242-08 | GQ481604 |
| *Columba livia* | UWBM 66460 | KBPBU370-06 | GQ481605 |
| *Columba livia* | UWBM 46303 | KBPBU369-06 | GQ481606 |
| *Columba palumbus* | UWBM 56505 | KBPBU372-06 | GQ481607 |
| *Columba palumbus* | UWBM 64851 | KBPBU374-06 | GQ481608 |
| *Columba palumbus* | UWBM 56694 | KBPBU373-06 | GQ481609 |
| *Columba rupestris* | UWBM 66642 | KBPBU371-06 | GQ481610 |
| *Columba rupestris* | UWBM 59987 | KBPBU364-06 | GQ481611 |
| *Columba rupestris* | UWBM 66643 | KBPBU367-06 | GQ481612 |
| *Columba rupestris* | UWBM 59803 | KBPBU366-06 | GQ481613 |
| *Columba rupestris* | UWBM 66630 | KBPBU368-06 | GQ481614 |
| *Columba rupestris* | UWBM 59762 | KBPBU365-06 | GQ481615 |
| *Coracias garrulus* | UWBM 56660 | KBPBU434-06 | GQ481616 |
| *Coracias garrulus* | UWBM 56480 | KBPBU433-06 | GQ481617 |
| *Coracias garrulus* | UWBM 46244 | KBPBU432-06 | GQ481618 |
| *Coracias garrulus* | UWBM 46243 | KBPBU431-06 | GQ481619 |
| *Corvus corax* | UWBM 56544 | KBPBK043-08 | GQ481620 |
| *Corvus corax* | UWBM 57899 | KBPBK053-08 | GQ481621 |
| *Corvus corax* | UWBM 46861 | KBPBK006-08 | GQ481622 |
| *Corvus corax* | UWBM 61493 | KBPBK308-08 | GQ481623 |
| *Corvus corax* | UWBM 64779 | KBPBK315-08 | GQ481624 |
| *Corvus corone* | UWBM 66593 | KBPBK226-08 | GQ481625 |
| *Corvus corone* | UWBM 56593 | KBPBK085-08 | GQ481626 |
| *Corvus corone* | UWBM 44426 | KBPBK251-08 | GQ481627 |
| *Corvus corone* | UWBM 69716 | KBPBK017-08 | GQ481628 |
| *Corvus corone* | UWBM 58036 | KBPBK118-08 | GQ481629 |
| *Corvus corone* | UWBM 47138 | KBPBK261-08 | GQ481630 |
| *Corvus corone* | UWBM 51890 | KBPBK289-08 | GQ481631 |
| *Corvus corone* | UWBM 73347 | KBPBK179-08 | GQ481632 |
| *Corvus dauuricus* | UWBM 57908 | KBPBK054-08 | GQ481633 |
| *Corvus dauuricus* | UWBM 74563 | KBPBK141-08 | GQ481634 |
| *Corvus dauuricus* | UWBM 75394 | KBPBK244-08 | GQ481635 |
| *Corvus dauuricus* | UWBM 58041 | KBPBK119-08 | GQ481636 |
| *Corvus frugilegus* | UWBM 46364 | KBPBK108-08 | GQ481637 |
| *Corvus frugilegus* | UWBM 74565 | KBPBK142-08 | GQ481638 |
| *Corvus frugilegus* | UWBM 49247 | KBPBK011-08 | GQ481639 |
| *Corvus frugilegus* | UWBM 59801 | KBPBK096-08 | GQ481640 |
| *Corvus macrorhynchos* | UWBM 47041 | KBPBK079-08 | GQ481641 |
| *Corvus macrorhynchos* | UWBM 72006 | KBPBK178-08 | GQ481642 |
| *Corvus macrorhynchos* | UWBM 83129 | KBPBK249-08 | GQ481643 |
| *Corvus monedula* | UWBM 46239 | KBPBK063-08 | GQ481644 |
| *Corvus monedula* | UWBM 56496 | KBPBK039-08 | GQ481645 |
| *Corvus monedula* | UWBM 72062 | KBPBK322-08 | GQ481646 |
| *Corvus monedula* | UWBM 71351 | KBPBK351-08 | GQ481647 |
| *Coturnix coturnix* | UWBM 61369 | KBPBU120-06 | GQ481648 |
| *Coturnix coturnix* | UWBM 61354 | KBPBU118-06 | GQ481649 |
| *Coturnix japonica* | UWBM 59837 | KBPBU116-06 | GQ481650 |
| *Coturnix japonica* | UWBM 59896 | KBPBU117-06 | GQ481651 |
| *Coturnix japonica* | UWBM 73309 | KBPBU776-06 | GQ481652 |
| *Coturnix japonica* | UWBM 73631 | KBPBU778-06 | GQ481653 |
| *Crex crex* | UWBM 49624 | KBPBU131-06 | GQ481654 |
| *Cuculus canorus* | UWBM 56594 | KBPBU384-06 | GQ481655 |
| *Cuculus canorus* | UWBM 47024 | KBPBU385-06 | GQ481656 |
| *Cuculus canorus* | UWBM 66441 | KBPBU386-06 | GQ481657 |
| *Cuculus canorus* | UWBM 58010 | KBPBU383-06 | GQ481658 |
| *Cuculus canorus* | UWBM 46365 | KBPBU382-06 | GQ481659 |
| *Cuculus optatus* | UWBM 44563 | KBPBU387-06 | GQ481660 |
| *Cuculus optatus* | UWBM 44401 | KBPBU388-06 | GQ481661 |
| *Cuculus optatus* | UWBM 56743 | KBPBU391-06 | GQ481662 |
| *Cuculus optatus* | UWBM 71703 | KBPBU389-06 | GQ481663 |
| *Cuculus optatus* | UWBM 49457 | KBPBU390-06 | GQ481664 |
| *Cyanistes caeruleus* | ZMMU 113a | KBPZM049-06 | GQ481665 |
| *Cyanistes caeruleus* | ZMMU 114a | KBPZM050-06 | GQ481666 |
| *Cyanistes caeruleus* | UWBM 49322 | KBPBR086-07 | GQ481667 |
| *Cyanistes caeruleus* | ZMMU RYA 1462 | KBPZM115-07 | GQ481668 |
| *Cyanistes caeruleus* | ZMMU MVK 477 | KBPZM084-07 | GQ481669 |
| *Cyanistes caeruleus* | ZMMU MVK 485 | KBPZM086-07 | GQ481670 |
| *Cyanistes caeruleus* | ZMMU MVK 483 | KBPZM085-07 | GQ481671 |
| *Cyanistes caeruleus* | ZMMU 52a | KBPZM023-06 | GQ481672 |
| *Cyanistes caeruleus* | ZMMU 53a | KBPZM024-06 | GQ481673 |
| *Cyanistes caeruleus* | UWBM 82161 | KBPBR283-07 | GQ481674 |
| *Cyanistes caeruleus* | UWBM 74196 | KBPBR318-07 | GQ481675 |
| *Cyanistes caeruleus* | UWBM 56695 | KBPBR178-07 | GQ481676 |
| *Cyanistes caeruleus* | UWBM 61420 | KBPBR451-07 | GQ481677 |
| *Cyanistes cyanus* | ZMMU 50\2000 | KBPZM064-07 | GQ481678 |
| *Cyanistes cyanus* | UWBM 59700 | KBPBR148-07 | GQ481679 |
| *Cyanistes cyanus* | ZMMU RYA 338 | KBPZM249-07 | GQ481680 |
| *Cyanistes cyanus* | ZMMU RYA 301 | KBPZM247-07 | GQ481681 |
| *Cyanistes cyanus* | ZMMU 51\2000 | KBPZM065-07 | GQ481682 |
| *Cyanistes cyanus* | UWBM 67561 | KBPBR292-07 | GQ481683 |
| *Cyanopica cyanus* | UWBM 78227 | KBPBK133-08 | GQ481684 |
| *Cyanopica cyanus* | UWBM 74588 | KBPBK152-08 | GQ481685 |
| *Cyanopica cyanus* | UWBM 71977 | KBPBK177-08 | GQ481686 |
| *Cyanopica cyanus* | UWBM 60095 | KBPBK128-08 | GQ481687 |
| *Cyanopica cyanus* | UWBM 46448 | KBPBK112-08 | GQ481688 |
| *Delichon dasypus* | UWBM 51835 | KBPBU551-06 | GQ481689 |
| *Delichon dasypus* | UWBM 46947 | KBPBU553-06 | GQ481690 |
| *Delichon dasypus* | UWBM 51837 | KBPBU552-06 | GQ481691 |
| *Delichon dasypus* | UWBM 46968 | KBPBU554-06 | GQ481692 |
| *Delichon urbicum* | UWBM 67727 | KBPBU549-06 | GQ481693 |
| *Delichon urbicum* | UWBM 49186 | KBPBU548-06 | GQ481694 |
| *Delichon urbicum* | UWBM 66624 | KBPBU550-06 | GQ481695 |
| *Dendrocopos canicapillus* | UWBM 72096 | KBPBU467-06 | GQ481696 |
| *Dendrocopos canicapillus* | UWBM 51079 | KBPBU466-06 | GQ481697 |
| *Dendrocopos kizuki* | UWBM 47379 | KBPBU472-06 | GQ481698 |
| *Dendrocopos kizuki* | UWBM 51183 | KBPBU468-06 | GQ481699 |
| *Dendrocopos kizuki* | UWBM 72254 | KBPBU470-06 | GQ481700 |
| *Dendrocopos kizuki* | UWBM 47374 | KBPBU471-06 | GQ481701 |
| *Dendrocopos kizuki* | UWBM 71712 | KBPBU469-06 | GQ481702 |
| *Dendrocopos leucotos* | UWBM 46354 | KBPBU473-06 | GQ481703 |
| *Dendrocopos leucotos* | UWBM 49418 | KBPBU475-06 | GQ481704 |
| *Dendrocopos leucotos* | UWBM 57279 | KBPBU476-06 | GQ481705 |
| *Dendrocopos leucotos* | UWBM 47343 | KBPBU474-06 | GQ481706 |
| *Dendrocopos leucotos* | UWBM 72175 | KBPBU477-06 | GQ481707 |
| *Dendrocopos major* | UWBM 46935 | KBPBU478-06 | GQ481708 |
| *Dendrocopos major* | UWBM 51700 | KBPBU481-06 | GQ481709 |
| *Dendrocopos major* | UWBM 49397 | KBPBU479-06 | GQ481710 |
| *Dendrocopos major* | UWBM 51632 | KBPBU480-06 | GQ481711 |
| *Dendrocopos major* | UWBM 56745 | KBPBU482-06 | GQ481712 |
| *Dendrocopos medius* | UWBM 64847 | KBPBU485-06 | GQ481713 |
| *Dendrocopos medius* | UWBM 61173 | KBPBU483-06 | GQ481714 |
| *Dendrocopos medius* | UWBM 61425 | KBPBU484-06 | GQ481715 |
| *Dendrocopos minor* | UWBM 61410 | KBPBU486-06 | GQ481716 |
| *Dendrocopos minor* | UWBM 72267 | KBPBU488-06 | GQ481717 |
| *Dendrocopos minor* | UWBM 52617 | KBPBU487-06 | GQ481718 |
| *Dendrocopos minor* | UWBM 75910 | KBPBU489-06 | GQ481719 |
| *Dendrocopos minor* | UWBM 49565 | KBPBU490-06 | GQ481720 |
| *Dryocopus martius* | UWBM 56955 | KBPBU452-06 | GQ481721 |
| *Dryocopus martius* | UWBM 64839 | KBPBU449-06 | GQ481722 |
| *Dryocopus martius* | UWBM 52589 | KBPBU450-06 | GQ481723 |
| *Dryocopus martius* | UWBM 47530 | KBPBU448-06 | GQ481724 |
| *Dryocopus martius* | UWBM 46980 | KBPBU451-06 | GQ481725 |
| *Egretta eulophotes* | ZMMU EAK 045 | KBPZM071-07 | GQ481726 |
| *Emberiza aureola* | UWBM 52534 | KBPBR412-07 | GQ481727 |
| *Emberiza aureola* | UWBM 82227 | KBPBR495-07 | GQ481728 |
| *Emberiza aureola* | UWBM 82287 | KBPBR499-07 | GQ481729 |
| *Emberiza aureola* | UWBM 59928 | KBPBR186-07 | GQ481730 |
| *Emberiza aureola* | UWBM 73118 | KBPBR071-07 | GQ481731 |
| *Emberiza aureola* | UWBM 78283 | KBPBR235-07 | GQ481732 |
| *Emberiza aureola* | UWBM 46588 | KBPBR259-07 | GQ481733 |
| *Emberiza aureola* | UWBM 58031 | KBPBR221-07 | GQ481734 |
| *Emberiza aureola* | UWBM 47574 | KBPBR518-07 | GQ481735 |
| *Emberiza aureola* | UWBM 74822 | KBPBR409-07 | GQ481736 |
| *Emberiza aureola* | UWBM 44597 | KBPBR384-07 | GQ481737 |
| *Emberiza bruniceps* | UWBM 46230 | KBPBR151-07 | GQ481738 |
| *Emberiza bruniceps* | UWBM 46486 | KBPBR244-07 | GQ481739 |
| *Emberiza bruniceps* | UWBM 46508 | KBPBR248-07 | GQ481740 |
| *Emberiza buchanani* | UWBM 57974 | KBPBR212-07 | GQ481741 |
| *Emberiza buchanani* | UWBM 46519 | KBPBR251-07 | GQ481742 |
| *Emberiza calandra* | UWBM 64676 | KBPBR464-07 | GQ481743 |
| *Emberiza calandra* | UWBM 61357 | KBPBR445-07 | GQ481744 |
| *Emberiza chrysophrys* | UWBM 73328 | KBPBR291-07 | GQ481745 |
| *Emberiza chrysophrys* | UWBM 73327 | KBPBR290-07 | GQ481746 |
| *Emberiza cia* | UWBM 46542 | KBPBR253-07 | GQ481747 |
| *Emberiza cia* | UWBM 46403 | KBPBR202-07 | GQ481748 |
| *Emberiza cioides* | UWBM 74812 | KBPBR406-07 | GQ481749 |
| *Emberiza cioides* | UWBM 57824 | KBPBR140-07 | GQ481750 |
| *Emberiza cioides* | UWBM 60154 | KBPBR228-07 | GQ481751 |
| *Emberiza cioides* | UWBM 67581 | KBPBR293-07 | GQ481752 |
| *Emberiza cioides* | UWBM 46405 | KBPBR203-07 | GQ481753 |
| *Emberiza citrinella* | ZMMU 25a | KBPZM013-06 | GQ481754 |
| *Emberiza citrinella* | UWBM 49556 | KBPBR116-07 | GQ481755 |
| *Emberiza citrinella* | UWBM 49313 | KBPBR084-07 | GQ481756 |
| *Emberiza citrinella* | UWBM 49587 | KBPBR118-07 | GQ481757 |
| *Emberiza citrinella* | UWBM 61126 | KBPBR274-07 | GQ481758 |
| *Emberiza elegans* | UWBM 59907 | KBPBR183-07 | GQ481759 |
| *Emberiza elegans* | UWBM 46867 | KBPBR073-07 | GQ481760 |
| *Emberiza fucata* | UWBM 47052 | KBPBR162-07 | GQ481761 |
| *Emberiza fucata* | UWBM 75462 | KBPBR370-07 | GQ481762 |
| *Emberiza fucata* | UWBM 59910 | KBPBR184-07 | GQ481763 |
| *Emberiza fucata* | UWBM 72100 | KBPBR483-07 | GQ481764 |
| *Emberiza godlewskii* | UWBM 51855 | KBPBR428-07 | GQ481765 |
| *Emberiza godlewskii* | UWBM 57948 | KBPBR209-07 | GQ481766 |
| *Emberiza hortulana* | UWBM 49363 | KBPBR091-07 | GQ481767 |
| *Emberiza leucocephalos* | UWBM 73419 | KBPBK228-08 | GQ481768 |
| *Emberiza leucocephalos* | UWBM 47310 | KBPBK265-08 | GQ481769 |
| *Emberiza leucocephalos* | UWBM 66576 | KBPBK225-08 | GQ481770 |
| *Emberiza leucocephalos* | UWBM 59990 | KBPBK104-08 | GQ481771 |
| *Emberiza leucocephalos* | UWBM 46456 | KBPBK113-08 | GQ481772 |
| *Emberiza melanocephala* | UWBM 56847 | KBPBK291-08 | GQ481773 |
| *Emberiza melanocephala* | UWBM 61566 | KBPBK214-08 | GQ481774 |
| *Emberiza pallasi* | UWBM 57830 | KBPBK045-08 | GQ481775 |
| *Emberiza pallasi* | UWBM 57996 | KBPBK117-08 | GQ481776 |
| *Emberiza pallasi* | UWBM 47188 | KBPBK262-08 | GQ481777 |
| *Emberiza pallasi* | UWBM 44263 | KBPBK174-08 | GQ481778 |
| *Emberiza pallasi* | UWBM 59816 | KBPBK097-08 | GQ481779 |
| *Emberiza pallasi* | UWBM 75802 | KBPBK190-08 | GQ481780 |
| *Emberiza pusilla* | UWBM 73472 | KBPBK231-08 | GQ481781 |
| *Emberiza pusilla* | UWBM 59579 | KBPBK299-08 | GQ481782 |
| *Emberiza pusilla* | UWBM 56517 | KBPBK041-08 | GQ481783 |
| *Emberiza pusilla* | UWBM 57820 | KBPBK044-08 | GQ481784 |
| *Emberiza pusilla* | UWBM 82286 | KBPBK334-08 | GQ481785 |
| *Emberiza pusilla* | UWBM 44241 | KBPBK173-08 | GQ481786 |
| *Emberiza pusilla* | UWBM 43939 | KBPBK072-08 | GQ481787 |
| *Emberiza pusilla* | UWBM 82166 | KBPBK167-08 | GQ481788 |
| *Emberiza pusilla* | UWBM 60048 | KBPBK126-08 | GQ481789 |
| *Emberiza pusilla* | UWBM 44472 | KBPBK252-08 | GQ481790 |
| *Emberiza rustica* | UWBM 49477 | KBPBK022-08 | GQ481791 |
| *Emberiza rustica* | UWBM 44027 | KBPBK077-08 | GQ481792 |
| *Emberiza rustica* | UWBM 78395 | KBPBK234-08 | GQ481793 |
| *Emberiza rustica* | UWBM 82313 | KBPBK337-08 | GQ481794 |
| *Emberiza rustica* | UWBM 56528 | KBPBK042-08 | GQ481795 |
| *Emberiza rustica* | UWBM 74648 | KBPBK267-08 | GQ481796 |
| *Emberiza rustica* | UWBM 51868 | KBPBK288-08 | GQ481797 |
| *Emberiza rustica* | UWBM 51597 | KBPBK278-08 | GQ481798 |
| *Emberiza rustica* | UWBM 78418 | KBPBK235-08 | GQ481799 |
| *Emberiza rutila* | UWBM 73120 | KBPBK003-08 | GQ481800 |
| *Emberiza rutila* | UWBM 57935 | KBPBK055-08 | GQ481801 |
| *Emberiza rutila* | UWBM 59697 | KBPBK059-08 | GQ481802 |
| *Emberiza rutila* | UWBM 72276 | KBPBK330-08 | GQ481803 |
| *Emberiza rutila* | UWBM 58394 | KBPBK353-08 | GQ481804 |
| *Emberiza rutila* | UWBM 73421 | KBPBK229-08 | GQ481805 |
| *Emberiza schoeniclus* | ZMMU RYA 1689 | KBPZM161-07 | GQ481806 |
| *Emberiza schoeniclus* | ZMMU RYA 1690 | KBPZM162-07 | GQ481807 |
| *Emberiza schoeniclus* | ZMMU RYA 1907 | KBPZM234-07 | GQ481808 |
| *Emberiza schoeniclus* | ZMMU RYA 1691 | KBPZM163-07 | GQ481809 |
| *Emberiza schoeniclus* | ZMMU RYA 1906 | KBPZM233-07 | GQ481810 |
| *Emberiza schoeniclus* | ZMMU 48a | KBPZM022-06 | GQ481811 |
| *Emberiza schoeniclus* | UWBM 73218 | KBPBK005-08 | GQ481812 |
| *Emberiza schoeniclus* | UWBM 59960 | KBPBK103-08 | GQ481813 |
| *Emberiza schoeniclus* | UWBM 49408 | KBPBK019-08 | GQ481814 |
| *Emberiza schoeniclus* | UWBM 49549 | KBPBK028-08 | GQ481815 |
| *Emberiza schoeniclus* | UWBM 83402 | KBPBK248-08 | GQ481816 |
| *Emberiza schoeniclus* | UWBM 82168 | KBPBK168-08 | GQ481817 |
| *Emberiza schoeniclus* | UWBM 72168 | KBPBK327-08 | GQ481818 |
| *Emberiza schoeniclus* | UWBM 59578 | KBPBK298-08 | GQ481819 |
| *Emberiza schoeniclus* | UWBM 67707 | KBPBK185-08 | GQ481820 |
| *Emberiza schoeniclus* | UWBM 74287 | KBPBK200-08 | GQ481821 |
| *Emberiza schoeniclus* | UWBM 59933 | KBPBK101-08 | GQ481822 |
| *Emberiza schoeniclus* | UWBM 61234 | KBPBK165-08 | GQ481823 |
| *Emberiza schoeniclus* | UWBM 44151 | KBPBK107-08 | GQ481824 |
| *Emberiza spodocephala* | ZMMU RYA 1782 | KBPZM205-07 | GQ481825 |
| *Emberiza spodocephala* | ZMMU RYA 1785 | KBPZM206-07 | GQ481826 |
| *Emberiza spodocephala* | ZMMU RYA 1810 | KBPZM218-07 | GQ481827 |
| *Emberiza spodocephala* | ZMMU RYA 1675 | KBPZM149-07 | GQ481828 |
| *Emberiza spodocephala* | ZMMU RYA 1712 | KBPZM176-07 | GQ481829 |
| *Emberiza spodocephala* | UWBM 59782 | KBPBK057-08 | GQ481830 |
| *Emberiza spodocephala* | UWBM 47066 | KBPBK080-08 | GQ481831 |
| *Emberiza spodocephala* | UWBM 74577 | KBPBK151-08 | GQ481832 |
| *Emberiza spodocephala* | UWBM 58431 | KBPBK354-08 | GQ481833 |
| *Emberiza spodocephala* | UWBM 83392 | KBPBK247-08 | GQ481834 |
| *Emberiza spodocephala* | UWBM 78207 | KBPBK136-08 | GQ481835 |
| *Emberiza spodocephala* | UWBM 73407 | KBPBK227-08 | GQ481836 |
| *Emberiza spodocephala* | UWBM 52613 | KBPBK273-08 | GQ481837 |
| *Emberiza spodocephala* | UWBM 78244 | KBPBK134-08 | GQ481838 |
| *Emberiza tristrami* | UWBM 74607 | KBPBK153-08 | GQ481839 |
| *Emberiza tristrami* | UWBM 71711 | KBPBK139-08 | GQ481840 |
| *Emberiza tristrami* | UWBM 47438 | KBPBK339-08 | GQ481841 |
| *Emberiza tristrami* | UWBM 74745 | KBPBK269-08 | GQ481842 |
| *Emberiza tristrami* | UWBM 72224 | KBPBK329-08 | GQ481843 |
| *Emberiza variabilis* | ZMMU EAK 102 | KBPZM075-07 | GQ481844 |
| *Emberiza variabilis* | ZMMU RYA 1813 | KBPZM220-07 | GQ481845 |
| *Emberiza variabilis* | UWBM 46944 | KBPBK008-08 | GQ481846 |
| *Emberiza variabilis* | UWBM 46950 | KBPBK009-08 | GQ481847 |
| *Emberiza yessoensis* | UWBM 72135 | KBPBK325-08 | GQ481848 |
| *Emberiza yessoensis* | UWBM 75466 | KBPBK246-08 | GQ481849 |
| *Emberiza yessoensis* | UWBM 72157 | KBPBK326-08 | GQ481850 |
| *Emberiza yessoensis* | UWBM 59941 | KBPBK102-08 | GQ481851 |
| *Eremophila alpestris* | UWBM 66333 | KBPBU537-06 | GQ481852 |
| *Eremophila alpestris* | UWBM 59836 | KBPBU535-06 | GQ481853 |
| *Eremophila alpestris* | UWBM 60039 | KBPBU536-06 | GQ481854 |
| *Erithacus akahige* | ZMMU RYA 1840 | KBPZM223-07 | GQ481855 |
| *Erithacus akahige* | ZMMU RYA 1585 | KBPZM123-07 | GQ481856 |
| *Erithacus akahige* | ZMMU RYA 1713 | KBPZM177-07 | GQ481857 |
| *Erithacus akahige* | ZMMU RYA 1746 | KBPZM195-07 | GQ481858 |
| *Erithacus akahige* | ZMMU RYA 1723 | KBPZM184-07 | GQ481859 |
| *Erithacus akahige* | UWBM 47481 | KBPBR065-06 | GQ481860 |
| *Erithacus akahige* | UWBM 47487 | KBPBR066-06 | GQ481861 |
| *Erithacus akahige* | UWBM 47490 | KBPBR067-06 | GQ481862 |
| *Erithacus rubecula* | ZMMU 14a | KBPZM002-06 | GQ481863 |
| *Erithacus rubecula* | UWBM 61398 | KBPBR063-06 | GQ481864 |
| *Erithacus rubecula* | UWBM 49456 | KBPBR013-06 | GQ481865 |
| *Erithacus rubecula* | UWBM 59664 | KBPBR061-06 | GQ481866 |
| *Erithacus rubecula* | UWBM 74231 | KBPBR046-06 | GQ481867 |
| *Erithacus rubecula* | UWBM 49765 | KBPBR019-06 | GQ481868 |
| *Erithacus rubecula* | UWBM 49285 | KBPBR006-06 | GQ481869 |
| *Eurystomus orientalis* | UWBM 72015 | KBPBU435-06 | GQ481870 |
| *Eurystomus orientalis* | UWBM 47198 | KBPBU436-06 | GQ481871 |
| *Falco amurensis* | UWBM 47521 | KBPBU076-06 | GQ481872 |
| *Falco amurensis* | UWBM 59901 | KBPBU077-06 | GQ481873 |
| *Falco columbarius* | UWBM 44262 | KBPBU079-06 | GQ481874 |
| *Falco naumanni* | UWBM 59764 | KBPBU080-06 | GQ481875 |
| *Falco naumanni* | UWBM 60035 | KBPBU081-06 | GQ481876 |
| *Falco subbuteo* | UWBM 51136 | KBPBU758-06 | GQ481877 |
| *Falco subbuteo* | UWBM 44306 | KBPBU759-06 | GQ481878 |
| *Falco subbuteo* | UWBM 57800 | KBPBU760-06 | GQ481879 |
| *Falco subbuteo* | UWBM 47520 | KBPBU761-06 | GQ481880 |
| *Falco tinnunculus* | UWBM 61104 | KBPBU082-06 | GQ481881 |
| *Falco tinnunculus* | UWBM 46363 | KBPBU083-06 | GQ481882 |
| *Falco tinnunculus* | UWBM 46489 | KBPBU084-06 | GQ481883 |
| *Ficedula albicilla* | UWBM 47371 | KBPBR417-07 | GQ481884 |
| *Ficedula albicilla* | UWBM 44304 | KBPBR287-07 | GQ481885 |
| *Ficedula albicilla* | UWBM 57971 | KBPBR210-07 | GQ481886 |
| *Ficedula albicilla* | UWBM 73122 | KBPBR072-07 | GQ481887 |
| *Ficedula albicilla* | UWBM 43997 | KBPBR157-07 | GQ481888 |
| *Ficedula albicilla* | UWBM 60169 | KBPBR230-07 | GQ481889 |
| *Ficedula albicilla* | UWBM 82281 | KBPBR496-07 | GQ481890 |
| *Ficedula albicilla* | UWBM 60049 | KBPBR224-07 | GQ481891 |
| *Ficedula albicollis* | UWBM 49299 | KBPBR083-07 | GQ481892 |
| *Ficedula albicollis* | UWBM 49388 | KBPBR095-07 | GQ481893 |
| *Ficedula albicollis* | UWBM 49425 | KBPBR099-07 | GQ481894 |
| *Ficedula hyperythra* | UWBM 67484 | KBPBR532-07 | GQ481895 |
| *Ficedula hypoleuca* | UWBM 49352 | KBPBR089-07 | GQ481896 |
| *Ficedula hypoleuca* | ZMMU 10a | KBPZM001-06 | GQ481897 |
| *Ficedula hypoleuca* | UWBM 49395 | KBPBR096-07 | GQ481898 |
| *Ficedula hypoleuca* | UWBM 49648 | KBPBR123-07 | GQ481899 |
| *Ficedula hypoleuca* | UWBM 49474 | KBPBR108-07 | GQ481900 |
| *Ficedula hypoleuca* | UWBM 61029 | KBPBR273-07 | GQ481901 |
| *Ficedula mugimaki* | UWBM 75175 | KBPBR362-07 | GQ481902 |
| *Ficedula mugimaki* | UWBM 78247 | KBPBR231-07 | GQ481903 |
| *Ficedula mugimaki* | UWBM 58451 | KBPBR529-07 | GQ481904 |
| *Ficedula mugimaki* | UWBM 46877 | KBPBR076-07 | GQ481905 |
| *Ficedula mugimaki* | UWBM 47595 | KBPBR521-07 | GQ481906 |
| *Ficedula mugimaki* | UWBM 72225 | KBPBR492-07 | GQ481907 |
| *Ficedula narcissina* | UWBM 47583 | KBPBR519-07 | GQ481908 |
| *Ficedula narcissina* | UWBM 46988 | KBPBR077-07 | GQ481909 |
| *Ficedula narcissina* | UWBM 47253 | KBPBR388-07 | GQ481910 |
| *Ficedula parva* | UWBM 49476 | KBPBR109-07 | GQ481911 |
| *Ficedula parva* | UWBM 64648 | KBPBR463-07 | GQ481912 |
| *Ficedula semitorquata* | UWBM 61130 | KBPBR275-07 | GQ481913 |
| *Ficedula semitorquata* | UWBM 61175 | KBPBR277-07 | GQ481914 |
| *Ficedula semitorquata* | UWBM 64706 | KBPBR470-07 | GQ481915 |
| *Ficedula semitorquata* | UWBM 64718 | KBPBR472-07 | GQ481916 |
| *Ficedula zanthopygia* | UWBM 71732 | KBPBR236-07 | GQ481917 |
| *Ficedula zanthopygia* | UWBM 74729 | KBPBR397-07 | GQ481918 |
| *Ficedula zanthopygia* | UWBM 59913 | KBPBR185-07 | GQ481919 |
| *Ficedula zanthopygia* | UWBM 47518 | KBPBR513-07 | GQ481920 |
| *Ficedula zanthopygia* | UWBM 74826 | KBPBR410-07 | GQ481921 |
| *Fratercula cirrhata* | UWBM 44103 | KBPBU358-06 | GQ481922 |
| *Fratercula cirrhata* | UWBM 44431 | KBPBU359-06 | GQ481923 |
| *Fringilla coelebs* | ZMMU 20a | KBPZM008-06 | GQ481924 |
| *Fringilla coelebs* | ZMMU 27a | KBPZM015-06 | GQ481925 |
| *Fringilla coelebs* | UWBM 49518 | KBPBK025-08 | GQ481926 |
| *Fringilla coelebs* | UWBM 51787 | KBPBK285-08 | GQ481927 |
| *Fringilla montifringilla* | UWBM 51184 | KBPBK083-08 | GQ481928 |
| *Fringilla montifringilla* | UWBM 44000 | KBPBK073-08 | GQ481929 |
| *Fringilla montifringilla* | UWBM 49761 | KBPBK037-08 | GQ481930 |
| *Fringilla montifringilla* | UWBM 78370 | KBPBK233-08 | GQ481931 |
| *Fringilla montifringilla* | UWBM 82282 | KBPBK333-08 | GQ481932 |
| *Fringilla montifringilla* | UWBM 56507 | KBPBK040-08 | GQ481933 |
| *Fringilla montifringilla* | UWBM 46622 | KBPBK150-08 | GQ481934 |
| *Fringilla montifringilla* | UWBM 67636 | KBPBK182-08 | GQ481935 |
| *Fringilla montifringilla* | UWBM 44561 | KBPBK255-08 | GQ481936 |
| *Fringilla montifringilla* | UWBM 59521 | KBPBK295-08 | GQ481937 |
| *Fulica atra* | UWBM 71979 | KBPBU132-06 | GQ481938 |
| *Galerida cristata* | UWBM 61358 | KBPBU515-06 | GQ481939 |
| *Galerida cristata* | UWBM 46385 | KBPBU512-06 | GQ481940 |
| *Galerida cristata* | UWBM 61329 | KBPBU513-06 | GQ481941 |
| *Galerida cristata* | UWBM 56595 | KBPBU511-06 | GQ481942 |
| *Gallinago gallinago* | UWBM 59432 | KBPBU272-06 | GQ481943 |
| *Gallinago gallinago* | UWBM 46880 | KBPBU269-06 | GQ481944 |
| *Gallinago gallinago* | UWBM 44046 | KBPBU270-06 | GQ481945 |
| *Gallinago gallinago* | UWBM 67701 | KBPBU271-06 | GQ481946 |
| *Gallinago media* | UWBM 61578 | KBPBU284-06 | GQ481947 |
| *Gallinago media* | UWBM 56761 | KBPBU283-06 | GQ481948 |
| *Gallinago megala* | UWBM 57929 | KBPBU274-06 | GQ481949 |
| *Gallinago megala* | UWBM 57927 | KBPBU275-06 | GQ481950 |
| *Gallinago stenura* | UWBM 57870 | KBPBU276-06 | GQ481951 |
| *Gallinago stenura* | UWBM 51675 | KBPBU279-06 | GQ481952 |
| *Gallinago stenura* | UWBM 67645 | KBPBU278-06 | GQ481953 |
| *Gallinago stenura* | UWBM 51859 | KBPBU280-06 | GQ481954 |
| *Gallinago stenura* | UWBM 46307 | KBPBU277-06 | GQ481955 |
| *Gallinula chloropus* | ZMMU RYA 1902 | KBPZM232-07 | GQ481956 |
| *Garrulus glandarius* | UWBM 49468 | KBPBK021-08 | GQ481957 |
| *Garrulus glandarius* | UWBM 61129 | KBPBK160-08 | GQ481958 |
| *Garrulus glandarius* | UWBM 58442 | KBPBK355-08 | GQ481959 |
| *Garrulus glandarius* | UWBM 72214 | KBPBK328-08 | GQ481960 |
| *Garrulus glandarius* | UWBM 75396 | KBPBK245-08 | GQ481961 |
| *Garrulus glandarius* | UWBM 57242 | KBPBK350-08 | GQ481962 |
| *Garrulus glandarius* | UWBM 74429 | KBPBK203-08 | GQ481963 |
| *Gavia stellata* | UWBM 44652 | KBPBU001-06 | GQ481964 |
| *Gavia stellata* | UWBM 56951 | KBPBU002-06 | GQ481965 |
| *Glareola nordmanni* | UWBM 56661 | KBPBU187-06 | GQ481966 |
| *Glareola nordmanni* | UWBM 56900 | KBPBU190-06 | GQ481967 |
| *Glareola nordmanni* | UWBM 56675 | KBPBU188-06 | GQ481968 |
| *Glareola pratincola* | UWBM 46260 | KBPBU193-06 | GQ481969 |
| *Glareola pratincola* | UWBM 46259 | KBPBU192-06 | GQ481970 |
| *Haematopus ostralegus* | UWBM 49759 | KBPBU158-06 | GQ481971 |
| *Haematopus ostralegus* | UWBM 56916 | KBPBU159-06 | GQ481972 |
| *Haematopus ostralegus* | UWBM 49540 | KBPBU157-06 | GQ481973 |
| *Hippolais caligata* | UWBM 46510 | KBPBR249-07 | GQ481974 |
| *Hippolais caligata* | UWBM 46386 | KBPBR201-07 | GQ481975 |
| *Hippolais icterina* | UWBM 49226 | KBPBR079-07 | GQ481976 |
| *Hippolais icterina* | UWBM 49415 | KBPBR098-07 | GQ481977 |
| *Hippolais icterina* | UWBM 49767 | KBPBR129-07 | GQ481978 |
| *Hirundapus caudacutus* | UWBM 47344 | KBPBU429-06 | GQ481979 |
| *Hirundapus caudacutus* | UWBM 47229 | KBPBU428-06 | GQ481980 |
| *Hirundapus caudacutus* | UWBM 47444 | KBPBU430-06 | GQ481981 |
| *Hirundo rustica* | UWBM 60079 | KBPBU542-06 | GQ481982 |
| *Hirundo rustica* | UWBM 44441 | KBPBU545-06 | GQ481983 |
| *Hirundo rustica* | UWBM 47366 | KBPBU543-06 | GQ481984 |
| *Hirundo rustica* | UWBM 46396 | KBPBU541-06 | GQ481985 |
| *Hirundo rustica* | UWBM 61435 | KBPBU544-06 | GQ481986 |
| *Histrionicus histrionicus* | UWBM 51871 | KBPBU055-06 | GQ481987 |
| *Histrionicus histrionicus* | UWBM 44446 | KBPBU054-06 | GQ481988 |
| *Ixobrychus eurhythmus* | UWBM 71976 | KBPBU017-06 | GQ481989 |
| *Ixobrychus eurhythmus* | UWBM 47046 | KBPBU015-06 | GQ481990 |
| *Ixobrychus eurhythmus* | UWBM 47126 | KBPBU019-06 | GQ481991 |
| *Ixobrychus eurhythmus* | UWBM 71975 | KBPBU016-06 | GQ481992 |
| *Ixobrychus eurhythmus* | UWBM 71990 | KBPBU018-06 | GQ481993 |
| *Ixobrychus minutus* | UWBM 56888 | KBPBU022-06 | GQ481994 |
| *Jynx torquilla* | UWBM 49209 | KBPBU496-06 | GQ481995 |
| *Lagopus lagopus* | UWBM 49717 | KBPBU085-06 | GQ481996 |
| *Lagopus lagopus* | UWBM 58884 | KBPBU089-06 | GQ481997 |
| *Lagopus lagopus* | UWBM 47351 | KBPBU087-06 | GQ481998 |
| *Lagopus lagopus* | UWBM 57879 | KBPBU092-06 | GQ481999 |
| *Lagopus lagopus* | UWBM 46576 | KBPBU086-06 | GQ482000 |
| *Lagopus muta* | UWBM 75674 | KBPBU094-06 | GQ482001 |
| *Lagopus muta* | UWBM 51814 | KBPBU091-06 | GQ482002 |
| *Lagopus muta* | UWBM 75664 | KBPBU093-06 | GQ482003 |
| *Lagopus muta* | UWBM 51661 | KBPBU090-06 | GQ482004 |
| *Lanius bucephalus* | ZMMU RYA 1595 | KBPZM127-07 | GQ482005 |
| *Lanius bucephalus* | ZMMU RYA 1199 | KBPZM100-07 | GQ482006 |
| *Lanius bucephalus* | ZMMU RYA 1594 | KBPZM126-07 | GQ482007 |
| *Lanius collurio* | UWBM 60985 | KBPBU613-06 | GQ482008 |
| *Lanius collurio* | UWBM 61097 | KBPBU611-06 | GQ482009 |
| *Lanius collurio* | UWBM 56842 | KBPBU610-06 | GQ482010 |
| *Lanius collurio* | UWBM 49202 | KBPBU612-06 | GQ482011 |
| *Lanius cristatus* | UWBM 67594 | KBPBU617-06 | GQ482012 |
| *Lanius cristatus* | UWBM 52595 | KBPBU616-06 | GQ482013 |
| *Lanius cristatus* | UWBM 47049 | KBPBU615-06 | GQ482014 |
| *Lanius excubitor* | UWBM 47014 | KBPBU619-06 | GQ482015 |
| *Lanius excubitor* | UWBM 57238 | KBPBU618-06 | GQ482016 |
| *Lanius excubitor* | UWBM 59641 | KBPBU620-06 | GQ482017 |
| *Lanius isabellinus* | UWBM 46496 | KBPBU621-06 | GQ482018 |
| *Lanius isabellinus* | UWBM 60139 | KBPBU614-06 | GQ482019 |
| *Lanius isabellinus* | UWBM 46630 | KBPBU622-06 | GQ482020 |
| *Lanius minor* | UWBM 56645 | KBPBU623-06 | GQ482021 |
| *Lanius minor* | UWBM 56922 | KBPBU624-06 | GQ482022 |
| *Lanius minor* | UWBM 61076 | KBPBU625-06 | GQ482023 |
| *Lanius minor* | UWBM 61082 | KBPBU626-06 | GQ482024 |
| *Lanius tigrinus* | UWBM 72082 | KBPBU627-06 | GQ482025 |
| *Larus canus* | UWBM 43929 | KBPBU307-06 | GQ482026 |
| *Larus canus* | UWBM 44309 | KBPBU311-06 | GQ482027 |
| *Larus canus* | UWBM 44196 | KBPBU310-06 | GQ482028 |
| *Larus canus* | UWBM 61302 | KBPBU309-06 | GQ482029 |
| *Larus canus* | UWBM 44068 | KBPBU308-06 | GQ482030 |
| *Larus crassirostris* | UWBM 51177 | KBPBU314-06 | GQ482031 |
| *Larus crassirostris* | UWBM 47274 | KBPBU315-06 | GQ482032 |
| *Larus crassirostris* | UWBM 46971 | KBPBU312-06 | GQ482033 |
| *Larus crassirostris* | UWBM 51176 | KBPBU313-06 | GQ482034 |
| *Larus heuglini* | UWBM 56820 | KBPBU304-06 | GQ482035 |
| *Larus hyperboreus* | UWBM 43852 | KBPBU319-06 | GQ482036 |
| *Larus hyperboreus* | UWBM 43851 | KBPBU318-06 | GQ482037 |
| *Larus ridibundus* | UWBM 43840 | KBPBU321-06 | GQ482038 |
| *Larus ridibundus* | UWBM 59734 | KBPBU325-06 | GQ482039 |
| *Larus ridibundus* | UWBM 44057 | KBPBU322-06 | GQ482040 |
| *Larus vegae* | UWBM 71997 | KBPBU305-06 | GQ482041 |
| *Larus vegae* | UWBM 73195 | KBPBU789-06 | GQ482042 |
| *Larus vegae* | UWBM 59969 | KBPBU306-06 | GQ482043 |
| *Leucosticte arctoa* | UWBM 57878 | KBPBK050-08 | GQ482044 |
| *Leucosticte arctoa* | UWBM 66525 | KBPBK218-08 | GQ482045 |
| *Leucosticte arctoa* | UWBM 44623 | KBPBK258-08 | GQ482046 |
| *Leucosticte arctoa* | UWBM 46607 | KBPBK149-08 | GQ482047 |
| *Leucosticte brandti* | UWBM 66515 | KBPBK217-08 | GQ482048 |
| *Leucosticte brandti* | UWBM 75793 | KBPBK189-08 | GQ482049 |
| *Leucosticte nemoricola* | UWBM 66713 | KBPBK320-08 | GQ482050 |
| *Leucosticte nemoricola* | UWBM 75644 | KBPBK193-08 | GQ482051 |
| *Leucosticte nemoricola* | UWBM 75860 | KBPBK191-08 | GQ482052 |
| *Leucosticte nemoricola* | UWBM 66572 | KBPBK224-08 | GQ482053 |
| *Limicola falcinellus* | UWBM 44493 | KBPBU229-06 | GQ482054 |
| *Limnodromus scolopaceus* | UWBM 43936 | KBPBU292-06 | GQ482055 |
| *Limnodromus scolopaceus* | UWBM 43928 | KBPBU290-06 | GQ482056 |
| *Limnodromus scolopaceus* | UWBM 43930 | KBPBU291-06 | GQ482057 |
| *Limosa lapponica* | UWBM 49751 | KBPBU155-06 | GQ482058 |
| *Limosa lapponica* | UWBM 49752 | KBPBU156-06 | GQ482059 |
| *Limosa lapponica* | UWBM 49665 | KBPBU154-06 | GQ482060 |
| *Limosa lapponica* | UWBM 49664 | KBPBU153-06 | GQ482061 |
| *Limosa limosa* | UWBM 73771 | KBPBU780-06 | GQ482062 |
| *Limosa limosa* | UWBM 46884 | KKBNA290-05 | GQ482063 |
| *Limosa limosa* | UWBM 73550 | KBPBU779-06 | GQ482064 |
| *Limosa limosa* | UWBM 46888 | KBPBU152-06 | GQ482065 |
| *Limosa limosa* | UWBM 47118 | KKBNA728-05 | GQ482066 |
| *Locustella amnicola* | UWBM 47496 | KBPBR508-07 | GQ482067 |
| *Locustella amnicola* | UWBM 47557 | KBPBR516-07 | GQ482068 |
| *Locustella certhiola* | UWBM 47180 | KBPBR386-07 | GQ482069 |
| *Locustella certhiola* | UWBM 67713 | KBPBR302-07 | GQ482070 |
| *Locustella certhiola* | UWBM 51798 | KBPBR426-07 | GQ482071 |
| *Locustella certhiola* | UWBM 73425 | KBPBR346-07 | GQ482072 |
| *Locustella certhiola* | UWBM 43834 | KBPBR155-07 | GQ482073 |
| *Locustella certhiola* | UWBM 73556 | KBPBR351-07 | GQ482074 |
| *Locustella certhiola* | UWBM 72154 | KBPBR487-07 | GQ482075 |
| *Locustella fasciolata* | UWBM 72266 | KBPBR493-07 | GQ482076 |
| *Locustella fluviatilis* | UWBM 49471 | KBPBR106-07 | GQ482077 |
| *Locustella fluviatilis* | UWBM 82215 | KBPBR494-07 | GQ482078 |
| *Locustella lanceolata* | UWBM 49463 | KBPBR104-07 | GQ482079 |
| *Locustella lanceolata* | ZMMU RYA 1929 | KBPZM240-07 | GQ482080 |
| *Locustella lanceolata* | UWBM 57934 | KBPBR144-07 | GQ482081 |
| *Locustella lanceolata* | UWBM 73564 | KBPBR352-07 | GQ482082 |
| *Locustella lanceolata* | UWBM 83396 | KBPBR373-07 | GQ482083 |
| *Locustella lanceolata* | UWBM 44157 | KBPBR198-07 | GQ482084 |
| *Locustella lanceolata* | UWBM 59958 | KBPBR187-07 | GQ482085 |
| *Locustella lanceolata* | ZMMU RYA 1724 | KBPZM185-07 | GQ482086 |
| *Locustella lanceolata* | ZMMU RYA 1798 | KBPZM214-07 | GQ482087 |
| *Locustella naevia* | UWBM 67725 | KBPBR304-07 | GQ482088 |
| *Locustella naevia* | UWBM 64744 | KBPBR474-07 | GQ482089 |
| *Locustella naevia* | UWBM 61505 | KBPBR457-07 | GQ482090 |
| *Locustella naevia* | UWBM 61451 | KBPBR453-07 | GQ482091 |
| *Locustella ochotensis* | UWBM 44066 | KBPBR160-07 | GQ482092 |
| *Locustella ochotensis* | UWBM 47260 | KBPBR390-07 | GQ482093 |
| *Locustella ochotensis* | ZMMU RYA 1662 | KBPZM145-07 | GQ482094 |
| *Locustella ochotensis* | ZMMU RYA 1663 | KBPZM146-07 | GQ482095 |
| *Locustella ochotensis* | ZMMU RYA 1671 | KBPZM147-07 | GQ482096 |
| *Locustella ochotensis* | ZMMU RYA 1660 | KBPZM143-07 | GQ482097 |
| *Locustella ochotensis* | ZMMU RYA 1661 | KBPZM144-07 | GQ482098 |
| *Lophophanes cristatus* | UWBM 57235 | KBPBR523-07 | GQ482099 |
| *Lophophanes cristatus* | UWBM 49778 | KBPBR131-07 | GQ482100 |
| *Lophophanes cristatus* | ZMMU RYA 1427 | KBPZM114-07 | GQ482101 |
| *Loxia curvirostra* | UWBM 49614 | KBPBK031-08 | GQ482102 |
| *Loxia curvirostra* | UWBM 46981 | KBPBK010-08 | GQ482103 |
| *Loxia curvirostra* | UWBM 49723 | KBPBK034-08 | GQ482104 |
| *Loxia curvirostra* | UWBM 61211 | KBPBK164-08 | GQ482105 |
| *Loxia curvirostra* | UWBM 74302 | KBPBK204-08 | GQ482106 |
| *Loxia leucoptera* | UWBM 59497 | KBPBK211-08 | GQ482107 |
| *Loxia leucoptera* | UWBM 56800 | KBPBK095-08 | GQ482108 |
| *Loxia leucoptera* | UWBM 56799 | KBPBK094-08 | GQ482109 |
| *Lullula arborea* | UWBM 64680 | KBPBU509-06 | GQ482110 |
| *Lullula arborea* | UWBM 64681 | KBPBU510-06 | GQ482111 |
| *Luscinia calliope* | UWBM 44150 | KBPBR030-06 | GQ482112 |
| *Luscinia calliope* | UWBM 51743 | KBPBR060-06 | GQ482113 |
| *Luscinia calliope* | UWBM 47214 | KBPBR053-06 | GQ482114 |
| *Luscinia calliope* | UWBM 52532 | KBPBR058-06 | GQ482115 |
| *Luscinia calliope* | UWBM 73298 | KBPBR041-06 | GQ482116 |
| *Luscinia calliope* | UWBM 59869 | KBPBR028-06 | GQ482117 |
| *Luscinia calliope* | ZMMU RYA 1681 | KBPZM155-07 | GQ482118 |
| *Luscinia calliope* | ZMMU RYA 1682 | KBPZM156-07 | GQ482119 |
| *Luscinia calliope* | ZMMU RYA 1680 | KBPZM154-07 | GQ482120 |
| *Luscinia calliope* | ZMMU RYA 1658 | KBPZM141-07 | GQ482121 |
| *Luscinia cyane* | UWBM 47130 | KBPBR051-06 | GQ482122 |
| *Luscinia cyane* | UWBM 74757 | KBPBR056-06 | GQ482123 |
| *Luscinia cyane* | UWBM 52522 | KBPBR057-06 | GQ482124 |
| *Luscinia cyane* | UWBM 51739 | KBPBR059-06 | GQ482125 |
| *Luscinia cyane* | UWBM 46940 | KBPBR001-06 | GQ482126 |
| *Luscinia cyane* | UWBM 59709 | KBPBR034-06 | GQ482127 |
| *Luscinia luscinia* | UWBM 49577 | KBPBR016-06 | GQ482128 |
| *Luscinia luscinia* | UWBM 49179 | KBPBR005-06 | GQ482129 |
| *Luscinia luscinia* | UWBM 49411 | KBPBR010-06 | GQ482130 |
| *Luscinia luscinia* | UWBM 49514 | KBPBR014-06 | GQ482131 |
| *Luscinia luscinia* | UWBM 74235 | KBPBR045-06 | GQ482132 |
| *Luscinia luscinia* | UWBM 59669 | KBPBR062-06 | GQ482133 |
| *Luscinia luscinia* | ZMMU 59a | KBPZM027-06 | GQ482134 |
| *Luscinia megarhynchos* | UWBM 64638 | KBPBR064-06 | GQ482135 |
| *Luscinia megarhynchos* | UWBM 46491 | KBPBR037-06 | GQ482136 |
| *Luscinia megarhynchos* | UWBM 61111 | KBPBR039-06 | GQ482137 |
| *Luscinia sibilans* | UWBM 47493 | KBPBR068-06 | GQ482138 |
| *Luscinia sibilans* | UWBM 44562 | KBPBR049-06 | GQ482139 |
| *Luscinia sibilans* | UWBM 78240 | KBPBR036-06 | GQ482140 |
| *Luscinia sibilans* | UWBM 47106 | KBPBR026-06 | GQ482141 |
| *Luscinia svecica* | UWBM 74242 | KBPBR044-06 | GQ482142 |
| *Luscinia svecica* | UWBM 59422 | KBPBR047-06 | GQ482143 |
| *Luscinia svecica* | UWBM 49697 | KBPBR017-06 | GQ482144 |
| *Luscinia svecica* | UWBM 75800 | KBPBR043-06 | GQ482145 |
| *Luscinia svecica* | UWBM 44132 | KBPBR029-06 | GQ482146 |
| *Luscinia svecica* | ZMMU RYA 1926 | KBPZM237-07 | GQ482147 |
| *Luscinia svecica* | ZMMU RYA 1927 | KBPZM238-07 | GQ482148 |
| *Lymnocryptes minimus* | UWBM 68121 | KBPBU281-06 | GQ482149 |
| *Lymnocryptes minimus* | UWBM 68120 | KBPBU282-06 | GQ482150 |
| *Melanitta fusca* | UWBM 44372 | KBPBU056-06 | GQ482151 |
| *Melanitta nigra* | UWBM 43946 | KBPBU057-06 | GQ482152 |
| *Melanocorypha mongolica* | UWBM 66599 | KBPBU533-06 | GQ482153 |
| *Melanocorypha mongolica* | UWBM 60020 | KBPBU531-06 | GQ482154 |
| *Melanocorypha mongolica* | UWBM 59786 | KBPBU530-06 | GQ482155 |
| *Melanocorypha mongolica* | UWBM 67599 | KBPBU532-06 | GQ482156 |
| *Melanocorypha mongolica* | UWBM 57847 | KBPBU529-06 | GQ482157 |
| *Mergus serrator* | UWBM 59567 | KBPBU058-06 | GQ482158 |
| *Mergus squamatus* | UWBM 72195 | KBPBU059-06 | GQ482159 |
| *Merops apiaster* | UWBM 61073 | KBPBU446-06 | GQ482160 |
| *Merops apiaster* | UWBM 49370 | KBPBU444-06 | GQ482161 |
| *Merops apiaster* | UWBM 49309 | KBPBU443-06 | GQ482162 |
| *Merops apiaster* | UWBM 61455 | KBPBU447-06 | GQ482163 |
| *Merops apiaster* | UWBM 56656 | KBPBU445-06 | GQ482164 |
| *Monticola gularis* | UWBM 72202 | KBPBU688-06 | GQ482165 |
| *Monticola gularis* | UWBM 72203 | KBPBU689-06 | GQ482166 |
| *Monticola gularis* | UWBM 71689 | KBPBU686-06 | GQ482167 |
| *Monticola gularis* | UWBM 59864 | KBPBU685-06 | GQ482168 |
| *Monticola gularis* | UWBM 47213 | KBPBU687-06 | GQ482169 |
| *Monticola saxatilis* | UWBM 67602 | KBPBU693-06 | GQ482170 |
| *Monticola saxatilis* | UWBM 57880 | KBPBU690-06 | GQ482171 |
| *Monticola saxatilis* | UWBM 46517 | KBPBU692-06 | GQ482172 |
| *Monticola saxatilis* | UWBM 64759 | KBPBU694-06 | GQ482173 |
| *Montifringilla davidiana* | UWBM 57837 | KBPBK046-08 | GQ482174 |
| *Montifringilla davidiana* | UWBM 57838 | KBPBK047-08 | GQ482175 |
| *Montifringilla nivalis* | UWBM 57874 | KBPBK049-08 | GQ482176 |
| *Montifringilla nivalis* | UWBM 75743 | KBPBK187-08 | GQ482177 |
| *Montifringilla nivalis* | UWBM 75733 | KBPBK186-08 | GQ482178 |
| *Motacilla alba* | ZMMU RYA 1632 | KBPZM136-07 | GQ482179 |
| *Motacilla alba* | UWBM 46962 | KBPBR002-06 | GQ482180 |
| *Motacilla alba* | UWBM 44605 | KBPBR050-06 | GQ482181 |
| *Motacilla alba* | UWBM 47243 | KBPBR054-06 | GQ482182 |
| *Motacilla alba* | UWBM 46991 | KBPBR004-06 | GQ482183 |
| *Motacilla alba* | UWBM 49525 | KBPBR015-06 | GQ482184 |
| *Motacilla alba* | UWBM 49718 | KBPBR018-06 | GQ482185 |
| *Motacilla alba* | UWBM 46419 | KBPBR032-06 | GQ482186 |
| *Motacilla alba* | UWBM 49417 | KBPBR011-06 | GQ482187 |
| *Motacilla alba* | ZMMU RYA 1635 | KBPZM138-07 | GQ482188 |
| *Motacilla alba* | ZMMU RYA 1636 | KBPZM139-07 | GQ482189 |
| *Motacilla alba* | ZMMU RYA 1653 | KBPZM140-07 | GQ482190 |
| *Motacilla alba* | UWBM 43837 | KBPBR023-06 | GQ482191 |
| *Motacilla cinerea* | UWBM 46556 | KBPBR038-06 | GQ482192 |
| *Motacilla cinerea* | UWBM 47196 | KBPBR052-06 | GQ482193 |
| *Motacilla cinerea* | UWBM 43826 | KBPBR022-06 | GQ482194 |
| *Motacilla cinerea* | UWBM 46439 | KBPBR033-06 | GQ482195 |
| *Motacilla cinerea* | UWBM 44026 | KBPBR025-06 | GQ482196 |
| *Motacilla cinerea* | ZMMU RYA 1584 | KBPZM122-07 | GQ482197 |
| *Motacilla citreola* | UWBM 46337 | KBPBR021-06 | GQ482198 |
| *Motacilla citreola* | UWBM 49351 | KBPBR009-06 | GQ482199 |
| *Motacilla citreola* | UWBM 46336 | KBPBR020-06 | GQ482200 |
| *Motacilla citreola* | UWBM 49348 | KBPBR007-06 | GQ482201 |
| *Motacilla citreola* | UWBM 49350 | KBPBR008-06 | GQ482202 |
| *Motacilla citreola* | ZMMU RYA 1928 | KBPZM239-07 | GQ482203 |
| *Motacilla flava* | UWBM 46975 | KBPBR003-06 | GQ482204 |
| *Motacilla flava* | UWBM 49434 | KBPBR012-06 | GQ482205 |
| *Motacilla flava* | UWBM 46881 | KKBNA289-05 | GQ482206 |
| *Motacilla tschutschensis* | UWBM 43941 | KBPBR024-06 | GQ482207 |
| *Motacilla tschutschensis* | UWBM 44480 | KBPBR048-06 | GQ482208 |
| *Muscicapa dauurica* | UWBM 57861 | KBPBR141-07 | GQ482209 |
| *Muscicapa dauurica* | UWBM 74791 | KBPBR403-07 | GQ482210 |
| *Muscicapa dauurica* | UWBM 73228 | KBPBR069-07 | GQ482211 |
| *Muscicapa dauurica* | UWBM 60104 | KBPBR225-07 | GQ482212 |
| *Muscicapa dauurica* | UWBM 47426 | KBPBR502-07 | GQ482213 |
| *Muscicapa dauurica* | ZMMU RYA 1722 | KBPZM183-07 | GQ482214 |
| *Muscicapa dauurica* | ZMMU RYA 1828 | KBPZM222-07 | GQ482215 |
| *Muscicapa dauurica* | ZMMU RYA 1846 | KBPZM228-07 | GQ482216 |
| *Muscicapa griseisticta* | UWBM 58404 | KBPBR525-07 | GQ482217 |
| *Muscicapa griseisticta* | UWBM 44071 | KBPBR161-07 | GQ482218 |
| *Muscicapa griseisticta* | UWBM 44145 | KBPBR196-07 | GQ482219 |
| *Muscicapa sibirica* | UWBM 83285 | KBPBR379-07 | GQ482220 |
| *Muscicapa sibirica* | UWBM 73106 | KBPBR070-07 | GQ482221 |
| *Muscicapa sibirica* | UWBM 44571 | KBPBR383-07 | GQ482222 |
| *Muscicapa sibirica* | UWBM 44282 | KBPBR286-07 | GQ482223 |
| *Muscicapa sibirica* | UWBM 59998 | KBPBR193-07 | GQ482224 |
| *Muscicapa sibirica* | UWBM 74629 | KBPBR265-07 | GQ482225 |
| *Muscicapa sibirica* | UWBM 58009 | KBPBR216-07 | GQ482226 |
| *Muscicapa striata* | UWBM 49509 | KBPBR113-07 | GQ482227 |
| *Muscicapa striata* | UWBM 49762 | KBPBR128-07 | GQ482228 |
| *Muscicapa striata* | UWBM 49188 | KBPBR078-07 | GQ482229 |
| *Muscicapa striata* | UWBM 46487 | KBPBR245-07 | GQ482230 |
| *Muscicapa striata* | UWBM 49400 | KBPBR097-07 | GQ482231 |
| *Muscicapa striata* | UWBM 60991 | KBPBR268-07 | GQ482232 |
| *Muscicapa striata* | UWBM 64853 | KBPBR478-07 | GQ482233 |
| *Netta rufina* | UWBM 56423 | KBPBU060-06 | GQ482234 |
| *Nucifraga caryocatactes* | UWBM 46286 | KBPBK067-08 | GQ482235 |
| *Nucifraga caryocatactes* | UWBM 56746 | KBPBK090-08 | GQ482236 |
| *Nucifraga caryocatactes* | UWBM 43817 | KBPBK069-08 | GQ482237 |
| *Nucifraga caryocatactes* | UWBM 75159 | KBPBK241-08 | GQ482238 |
| *Nucifraga caryocatactes* | UWBM 73681 | KBPBK344-08 | GQ482239 |
| *Nucifraga caryocatactes* | UWBM 59995 | KBPBK105-08 | GQ482240 |
| *Nucifraga caryocatactes* | UWBM 44365 | KBPBK176-08 | GQ482241 |
| *Nucifraga caryocatactes* | UWBM 66384 | KBPBK210-08 | GQ482242 |
| *Nucifraga caryocatactes* | UWBM 47598 | KBPBK349-08 | GQ482243 |
| *Numenius arquata* | UWBM 46473 | KBPBU141-06 | GQ482244 |
| *Numenius arquata* | UWBM 46329 | KBPBU140-06 | GQ482245 |
| *Numenius madagascariensis* | UWBM 46885 | KBPBU142-06 | GQ482246 |
| *Numenius madagascariensis* | UWBM 46886 | KBPBU145-06 | GQ482247 |
| *Numenius madagascariensis* | UWBM 47183 | KBPBU146-06 | GQ482248 |
| *Numenius madagascariensis* | UWBM 47020 | KBPBU143-06 | GQ482249 |
| *Numenius madagascariensis* | UWBM 47043 | KBPBU144-06 | GQ482250 |
| *Numenius phaeopus* | UWBM 59485 | KBPBU150-06 | GQ482251 |
| *Numenius phaeopus* | UWBM 49694 | KBPBU148-06 | GQ482252 |
| *Numenius phaeopus* | UWBM 49676 | KBPBU147-06 | GQ482253 |
| *Numenius phaeopus* | UWBM 61337 | KBPBU151-06 | GQ482254 |
| *Numenius phaeopus* | UWBM 51181 | KBPBU149-06 | GQ482255 |
| *Oenanthe deserti* | UWBM 66654 | KBPBU699-06 | GQ482256 |
| *Oenanthe deserti* | UWBM 57961 | KBPBU695-06 | GQ482257 |
| *Oenanthe deserti* | UWBM 66493 | KBPBU698-06 | GQ482258 |
| *Oenanthe deserti* | UWBM 57976 | KBPBU697-06 | GQ482259 |
| *Oenanthe deserti* | UWBM 57975 | KBPBU696-06 | GQ482260 |
| *Oenanthe isabellina* | UWBM 59787 | KBPBU701-06 | GQ482261 |
| *Oenanthe isabellina* | UWBM 46461 | KBPBU703-06 | GQ482262 |
| *Oenanthe isabellina* | UWBM 56910 | KBPBU702-06 | GQ482263 |
| *Oenanthe isabellina* | UWBM 66620 | KBPBU704-06 | GQ482264 |
| *Oenanthe isabellina* | UWBM 46247 | KBPBU700-06 | GQ482265 |
| *Oenanthe oenanthe* | UWBM 59537 | KBPBU709-06 | GQ482266 |
| *Oenanthe oenanthe* | UWBM 44203 | KBPBU708-06 | GQ482267 |
| *Oenanthe oenanthe* | UWBM 46422 | KBPBU705-06 | GQ482268 |
| *Oenanthe oenanthe* | UWBM 49362 | KBPBU707-06 | GQ482269 |
| *Oenanthe oenanthe* | UWBM 57884 | KBPBU706-06 | GQ482270 |
| *Oenanthe pleschanka* | UWBM 46249 | KBPBU710-06 | GQ482271 |
| *Oenanthe pleschanka* | UWBM 60161 | KBPBU711-06 | GQ482272 |
| *Oenanthe pleschanka* | UWBM 57817 | KBPBU712-06 | GQ482273 |
| *Oenanthe pleschanka* | UWBM 64636 | KBPBU713-06 | GQ482274 |
| *Oenanthe pleschanka* | UWBM 66667 | KBPBU714-06 | GQ482275 |
| *Onychoprion aleuticus* | UWBM 47320 | KBPBU340-06 | GQ482276 |
| *Oriolus chinensis* | UWBM 71725 | KBPBK138-08 | GQ482277 |
| *Oriolus chinensis* | UWBM 72068 | KBPBK323-08 | GQ482278 |
| *Oriolus oriolus* | UWBM 49306 | KBPBK012-08 | GQ482279 |
| *Oriolus oriolus* | UWBM 56652 | KBPBK087-08 | GQ482280 |
| *Oriolus oriolus* | UWBM 61157 | KBPBK162-08 | GQ482281 |
| *Oriolus oriolus* | UWBM 61063 | KBPBK159-08 | GQ482282 |
| *Oriolus oriolus* | UWBM 51749 | KBPBK283-08 | GQ482283 |
| *Otus lettia* | UWBM 51123 | KBPBU393-06 | GQ482284 |
| *Otus lettia* | UWBM 57799 | KBPBU392-06 | GQ482285 |
| *Otus sunia* | UWBM 60071 | KBPBU394-06 | GQ482286 |
| *Otus sunia* | UWBM 51122 | KBPBU396-06 | GQ482287 |
| *Otus sunia* | UWBM 58434 | KBPBU395-06 | GQ482288 |
| *Otus sunia* | UWBM 51180 | KBPBU397-06 | GQ482289 |
| *Panurus biarmicus* | UWBM 67702 | KBPBR042-06 | GQ482290 |
| *Panurus biarmicus* | UWBM 56573 | KBPBR027-06 | GQ482291 |
| *Panurus biarmicus* | UWBM 59726 | KBPBR035-06 | GQ482292 |
| *Panurus biarmicus* | UWBM 61235 | KBPBR040-06 | GQ482293 |
| *Parus major* | UWBM 74796 | KBPBR404-07 | GQ482294 |
| *Parus major* | UWBM 51795 | KBPBR425-07 | GQ482295 |
| *Parus major* | UWBM 49480 | KBPBR110-07 | GQ482296 |
| *Parus major* | UWBM 56462 | KBPBR134-07 | GQ482297 |
| *Parus major* | UWBM 46626 | KBPBR262-07 | GQ482298 |
| *Parus major* | UWBM 80561 | KBPBR266-07 | GQ482299 |
| *Parus major* | UWBM 46513 | KBPBR250-07 | GQ482300 |
| *Parus major* | ZMMU RYA 1188 | KBPZM099-07 | GQ482301 |
| *Parus major* | ZMMU RYA 1064 | KBPZM092-07 | GQ482302 |
| *Parus major* | ZMMU RYA 1238 | KBPZM104-07 | GQ482303 |
| *Parus major* | ZMMU RYA 1596 | KBPZM128-07 | GQ482304 |
| *Parus major* | ZMMU ASF 15 | KBPZM069-07 | GQ482305 |
| *Parus major* | ZMMU 29a | KBPZM017-06 | GQ482306 |
| *Passer ammodendri* | UWBM 46393 | KBPBK110-08 | GQ482307 |
| *Passer domesticus* | UWBM 61303 | KBPBK155-08 | GQ482308 |
| *Passer domesticus* | UWBM 67750 | KBPBK237-08 | GQ482309 |
| *Passer domesticus* | UWBM 73278 | KBPBK001-08 | GQ482310 |
| *Passer domesticus* | UWBM 46504 | KBPBK145-08 | GQ482311 |
| *Passer domesticus* | UWBM 61131 | KBPBK161-08 | GQ482312 |
| *Passer domesticus* | UWBM 74328 | KBPBK197-08 | GQ482313 |
| *Passer hispaniolensis* | UWBM 46503 | KBPBK144-08 | GQ482314 |
| *Passer hispaniolensis* | UWBM 46502 | KBPBK143-08 | GQ482315 |
| *Passer montanus* | UWBM 56580 | KBPBK084-08 | GQ482316 |
| *Passer montanus* | UWBM 61403 | KBPBK304-08 | GQ482317 |
| *Passer montanus* | UWBM 60042 | KBPBK125-08 | GQ482318 |
| *Passer montanus* | UWBM 61304 | KBPBK156-08 | GQ482319 |
| *Passer montanus* | UWBM 58052 | KBPBK121-08 | GQ482320 |
| *Passer montanus* | UWBM 73694 | KBPBK345-08 | GQ482321 |
| *Passer montanus* | UWBM 46506 | KBPBK146-08 | GQ482322 |
| *Passer montanus* | UWBM 74636 | KBPBK154-08 | GQ482323 |
| *Passer montanus* | UWBM 67573 | KBPBK184-08 | GQ482324 |
| *Passer rutilans* | UWBM 47482 | KBPBK340-08 | GQ482325 |
| *Passer rutilans* | UWBM 47375 | KBPBK276-08 | GQ482326 |
| *Passer rutilans* | ZMMU EAK 107 | KBPZM080-07 | GQ482327 |
| *Passer rutilans* | ZMMU EAK 108 | KBPZM081-07 | GQ482328 |
| *Passer rutilans* | ZMMU EAK 103 | KBPZM076-07 | GQ482329 |
| *Perdix dauurica* | UWBM 66631 | KBPBU123-06 | GQ482330 |
| *Perdix dauurica* | UWBM 59982 | KBPBU122-06 | GQ482331 |
| *Perdix perdix* | UWBM 71365 | KKBNA748-05 | GQ482332 |
| *Pericrocotus divaricatus* | UWBM 72182 | KBPBU609-06 | GQ482333 |
| *Pericrocotus divaricatus* | UWBM 47237 | KBPBU608-06 | GQ482334 |
| *Periparus ater* | UWBM 47287 | KBPBR391-07 | GQ482335 |
| *Periparus ater* | UWBM 49451 | KBPBR102-07 | GQ482336 |
| *Periparus ater* | UWBM 57197 | KBPBR208-07 | GQ482337 |
| *Periparus ater* | UWBM 51772 | KBPBR424-07 | GQ482338 |
| *Periparus ater* | UWBM 46870 | KBPBR075-07 | GQ482339 |
| *Periparus ater* | UWBM 59999 | KBPBR194-07 | GQ482340 |
| *Periparus ater* | ZMMU RYA 1878 | KBPZM230-07 | GQ482341 |
| *Periparus ater* | ZMMU RYA 202 | KBPZM243-07 | GQ482342 |
| *Periparus ater* | ZMMU RYA 204 | KBPZM244-07 | GQ482343 |
| *Periparus ater* | ZMMU RYA 1874 | KBPZM229-07 | GQ482344 |
| *Periparus ater* | ZMMU RYA 1966 | KBPZM242-07 | GQ482345 |
| *Perisoreus infaustus* | UWBM 78274 | KBPBK135-08 | GQ482346 |
| *Perisoreus infaustus* | UWBM 51769 | KBPBK284-08 | GQ482347 |
| *Perisoreus infaustus* | UWBM 66538 | KBPBK222-08 | GQ482348 |
| *Perisoreus infaustus* | UWBM 51860 | KBPBK287-08 | GQ482349 |
| *Perisoreus infaustus* | UWBM 49730 | KBPBK035-08 | GQ482350 |
| *Perisoreus infaustus* | UWBM 44014 | KBPBK075-08 | GQ482351 |
| *Perisoreus infaustus* | UWBM 73489 | KBPBK232-08 | GQ482352 |
| *Perisoreus infaustus* | UWBM 56793 | KBPBK092-08 | GQ482353 |
| *Petronia petronia* | UWBM 78961 | KBPBK181-08 | GQ482354 |
| *Petronia petronia* | UWBM 66486 | KBPBK216-08 | GQ482355 |
| *Phalacrocorax pelagicus* | UWBM 47273 | KBPBU012-06 | GQ482356 |
| *Phalacrocorax pelagicus* | UWBM 47268 | KBPBU011-06 | GQ482357 |
| *Phalaropus lobatus* | UWBM 47002 | KBPBU198-06 | GQ482358 |
| *Phalaropus lobatus* | UWBM 43857 | KBPBU199-06 | GQ482359 |
| *Phalaropus lobatus* | UWBM 44235 | KBPBU200-06 | GQ482360 |
| *Phalaropus lobatus* | UWBM 59445 | KBPBU201-06 | GQ482361 |
| *Phasianus colchicus* | UWBM 46494 | KBPBU115-06 | GQ482362 |
| *Phasianus colchicus* | UWBM 75364 | KBPBU775-06 | GQ482363 |
| *Phasianus colchicus* | UWBM 74864 | KBPBU774-06 | GQ482364 |
| *Philomachus pugnax* | UWBM 56425 | KBPBU231-06 | GQ482365 |
| *Philomachus pugnax* | UWBM 59419 | KBPBU235-06 | GQ482366 |
| *Philomachus pugnax* | UWBM 43980 | KBPBU232-06 | GQ482367 |
| *Philomachus pugnax* | UWBM 44232 | KBPBU234-06 | GQ482368 |
| *Philomachus pugnax* | UWBM 56715 | KBPBU233-06 | GQ482369 |
| *Phoenicurus auroreus* | UWBM 60170 | KBPBU743-06 | GQ482370 |
| *Phoenicurus auroreus* | UWBM 46901 | KBPBU746-06 | GQ482371 |
| *Phoenicurus auroreus* | UWBM 59769 | KBPBU742-06 | GQ482372 |
| *Phoenicurus auroreus* | UWBM 47090 | KBPBU745-06 | GQ482373 |
| *Phoenicurus auroreus* | UWBM 46349 | KBPBU744-06 | GQ482374 |
| *Phoenicurus erythrogastrus* | UWBM 66513 | KBPBU753-06 | GQ482375 |
| *Phoenicurus erythrogastrus* | UWBM 66672 | KBPBU754-06 | GQ482376 |
| *Phoenicurus erythrogastrus* | UWBM 66354 | KBPBU752-06 | GQ482377 |
| *Phoenicurus erythronotus* | UWBM 66542 | KBPBU749-06 | GQ482378 |
| *Phoenicurus erythronotus* | UWBM 66678 | KBPBU751-06 | GQ482379 |
| *Phoenicurus erythronotus* | UWBM 67651 | KBPBU748-06 | GQ482380 |
| *Phoenicurus erythronotus* | UWBM 66557 | KBPBU750-06 | GQ482381 |
| *Phoenicurus erythronotus* | UWBM 46289 | KBPBU747-06 | GQ482382 |
| *Phoenicurus ochruros* | ZMMU 84a | KBPZM041-06 | GQ482383 |
| *Phoenicurus ochruros* | UWBM 66503 | KBPBU735-06 | GQ482384 |
| *Phoenicurus ochruros* | UWBM 67603 | KBPBU734-06 | GQ482385 |
| *Phoenicurus ochruros* | UWBM 64768 | KBPBU733-06 | GQ482386 |
| *Phoenicurus ochruros* | UWBM 66625 | KBPBU736-06 | GQ482387 |
| *Phoenicurus ochruros* | ZMMU 85a | KBPZM042-06 | GQ482388 |
| *Phoenicurus phoenicurus* | ZMMU 112a | KBPZM048-06 | GQ482389 |
| *Phoenicurus phoenicurus* | UWBM 58024 | KBPBU738-06 | GQ482390 |
| *Phoenicurus phoenicurus* | UWBM 61122 | KBPBU739-06 | GQ482391 |
| *Phoenicurus phoenicurus* | UWBM 56805 | KBPBU741-06 | GQ482392 |
| *Phoenicurus phoenicurus* | UWBM 46481 | KBPBU737-06 | GQ482393 |
| *Phoenicurus phoenicurus* | UWBM 67569 | KBPBU740-06 | GQ482394 |
| *Phylloscopus borealis* | UWBM 82284 | KBPBR498-07 | GQ482395 |
| *Phylloscopus borealis* | UWBM 59696 | KBPBR147-07 | GQ482396 |
| *Phylloscopus borealis* | UWBM 56532 | KBPBR137-07 | GQ482397 |
| *Phylloscopus borealis* | UWBM 52547 | KBPBR414-07 | GQ482398 |
| *Phylloscopus borealis* | UWBM 82938 | KBPBR380-07 | GQ482399 |
| *Phylloscopus borealis* | UWBM 66539 | KBPBR337-07 | GQ482400 |
| *Phylloscopus borealis* | UWBM 78398 | KBPBR356-07 | GQ482401 |
| *Phylloscopus borealis* | UWBM 51101 | KBPBR167-07 | GQ482402 |
| *Phylloscopus borealis* | UWBM 57973 | KBPBR211-07 | GQ482403 |
| *Phylloscopus borealis* | UWBM 59484 | KBPBR324-07 | GQ482404 |
| *Phylloscopus borealis* | ZMMU RYA 1567 | KBPZM119-07 | GQ482405 |
| *Phylloscopus borealis* | ZMMU RYA 1705 | KBPZM172-07 | GQ482406 |
| *Phylloscopus borealis* | ZMMU RYA 1659 | KBPZM142-07 | GQ482407 |
| *Phylloscopus borealis* | ZMMU RYA 1707 | KBPZM174-07 | GQ482408 |
| *Phylloscopus borealoides* | UWBM 47488 | KBPBR506-07 | GQ482409 |
| *Phylloscopus borealoides* | UWBM 83277 | KBPBR378-07 | GQ482410 |
| *Phylloscopus borealoides* | ZMMU RYA 1310 | KBPZM111-07 | GQ482411 |
| *Phylloscopus borealoides* | ZMMU RYA 1388 | KBPZM112-07 | GQ482412 |
| *Phylloscopus borealoides* | ZMMU RYA 1409 | KBPZM113-07 | GQ482413 |
| *Phylloscopus borealoides* | ZMMU RYA 1253 | KBPZM105-07 | GQ482414 |
| *Phylloscopus borealoides* | ZMMU RYA 1308 | KBPZM110-07 | GQ482415 |
| *Phylloscopus collybita* | UWBM 49384 | KBPBR094-07 | GQ482416 |
| *Phylloscopus collybita* | UWBM 49768 | KBPBR130-07 | GQ482417 |
| *Phylloscopus collybita* | UWBM 64865 | KBPBR479-07 | GQ482418 |
| *Phylloscopus collybita* | ZMMU 19a | KBPZM007-06 | GQ482419 |
| *Phylloscopus coronatus* | UWBM 47429 | KBPBR503-07 | GQ482420 |
| *Phylloscopus coronatus* | UWBM 74909 | KBPBR361-07 | GQ482421 |
| *Phylloscopus coronatus* | UWBM 72043 | KBPBR288-07 | GQ482422 |
| *Phylloscopus coronatus* | UWBM 72206 | KBPBR490-07 | GQ482423 |
| *Phylloscopus coronatus* | UWBM 47430 | KBPBR504-07 | GQ482424 |
| *Phylloscopus fuscatus* | UWBM 59684 | KBPBR145-07 | GQ482425 |
| *Phylloscopus fuscatus* | UWBM 73411 | KBPBR344-07 | GQ482426 |
| *Phylloscopus fuscatus* | UWBM 44437 | KBPBR382-07 | GQ482427 |
| *Phylloscopus fuscatus* | UWBM 47301 | KBPBR392-07 | GQ482428 |
| *Phylloscopus fuscatus* | UWBM 60164 | KBPBR229-07 | GQ482429 |
| *Phylloscopus fuscatus* | UWBM 46566 | KBPBR254-07 | GQ482430 |
| *Phylloscopus fuscatus* | UWBM 78425 | KBPBR358-07 | GQ482431 |
| *Phylloscopus fuscatus* | UWBM 43942 | KBPBR156-07 | GQ482432 |
| *Phylloscopus griseolus* | UWBM 66478 | KBPBR334-07 | GQ482433 |
| *Phylloscopus humei* | UWBM 51811 | KBPBR427-07 | GQ482434 |
| *Phylloscopus humei* | UWBM 58028 | KBPBR219-07 | GQ482435 |
| *Phylloscopus humei* | UWBM 46480 | KBPBR243-07 | GQ482436 |
| *Phylloscopus inornatus* | UWBM 52519 | KBPBR411-07 | GQ482437 |
| *Phylloscopus inornatus* | UWBM 51149 | KBPBR168-07 | GQ482438 |
| *Phylloscopus inornatus* | UWBM 82283 | KBPBR497-07 | GQ482439 |
| *Phylloscopus proregulus* | UWBM 59689 | KBPBR146-07 | GQ482440 |
| *Phylloscopus proregulus* | UWBM 58444 | KBPBR528-07 | GQ482441 |
| *Phylloscopus proregulus* | UWBM 59743 | KBPBR149-07 | GQ482442 |
| *Phylloscopus proregulus* | UWBM 78363 | KBPBR354-07 | GQ482443 |
| *Phylloscopus proregulus* | UWBM 52538 | KBPBR413-07 | GQ482444 |
| *Phylloscopus proregulus* | UWBM 74870 | KBPBR531-07 | GQ482445 |
| *Phylloscopus proregulus* | UWBM 47589 | KBPBR520-07 | GQ482446 |
| *Phylloscopus sibilatrix* | UWBM 49281 | KBPBR082-07 | GQ482447 |
| *Phylloscopus sibilatrix* | UWBM 49634 | KBPBR122-07 | GQ482448 |
| *Phylloscopus sibilatrix* | UWBM 61412 | KBPBR449-07 | GQ482449 |
| *Phylloscopus tenellipes* | UWBM 72179 | KBPBR488-07 | GQ482450 |
| *Phylloscopus tenellipes* | UWBM 74736 | KBPBR398-07 | GQ482451 |
| *Phylloscopus tenellipes* | UWBM 59857 | KBPBR182-07 | GQ482452 |
| *Phylloscopus tenellipes* | UWBM 58455 | KBPBR530-07 | GQ482453 |
| *Phylloscopus trochiloides* | UWBM 78250 | KBPBR232-07 | GQ482454 |
| *Phylloscopus trochiloides* | UWBM 58029 | KBPBR220-07 | GQ482455 |
| *Phylloscopus trochiloides* | UWBM 46600 | KBPBR260-07 | GQ482456 |
| *Phylloscopus trochiloides* | UWBM 66640 | KBPBR481-07 | GQ482457 |
| *Phylloscopus trochiloides* | UWBM 73594 | KBPBR509-07 | GQ482458 |
| *Phylloscopus trochiloides* | UWBM 46413 | KBPBR204-07 | GQ482459 |
| *Phylloscopus trochiloides* | UWBM 49473 | KBPBR107-07 | GQ482460 |
| *Phylloscopus trochiloides* | UWBM 60108 | KBPBR227-07 | GQ482461 |
| *Phylloscopus trochilus* | UWBM 44238 | KBPBR285-07 | GQ482462 |
| *Phylloscopus trochilus* | UWBM 56552 | KBPBR139-07 | GQ482463 |
| *Phylloscopus trochilus* | UWBM 49727 | KBPBR126-07 | GQ482464 |
| *Phylloscopus trochilus* | UWBM 59658 | KBPBR440-07 | GQ482465 |
| *Phylloscopus trochilus* | ZMMU 17a | KBPZM005-06 | GQ482466 |
| *Phylloscopus trochilus* | UWBM 59650 | KBPBR438-07 | GQ482467 |
| *Phylloscopus trochilus* | UWBM 61378 | KBPBR448-07 | GQ482468 |
| *Pica pica* | UWBM 61362 | KBPBK302-08 | GQ482469 |
| *Pica pica* | UWBM 56913 | KBPBK293-08 | GQ482470 |
| *Pica pica* | UWBM 59757 | KBPBK056-08 | GQ482471 |
| *Pica pica* | UWBM 72091 | KBPBK324-08 | GQ482472 |
| *Pica pica* | UWBM 58043 | KBPBK120-08 | GQ482473 |
| *Pica pica* | UWBM 66697 | KBPBK319-08 | GQ482474 |
| *Pica pica* | UWBM 60996 | KBPBK158-08 | GQ482475 |
| *Pica pica* | UWBM 73800 | KBPBK347-08 | GQ482476 |
| *Pica pica* | UWBM 69718 | KBPBK027-08 | GQ482477 |
| *Pica pica* | UWBM 44585 | KBPBK256-08 | GQ482478 |
| *Picoides tridactylus* | UWBM 56771 | KBPBU464-06 | GQ482479 |
| *Picoides tridactylus* | UWBM 49797 | KBPBU465-06 | GQ482480 |
| *Picoides tridactylus* | UWBM 51851 | KBPBU461-06 | GQ482481 |
| *Picoides tridactylus* | UWBM 47396 | KBPBU463-06 | GQ482482 |
| *Picoides tridactylus* | UWBM 51748 | KBPBU462-06 | GQ482483 |
| *Picus canus* | UWBM 47166 | KBPBU453-06 | GQ482484 |
| *Picus canus* | UWBM 72085 | KBPBU455-06 | GQ482485 |
| *Picus canus* | UWBM 51783 | KBPBU454-06 | GQ482486 |
| *Picus viridis* | UWBM 49638 | KBPBU460-06 | GQ482487 |
| *Picus viridis* | UWBM 49637 | KBPBU459-06 | GQ482488 |
| *Picus viridis* | UWBM 61128 | KBPBU457-06 | GQ482489 |
| *Picus viridis* | UWBM 61411 | KBPBU458-06 | GQ482490 |
| *Pinicola enucleator* | UWBM 82324 | KBPBK338-08 | GQ482491 |
| *Pinicola enucleator* | UWBM 56795 | KBPBK093-08 | GQ482492 |
| *Pinicola enucleator* | UWBM 44628 | KBPBK259-08 | GQ482493 |
| *Pinicola enucleator* | UWBM 47313 | KBPBK266-08 | GQ482494 |
| *Pinicola enucleator* | UWBM 51642 | KBPBK280-08 | GQ482495 |
| *Pinicola enucleator* | UWBM 51847 | KBPBK286-08 | GQ482496 |
| *Pinicola enucleator* | UWBM 46574 | KBPBK148-08 | GQ482497 |
| *Pinicola enucleator* | ZMMU RYA 1699a | KBPZM168-07 | GQ482498 |
| *Pinicola enucleator* | ZMMU RYA 1700 | KBPZM170-07 | GQ482499 |
| *Pinicola enucleator* | ZMMU RYA 1696 | KBPZM165-07 | GQ482500 |
| *Pinicola enucleator* | ZMMU RYA 1697 | KBPZM166-07 | GQ482501 |
| *Pinicola enucleator* | ZMMU RYA 1698 | KBPZM167-07 | GQ482502 |
| *Plectrophenax nivalis* | UWBM 82268 | KBPBK332-08 | GQ482503 |
| *Pluvialis apricaria* | UWBM 59599 | KBPBU171-06 | GQ482504 |
| *Pluvialis apricaria* | UWBM 49669 | KBPBU168-06 | GQ482505 |
| *Pluvialis apricaria* | UWBM 61323 | KBPBU170-06 | GQ482506 |
| *Pluvialis apricaria* | UWBM 49670 | KBPBU169-06 | GQ482507 |
| *Pluvialis fulva* | UWBM 44172 | KBPBU166-06 | GQ482508 |
| *Pluvialis fulva* | UWBM 44171 | KBPBU165-06 | GQ482509 |
| *Pluvialis fulva* | UWBM 44231 | KBPBU167-06 | GQ482510 |
| *Pluvialis squatarola* | UWBM 43964 | KBPBU162-06 | GQ482511 |
| *Pluvialis squatarola* | UWBM 44500 | KBPBU163-06 | GQ482512 |
| *Pluvialis squatarola* | UWBM 51608 | KBPBU164-06 | GQ482513 |
| *Pluvialis squatarola* | UWBM 43963 | KBPBU161-06 | GQ482514 |
| *Podiceps cristatus* | UWBM 56440 | KBPBU003-06 | GQ482515 |
| *Podiceps cristatus* | UWBM 46892 | KBPBU004-06 | GQ482516 |
| *Podiceps cristatus* | UWBM 73383 | KBPBU005-06 | GQ482517 |
| *Podiceps cristatus* | UWBM 47186 | KBPBU006-06 | GQ482518 |
| *Podiceps nigricollis* | UWBM 73789 | KBPBU009-06 | GQ482519 |
| *Podiceps nigricollis* | UWBM 73209 | KBPBU008-06 | GQ482520 |
| *Podoces hendersoni* | UWBM 57981 | KBPBK116-08 | GQ482521 |
| *Podoces hendersoni* | UWBM 57850 | KBPBK048-08 | GQ482522 |
| *Poecile cincta* | UWBM 46570 | KBPBR256-07 | GQ482523 |
| *Poecile cincta* | UWBM 56944 | KBPBR435-07 | GQ482524 |
| *Poecile cincta* | UWBM 49742 | KBPBR127-07 | GQ482525 |
| *Poecile cincta* | UWBM 78419 | KBPBR357-07 | GQ482526 |
| *Poecile montana* | UWBM 49453 | KBPBR103-07 | GQ482527 |
| *Poecile montana* | UWBM 59749 | KBPBR150-07 | GQ482528 |
| *Poecile montana* | UWBM 44148 | KBPBR197-07 | GQ482529 |
| *Poecile montana* | UWBM 74183 | KBPBR313-07 | GQ482530 |
| *Poecile montana* | UWBM 49720 | KBPBR125-07 | GQ482531 |
| *Poecile montana* | UWBM 49619 | KBPBR121-07 | GQ482532 |
| *Poecile montana* | UWBM 73494 | KBPBR350-07 | GQ482533 |
| *Poecile montana* | UWBM 52561 | KBPBR415-07 | GQ482534 |
| *Poecile montana* | UWBM 78280 | KBPBR233-07 | GQ482535 |
| *Poecile montana* | UWBM 80557 | KBPBR241-07 | GQ482536 |
| *Poecile montana* | UWBM 46568 | KBPBR255-07 | GQ482537 |
| *Poecile montana* | UWBM 82991 | KBPBR374-07 | GQ482538 |
| *Poecile montana* | UWBM 82298 | KBPBR500-07 | GQ482539 |
| *Poecile montana* | UWBM 58424 | KBPBR526-07 | GQ482540 |
| *Poecile montana* | UWBM 56718 | KBPBR180-07 | GQ482541 |
| *Poecile montana* | UWBM 58013 | KBPBR217-07 | GQ482542 |
| *Poecile montana* | ZMMU RYA 511 | KBPZM252-07 | GQ482543 |
| *Poecile montana* | ZMMU RYA 1042 | KBPZM087-07 | GQ482544 |
| *Poecile montana* | ZMMU RYA 1212 | KBPZM102-07 | GQ482545 |
| *Poecile montana* | ZMMU RYA 429 | KBPZM250-07 | GQ482546 |
| *Poecile palustris* | UWBM 78334 | KBPBR353-07 | GQ482547 |
| *Poecile palustris* | UWBM 75300 | KBPBR366-07 | GQ482548 |
| *Poecile palustris* | UWBM 74604 | KBPBR264-07 | GQ482549 |
| *Poecile palustris* | UWBM 73626 | KBPBR510-07 | GQ482550 |
| *Poecile palustris* | UWBM 47492 | KBPBR507-07 | GQ482551 |
| *Poecile palustris* | ZMMU RYA 1717 | KBPZM181-07 | GQ482552 |
| *Poecile palustris* | ZMMU RYA 308 | KBPZM248-07 | GQ482553 |
| *Poecile palustris* | ZMMU RYA 482 | KBPZM251-07 | GQ482554 |
| *Poecile palustris* | ZMMU 72a | KBPZM037-06 | GQ482555 |
| *Poecile palustris* | ZMMU 73a | KBPZM038-06 | GQ482556 |
| *Poecile palustris* | ZMMU RYA 1065 | KBPZM093-07 | GQ482557 |
| *Porzana porzana* | UWBM 60982 | KBPBU130-06 | GQ482558 |
| *Prunella atrogularis* | UWBM 46573 | KBPBR258-07 | GQ482559 |
| *Prunella atrogularis* | UWBM 46572 | KBPBR257-07 | GQ482560 |
| *Prunella fulvescens* | UWBM 57988 | KBPBR214-07 | GQ482561 |
| *Prunella fulvescens* | UWBM 58007 | KBPBR215-07 | GQ482562 |
| *Prunella fulvescens* | UWBM 46421 | KBPBR205-07 | GQ482563 |
| *Prunella himalayana* | UWBM 67647 | KBPBR297-07 | GQ482564 |
| *Prunella himalayana* | UWBM 46423 | KBPBR206-07 | GQ482565 |
| *Prunella montanella* | UWBM 47361 | KBPBR416-07 | GQ482566 |
| *Prunella montanella* | UWBM 44004 | KBPBR158-07 | GQ482567 |
| *Prunella rubida* | ZMMU RYA 1708 | KBPZM175-07 | GQ482568 |
| *Ptyonoprogne rupestris* | UWBM 66323 | KBPBU560-06 | GQ482569 |
| *Ptyonoprogne rupestris* | UWBM 66658 | KBPBU561-06 | GQ482570 |
| *Pyrrhocorax graculus* | UWBM 61472 | KBPBK307-08 | GQ482571 |
| *Pyrrhocorax graculus* | UWBM 61471 | KBPBK306-08 | GQ482572 |
| *Pyrrhocorax pyrrhocorax* | UWBM 59742 | KBPBK060-08 | GQ482573 |
| *Pyrrhocorax pyrrhocorax* | UWBM 75747 | KBPBK188-08 | GQ482574 |
| *Pyrrhocorax pyrrhocorax* | UWBM 58057 | KBPBK122-08 | GQ482575 |
| *Pyrrhocorax pyrrhocorax* | UWBM 46418 | KBPBK111-08 | GQ482576 |
| *Pyrrhula pyrrhula* | UWBM 44038 | KBPBK078-08 | GQ482577 |
| *Pyrrhula pyrrhula* | UWBM 73660 | KBPBK343-08 | GQ482578 |
| *Pyrrhula pyrrhula* | UWBM 51637 | KBPBK279-08 | GQ482579 |
| *Pyrrhula pyrrhula* | UWBM 49773 | KBPBK038-08 | GQ482580 |
| *Pyrrhula pyrrhula* | UWBM 56768 | KBPBK091-08 | GQ482581 |
| *Pyrrhula pyrrhula* | UWBM 74862 | KBPBK357-08 | GQ482582 |
| *Pyrrhula pyrrhula* | UWBM 47593 | KBPBK348-08 | GQ482583 |
| *Pyrrhula pyrrhula* | UWBM 74319 | KBPBK201-08 | GQ482584 |
| *Pyrrhula pyrrhula* | UWBM 66529 | KBPBK219-08 | GQ482585 |
| *Pyrrhula pyrrhula* | UWBM 78212 | KBPBK137-08 | GQ482586 |
| *Pyrrhula pyrrhula* | UWBM 64790 | KBPBK316-08 | GQ482587 |
| *Pyrrhula pyrrhula* | ZMMU RYA 1687 | KBPZM159-07 | GQ482588 |
| *Pyrrhula pyrrhula* | ZMMU RYA 1699b | KBPZM169-07 | GQ482589 |
| *Pyrrhula pyrrhula* | ZMMU RYA 1676 | KBPZM150-07 | GQ482590 |
| *Pyrrhula pyrrhula* | ZMMU RYA 1677 | KBPZM151-07 | GQ482591 |
| *Pyrrhula pyrrhula* | ZMMU RYA 1686 | KBPZM158-07 | GQ482592 |
| *Rallus aquaticus* | UWBM 46943 | KBPBU129-06 | GQ482593 |
| *Regulus regulus* | UWBM 47210 | KBPBR387-07 | GQ482594 |
| *Regulus regulus* | UWBM 47377 | KBPBR418-07 | GQ482595 |
| *Regulus regulus* | UWBM 56962 | KBPBR436-07 | GQ482596 |
| *Regulus regulus* | UWBM 57233 | KBPBR522-07 | GQ482597 |
| *Regulus regulus* | UWBM 74680 | KBPBR394-07 | GQ482598 |
| *Regulus regulus* | UWBM 64690 | KBPBR465-07 | GQ482599 |
| *Regulus regulus* | ZMMU RYA 1790 | KBPZM210-07 | GQ482600 |
| *Regulus regulus* | ZMMU RYA 1789 | KBPZM209-07 | GQ482601 |
| *Regulus regulus* | ZMMU RYA 1791 | KBPZM211-07 | GQ482602 |
| *Regulus regulus* | ZMMU RYA 1788 | KBPZM208-07 | GQ482603 |
| *Rhodopechys mongolica* | UWBM 46256 | KBPBK065-08 | GQ482604 |
| *Rhodopechys mongolica* | UWBM 46248 | KBPBK064-08 | GQ482605 |
| *Rhodopechys mongolica* | UWBM 75871 | KBPBK192-08 | GQ482606 |
| *Rhodopechys mongolica* | UWBM 57955 | KBPBK114-08 | GQ482607 |
| *Rhodopechys mongolica* | UWBM 57968 | KBPBK115-08 | GQ482608 |
| *Riparia diluta* | UWBM 67556 | KBPBU557-06 | GQ482609 |
| *Riparia riparia* | ZMMU RYA 1056 | KBPZM091-07 | GQ482610 |
| *Riparia riparia* | ZMMU RYA 1055 | KBPZM090-07 | GQ482611 |
| *Riparia riparia* | ZMMU RYA 1053 | KBPZM089-07 | GQ482612 |
| *Riparia riparia* | UWBM 59972 | KBPBU555-06 | GQ482613 |
| *Riparia riparia* | ZMMU RYA 1052 | KBPZM088-07 | GQ482614 |
| *Riparia riparia* | UWBM 49641 | KBPBU556-06 | GQ482615 |
| *Riparia riparia* | UWBM 46945 | KBPBU558-06 | GQ482616 |
| *Riparia riparia* | UWBM 59627 | KBPBU559-06 | GQ482617 |
| *Saxicola insignis* | UWBM 57885 | KBPBU720-06 | GQ482618 |
| *Saxicola insignis* | UWBM 58004 | KBPBU721-06 | GQ482619 |
| *Saxicola maurus* | UWBM 47121 | KBPBU725-06 | GQ482620 |
| *Saxicola maurus* | UWBM 51190 | KBPBU726-06 | GQ482621 |
| *Saxicola maurus* | UWBM 46554 | KBPBU722-06 | GQ482622 |
| *Saxicola maurus* | UWBM 59770 | KBPBU723-06 | GQ482623 |
| *Saxicola maurus* | ZMMU RYA 1794 | KBPZM212-07 | GQ482624 |
| *Saxicola maurus* | ZMMU RYA 1795 | KBPZM213-07 | GQ482625 |
| *Saxicola maurus* | ZMMU RYA 1739 | KBPZM191-07 | GQ482626 |
| *Saxicola maurus* | ZMMU RYA 1609 | KBPZM134-07 | GQ482627 |
| *Saxicola rubetra* | UWBM 49183 | KBPBU717-06 | GQ482628 |
| *Saxicola rubetra* | UWBM 64800 | KBPBU715-06 | GQ482629 |
| *Saxicola rubetra* | UWBM 49340 | KBPBU716-06 | GQ482630 |
| *Saxicola rubetra* | UWBM 59665 | KBPBU718-06 | GQ482631 |
| *Saxicola rubetra* | UWBM 49561 | KBPBU719-06 | GQ482632 |
| *Saxicola rubicola* | UWBM 61437 | KBPBU724-06 | GQ482633 |
| *Scolopax rusticola* | UWBM 47395 | KBPBU287-06 | GQ482634 |
| *Scolopax rusticola* | UWBM 61577 | KBPBU289-06 | GQ482635 |
| *Scolopax rusticola* | UWBM 51816 | KBPBU288-06 | GQ482636 |
| *Scolopax rusticola* | UWBM 61000 | KBPBU286-06 | GQ482637 |
| *Scolopax rusticola* | UWBM 46931 | KBPBU285-06 | GQ482638 |
| *Serinus pusillus* | UWBM 64761 | KBPBK314-08 | GQ482639 |
| *Serinus pusillus* | UWBM 61193 | KBPBK163-08 | GQ482640 |
| *Serinus pusillus* | UWBM 61499 | KBPBK309-08 | GQ482641 |
| *Sitta europaea* | UWBM 47255 | KBPBR389-07 | GQ482642 |
| *Sitta europaea* | UWBM 64712 | KBPBR471-07 | GQ482643 |
| *Sitta europaea* | UWBM 44041 | KBPBR159-07 | GQ482644 |
| *Sitta europaea* | UWBM 49426 | KBPBR100-07 | GQ482645 |
| *Sitta europaea* | UWBM 82319 | KBPBR501-07 | GQ482646 |
| *Sitta europaea* | UWBM 46868 | KBPBR074-07 | GQ482647 |
| *Sitta europaea* | UWBM 57910 | KBPBR143-07 | GQ482648 |
| *Sitta europaea* | UWBM 56787 | KBPBR181-07 | GQ482649 |
| *Sitta europaea* | UWBM 78281 | KBPBR234-07 | GQ482650 |
| *Sitta europaea* | UWBM 66707 | KBPBR482-07 | GQ482651 |
| *Sitta europaea* | ZMMU RYA 1600 | KBPZM130-07 | GQ482652 |
| *Sitta europaea* | ZMMU RYA 1718 | KBPZM182-07 | GQ482653 |
| *Sitta europaea* | ZMMU RYA 1573 | KBPZM121-07 | GQ482654 |
| *Sitta krueperi* | UWBM 64809 | KBPBR477-07 | GQ482655 |
| *Sitta krueperi* | UWBM 61508 | KBPBR458-07 | GQ482656 |
| *Sitta krueperi* | UWBM 61527 | KBPBR459-07 | GQ482657 |
| *Sitta krueperi* | UWBM 64691 | KBPBR466-07 | GQ482658 |
| *Somateria spectabilis* | UWBM 43965 | KBPBU062-06 | GQ482659 |
| *Stercorarius longicaudus* | UWBM 43888 | KBPBU294-06 | GQ482660 |
| *Stercorarius longicaudus* | UWBM 43950 | KBPBU295-06 | GQ482661 |
| *Stercorarius parasiticus* | UWBM 43914 | KBPBU299-06 | GQ482662 |
| *Sterna hirundo* | UWBM 59703 | KBPBU345-06 | GQ482663 |
| *Sterna hirundo* | UWBM 61055 | KBPBU343-06 | GQ482664 |
| *Sterna hirundo* | UWBM 44308 | KBPBU342-06 | GQ482665 |
| *Sterna hirundo* | UWBM 56582 | KBPBU341-06 | GQ482666 |
| *Sterna paradisaea* | UWBM 59528 | KBPBU350-06 | GQ482667 |
| *Sterna paradisaea* | UWBM 49683 | KBPBU347-06 | GQ482668 |
| *Sterna paradisaea* | UWBM 49713 | KBPBU348-06 | GQ482669 |
| *Sternula albifrons* | UWBM 61049 | KBPBU339-06 | GQ482670 |
| *Sternula albifrons* | UWBM 61048 | KBPBU338-06 | GQ482671 |
| *Streptopelia orientalis* | UWBM 46932 | KBPBU380-06 | GQ482672 |
| *Streptopelia orientalis* | UWBM 51729 | KBPBU378-06 | GQ482673 |
| *Streptopelia orientalis* | UWBM 61085 | KBPBU379-06 | GQ482674 |
| *Streptopelia orientalis* | UWBM 47283 | KBPBU381-06 | GQ482675 |
| *Streptopelia orientalis* | UWBM 75265 | KBPBU765-06 | GQ482676 |
| *Streptopelia orientalis* | UWBM 75076 | KBPBU764-06 | GQ482677 |
| *Streptopelia orientalis* | UWBM 75279 | KBPBU766-06 | GQ482678 |
| *Streptopelia turtur* | UWBM 61364 | KBPBU377-06 | GQ482679 |
| *Streptopelia turtur* | UWBM 46395 | KBPBU375-06 | GQ482680 |
| *Strix uralensis* | UWBM 51708 | KBPBU408-06 | GQ482681 |
| *Strix uralensis* | UWBM 57798 | KBPBU407-06 | GQ482682 |
| *Sturnia philippensis* | ZMMU EAK 105 | KBPZM078-07 | GQ482683 |
| *Sturnia philippensis* | ZMMU EAK 106 | KBPZM079-07 | GQ482684 |
| *Sturnia philippensis* | ZMMU RYA 1588 | KBPZM124-07 | GQ482685 |
| *Sturnia philippensis* | ZMMU RYA 1589 | KBPZM125-07 | GQ482686 |
| *Sturnia philippensis* | ZMMU EAK 104 | KBPZM077-07 | GQ482687 |
| *Sturnus cineraceus* | UWBM 46897 | KBPBK007-08 | GQ482688 |
| *Sturnus cineraceus* | UWBM 74720 | KBPBK268-08 | GQ482689 |
| *Sturnus cineraceus* | UWBM 59932 | KBPBK100-08 | GQ482690 |
| *Sturnus cineraceus* | UWBM 74799 | KBPBK270-08 | GQ482691 |
| *Sturnus cineraceus* | UWBM 59925 | KBPBK099-08 | GQ482692 |
| *Sturnus roseus* | UWBM 46227 | KBPBK062-08 | GQ482693 |
| *Sturnus roseus* | UWBM 46226 | KBPBK061-08 | GQ482694 |
| *Sturnus vulgaris* | UWBM 46261 | KBPBK066-08 | GQ482695 |
| *Sturnus vulgaris* | UWBM 56689 | KBPBK089-08 | GQ482696 |
| *Sturnus vulgaris* | UWBM 66736 | KBPBK321-08 | GQ482697 |
| *Sturnus vulgaris* | UWBM 49327 | KBPBK015-08 | GQ482698 |
| *Sturnus vulgaris* | UWBM 49517 | KBPBK024-08 | GQ482699 |
| *Sturnus vulgaris* | UWBM 74273 | KBPBK195-08 | GQ482700 |
| *Sylvia atricapilla* | UWBM 49651 | KBPBR124-07 | GQ482701 |
| *Sylvia atricapilla* | UWBM 49321 | KBPBR085-07 | GQ482702 |
| *Sylvia atricapilla* | UWBM 60972 | KBPBR267-07 | GQ482703 |
| *Sylvia atricapilla* | ZMMU 16a | KBPZM004-06 | GQ482704 |
| *Sylvia atricapilla* | ZMMU 24a | KBPZM012-06 | GQ482705 |
| *Sylvia atricapilla* | ZMMU 65a | KBPZM032-06 | GQ482706 |
| *Sylvia atricapilla* | ZMMU 15a | KBPZM003-06 | GQ482707 |
| *Sylvia atricapilla* | UWBM 64624 | KBPBR462-07 | GQ482708 |
| *Sylvia atricapilla* | UWBM 49510 | KBPBR114-07 | GQ482709 |
| *Sylvia borin* | UWBM 49379 | KBPBR092-07 | GQ482710 |
| *Sylvia borin* | UWBM 49470 | KBPBR105-07 | GQ482711 |
| *Sylvia borin* | UWBM 61023 | KBPBR269-07 | GQ482712 |
| *Sylvia borin* | UWBM 61376 | KBPBR446-07 | GQ482713 |
| *Sylvia communis* | UWBM 61338 | KBPBR444-07 | GQ482714 |
| *Sylvia communis* | UWBM 49444 | KBPBR101-07 | GQ482715 |
| *Sylvia communis* | UWBM 49249 | KBPBR080-07 | GQ482716 |
| *Sylvia communis* | UWBM 46535 | KBPBR252-07 | GQ482717 |
| *Sylvia communis* | UWBM 46447 | KBPBR207-07 | GQ482718 |
| *Sylvia communis* | UWBM 66619 | KBPBR480-07 | GQ482719 |
| *Sylvia communis* | ZMMU RYA 1923 | KBPZM236-07 | GQ482720 |
| *Sylvia curruca* | UWBM 56548 | KBPBR138-07 | GQ482721 |
| *Sylvia curruca* | UWBM 49357 | KBPBR090-07 | GQ482722 |
| *Sylvia curruca* | UWBM 59991 | KBPBR191-07 | GQ482723 |
| *Sylvia curruca* | UWBM 58018 | KBPBR218-07 | GQ482724 |
| *Sylvia curruca* | UWBM 46475 | KBPBR242-07 | GQ482725 |
| *Sylvia curruca* | UWBM 73729 | KBPBR511-07 | GQ482726 |
| *Sylvia curruca* | UWBM 49486 | KBPBR111-07 | GQ482727 |
| *Sylvia curruca* | ZMMU 119a | KBPZM051-06 | GQ482728 |
| *Sylvia curruca* | ZMMU 22a | KBPZM010-06 | GQ482729 |
| *Sylvia mystacea* | UWBM 56588 | KBPBR169-07 | GQ482730 |
| *Sylvia nana* | UWBM 57866 | KBPBR142-07 | GQ482731 |
| *Sylvia nana* | UWBM 57977 | KBPBR213-07 | GQ482732 |
| *Sylvia nisoria* | UWBM 46268 | KBPBR152-07 | GQ482733 |
| *Sylvia nisoria* | UWBM 49324 | KBPBR087-07 | GQ482734 |
| *Sylvia nisoria* | UWBM 49344 | KBPBR088-07 | GQ482735 |
| *Sylvia nisoria* | UWBM 46379 | KBPBR199-07 | GQ482736 |
| *Synthliboramphus antiquus* | UWBM 74989 | KBPBU763-06 | GQ482737 |
| *Syrrhaptes paradoxus* | UWBM 59840 | KBPBU361-06 | GQ482738 |
| *Syrrhaptes paradoxus* | UWBM 57962 | KBPBU362-06 | GQ482739 |
| *Syrrhaptes paradoxus* | UWBM 67598 | KBPBU363-06 | GQ482740 |
| *Syrrhaptes paradoxus* | UWBM 59826 | KBPBU360-06 | GQ482741 |
| *Tachybaptus ruficollis* | UWBM 61474 | KBPBU010-06 | GQ482742 |
| *Tachymarptis melba* | UWBM 61222 | KBPBU421-06 | GQ482743 |
| *Tachymarptis melba* | UWBM 61463 | KBPBU422-06 | GQ482744 |
| *Tadorna ferruginea* | UWBM 57893 | KBPBU027-06 | GQ482745 |
| *Tadorna ferruginea* | UWBM 56475 | KBPBU025-06 | GQ482746 |
| *Tadorna ferruginea* | UWBM 57887 | KBPBU026-06 | GQ482747 |
| *Tadorna ferruginea* | UWBM 56474 | KBPBU024-06 | GQ482748 |
| *Tadorna ferruginea* | UWBM 46399 | KBPBU028-06 | GQ482749 |
| *Tadorna tadorna* | UWBM 56439 | KBPBU032-06 | GQ482750 |
| *Tadorna tadorna* | UWBM 60091 | KBPBU030-06 | GQ482751 |
| *Tadorna tadorna* | UWBM 46270 | KBPBU029-06 | GQ482752 |
| *Tadorna tadorna* | UWBM 61349 | KBPBU033-06 | GQ482753 |
| *Tarsiger cyanurus* | UWBM 58015 | KBPBU727-06 | GQ482754 |
| *Tarsiger cyanurus* | UWBM 51558 | KBPBU730-06 | GQ482755 |
| *Tarsiger cyanurus* | UWBM 47111 | KBPBU729-06 | GQ482756 |
| *Tarsiger cyanurus* | UWBM 56725 | KBPBU731-06 | GQ482757 |
| *Tarsiger cyanurus* | UWBM 59701 | KBPBU728-06 | GQ482758 |
| *Terpsiphone paradisi* | UWBM 72122 | KBPBR486-07 | GQ482759 |
| *Tetraogallus altaicus* | UWBM 75676 | KBPBU114-06 | GQ482760 |
| *Tetrao parvirostris* | UWBM 51609 | KBPBU103-06 | GQ482761 |
| *Tetrao parvirostris* | UWBM 44017 | KBPBU100-06 | GQ482762 |
| *Tetrao parvirostris* | UWBM 44311 | KBPBU101-06 | GQ482763 |
| *Tetrao parvirostris* | UWBM 51649 | KBPBU104-06 | GQ482764 |
| *Tetrao parvirostris* | UWBM 47363 | KBPBU102-06 | GQ482765 |
| *Tetrao tetrix* | UWBM 61547 | KBPBU096-06 | GQ482766 |
| *Tetrao tetrix* | UWBM 63815 | KBPBU098-06 | GQ482767 |
| *Tetrao tetrix* | UWBM 61555 | KBPBU097-06 | GQ482768 |
| *Tetrao tetrix* | UWBM 64872 | KBPBU099-06 | GQ482769 |
| *Tetrao tetrix* | UWBM 57293 | KBPBU095-06 | GQ482770 |
| *Tetrao urogallus* | UWBM 49746 | KBPBU106-06 | GQ482771 |
| *Tetrao urogallus* | UWBM 66698 | KBPBU108-06 | GQ482772 |
| *Tetrao urogallus* | UWBM 61546 | KBPBU107-06 | GQ482773 |
| *Tetrax tetrax* | UWBM 64702 | KBPBU128-06 | GQ482774 |
| *Thalasseus sandvicensis* | UWBM 61054 | KKBNA533-05 | GQ482775 |
| *Thalasseus sandvicensis* | UWBM 61053 | KKBNA532-05 | GQ482776 |
| *Tichodroma muraria* | UWBM 61504 | KBPBR456-07 | GQ482777 |
| *Tringa brevipes* | UWBM 44448 | KBPBU254-06 | GQ482778 |
| *Tringa erythropus* | UWBM 44495 | KBPBU239-06 | GQ482779 |
| *Tringa erythropus* | UWBM 51066 | KBPBU236-06 | GQ482780 |
| *Tringa erythropus* | UWBM 51068 | KBPBU237-06 | GQ482781 |
| *Tringa erythropus* | UWBM 51069 | KBPBU238-06 | GQ482782 |
| *Tringa glareola* | UWBM 51134 | KBPBU258-06 | GQ482783 |
| *Tringa glareola* | UWBM 56441 | KBPBU256-06 | GQ482784 |
| *Tringa glareola* | UWBM 56513 | KBPBU257-06 | GQ482785 |
| *Tringa glareola* | UWBM 49734 | KBPBU255-06 | GQ482786 |
| *Tringa ochropus* | UWBM 49781 | KBPBU250-06 | GQ482787 |
| *Tringa ochropus* | UWBM 46903 | KBPBU251-06 | GQ482788 |
| *Tringa ochropus* | UWBM 49481 | KBPBU249-06 | GQ482789 |
| *Tringa ochropus* | UWBM 61210 | KBPBU252-06 | GQ482790 |
| *Tringa ochropus* | UWBM 44294 | KBPBU253-06 | GQ482791 |
| *Tringa stagnatilis* | UWBM 59721 | KBPBU247-06 | GQ482792 |
| *Tringa stagnatilis* | UWBM 66737 | KBPBU246-06 | GQ482793 |
| *Tringa stagnatilis* | UWBM 59950 | KBPBU248-06 | GQ482794 |
| *Tringa totanus* | UWBM 49714 | KBPBU240-06 | GQ482795 |
| *Tringa totanus* | UWBM 59800 | KBPBU244-06 | GQ482796 |
| *Tringa totanus* | UWBM 59942 | KBPBU245-06 | GQ482797 |
| *Tringa totanus* | UWBM 66352 | KBPBU242-06 | GQ482798 |
| *Tringa totanus* | UWBM 46266 | KBPBU243-06 | GQ482799 |
| *Tringa totanus* | UWBM 61033 | KBPBU241-06 | GQ482800 |
| *Troglodytes troglodytes* | ZMMU 58a | KBPZM026-06 | GQ482801 |
| *Troglodytes troglodytes* | ZMMU 21a | KBPZM009-06 | GQ482802 |
| *Troglodytes troglodytes* | UWBM 47389 | KKBNA729-05 | GQ482803 |
| *Troglodytes troglodytes* | ZMMU RYA 1555 | KBPZM117-07 | GQ482804 |
| *Troglodytes troglodytes* | ZMMU RYA 1562 | KBPZM118-07 | GQ482805 |
| *Troglodytes troglodytes* | ZMMU RYA 1704 | KBPZM171-07 | GQ482806 |
| *Troglodytes troglodytes* | ZMMU RYA 1841 | KBPZM224-07 | GQ482807 |
| *Troglodytes troglodytes* | UWBM 46951 | KBPBU642-06 | GQ482808 |
| *Troglodytes troglodytes* | UWBM 46602 | KBPBU639-06 | GQ482809 |
| *Troglodytes troglodytes* | UWBM 61118 | KBPBU640-06 | GQ482810 |
| *Troglodytes troglodytes* | UWBM 57291 | KBPBU641-06 | GQ482811 |
| *Troglodytes troglodytes* | UWBM 47536 | KKBNA747-05 | GQ482812 |
| *Turdus chrysolaus* | ZMMU RYA 1732 | KBPZM188-07 | GQ482813 |
| *Turdus chrysolaus* | ZMMU RYA 1745 | KBPZM194-07 | GQ482814 |
| *Turdus chrysolaus* | ZMMU RYA 1228 | KBPZM103-07 | GQ482815 |
| *Turdus chrysolaus* | ZMMU RYA 1674 | KBPZM148-07 | GQ482816 |
| *Turdus chrysolaus* | ZMMU RYA 1678 | KBPZM152-07 | GQ482817 |
| *Turdus chrysolaus* | ZMMU RYA 1679 | KBPZM153-07 | GQ482818 |
| *Turdus chrysolaus* | ZMMU RYA 1153 | KBPZM095-07 | GQ482819 |
| *Turdus chrysolaus* | UWBM 47575 | KBPBU643-06 | GQ482820 |
| *Turdus chrysolaus* | UWBM 47578 | KBPBU644-06 | GQ482821 |
| *Turdus hortulorum* | UWBM 75228 | KBPBU771-06 | GQ482822 |
| *Turdus hortulorum* | UWBM 75036 | KBPBU770-06 | GQ482823 |
| *Turdus iliacus* | UWBM 49795 | KBPBU647-06 | GQ482824 |
| *Turdus iliacus* | UWBM 49506 | KBPBU646-06 | GQ482825 |
| *Turdus iliacus* | UWBM 49430 | KBPBU645-06 | GQ482826 |
| *Turdus iliacus* | UWBM 59430 | KBPBU648-06 | GQ482827 |
| *Turdus iliacus* | UWBM 73166 | KBPBU784-06 | GQ482828 |
| *Turdus merula* | ZMMU 69a | KBPZM036-06 | GQ482829 |
| *Turdus merula* | ZMMU 23a | KBPZM011-06 | GQ482830 |
| *Turdus merula* | ZMMU 111a | KBPZM047-06 | GQ482831 |
| *Turdus merula* | ZMMU 26a | KBPZM014-06 | GQ482832 |
| *Turdus merula* | UWBM 49341 | KBPBU651-06 | GQ482833 |
| *Turdus merula* | UWBM 61574 | KBPBU650-06 | GQ482834 |
| *Turdus merula* | UWBM 64714 | KBPBU649-06 | GQ482835 |
| *Turdus merula* | UWBM 57257 | KBPBU653-06 | GQ482836 |
| *Turdus merula* | UWBM 49407 | KBPBU652-06 | GQ482837 |
| *Turdus naumanni* | ZMMU ASF 21 | KBPZM070-07 | GQ482838 |
| *Turdus naumanni* | ZMMU RYA 298 | KBPZM246-07 | GQ482839 |
| *Turdus naumanni* | ZMMU RYA 217 | KBPZM245-07 | GQ482840 |
| *Turdus naumanni* | ZMMU ASF 10 | KBPZM068-07 | GQ482841 |
| *Turdus naumanni* | UWBM 59771 | KBPBU654-06 | GQ482842 |
| *Turdus naumanni* | UWBM 44534 | KBPBU655-06 | GQ482843 |
| *Turdus naumanni* | UWBM 51892 | KBPBU656-06 | GQ482844 |
| *Turdus naumanni* | UWBM 74884 | KBPBU772-06 | GQ482845 |
| *Turdus naumanni* | UWBM 75006 | KBPBU773-06 | GQ482846 |
| *Turdus obscurus* | UWBM 59713 | KBPBU657-06 | GQ482847 |
| *Turdus obscurus* | UWBM 58395 | KBPBU661-06 | GQ482848 |
| *Turdus obscurus* | UWBM 57936 | KBPBU658-06 | GQ482849 |
| *Turdus obscurus* | UWBM 47113 | KBPBU660-06 | GQ482850 |
| *Turdus obscurus* | UWBM 44019 | KBPBU659-06 | GQ482851 |
| *Turdus pallidus* | UWBM 47446 | KBPBU662-06 | GQ482852 |
| *Turdus pallidus* | UWBM 74609 | KBPBU785-06 | GQ482853 |
| *Turdus pallidus* | UWBM 71759 | KBPBU665-06 | GQ482854 |
| *Turdus pallidus* | UWBM 47478 | KBPBU663-06 | GQ482855 |
| *Turdus philomelos* | UWBM 61127 | KBPBU667-06 | GQ482856 |
| *Turdus philomelos* | UWBM 66753 | KBPBU666-06 | GQ482857 |
| *Turdus philomelos* | UWBM 49794 | KBPBU669-06 | GQ482858 |
| *Turdus philomelos* | UWBM 49626 | KBPBU668-06 | GQ482859 |
| *Turdus pilaris* | UWBM 61654 | KBPBU670-06 | GQ482860 |
| *Turdus pilaris* | UWBM 49485 | KBPBU671-06 | GQ482861 |
| *Turdus pilaris* | UWBM 73340 | KBPBU787-06 | GQ482862 |
| *Turdus pilaris* | UWBM 59522 | KBPBU672-06 | GQ482863 |
| *Turdus ruficollis* | ZMMU 203\1999 | KBPZM061-07 | GQ482864 |
| *Turdus ruficollis* | ZMMU 223\1999 | KBPZM062-07 | GQ482865 |
| *Turdus ruficollis* | ZMMU 279\2000 | KBPZM063-07 | GQ482866 |
| *Turdus ruficollis* | UWBM 73524 | KBPBU788-06 | GQ482867 |
| *Turdus ruficollis* | UWBM 66709 | KBPBU675-06 | GQ482868 |
| *Turdus ruficollis* | UWBM 46282 | KBPBU674-06 | GQ482869 |
| *Turdus ruficollis* | UWBM 58023 | KBPBU673-06 | GQ482870 |
| *Turdus ruficollis* | UWBM 56711 | KBPBU676-06 | GQ482871 |
| *Turdus torquatus* | ZMMU EAK 065 | KBPZM072-07 | GQ482872 |
| *Turdus torquatus* | UWBM 64747 | KBPBU679-06 | GQ482873 |
| *Turdus torquatus* | UWBM 61195 | KBPBU677-06 | GQ482874 |
| *Turdus torquatus* | UWBM 61213 | KBPBU678-06 | GQ482875 |
| *Turdus viscivorus* | ZMMU RYA 1962 | KBPZM241-07 | GQ482876 |
| *Turdus viscivorus* | ZMMU 62\2000 | KBPZM067-07 | GQ482877 |
| *Turdus viscivorus* | ZMMU 61\2000 | KBPZM066-07 | GQ482878 |
| *Turdus viscivorus* | UWBM 57249 | KBPBU684-06 | GQ482879 |
| *Turdus viscivorus* | UWBM 61464 | KBPBU683-06 | GQ482880 |
| *Turdus viscivorus* | UWBM 61219 | KBPBU682-06 | GQ482881 |
| *Turdus viscivorus* | UWBM 46429 | KBPBU680-06 | GQ482882 |
| *Turdus viscivorus* | UWBM 51698 | KBPBU681-06 | GQ482883 |
| *Upupa epops* | UWBM 59860 | KBPBU492-06 | GQ482884 |
| *Upupa epops* | UWBM 46231 | KBPBU491-06 | GQ482885 |
| *Upupa epops* | UWBM 61068 | KBPBU493-06 | GQ482886 |
| *Upupa epops* | UWBM 49243 | KBPBU494-06 | GQ482887 |
| *Upupa epops* | UWBM 66321 | KBPBU495-06 | GQ482888 |
| *Uragus sibiricus* | ZMMU RYA 1692 | KBPZM164-07 | GQ482889 |
| *Uragus sibiricus* | ZMMU RYA 1765 | KBPZM197-07 | GQ482890 |
| *Uragus sibiricus* | ZMMU RYA 1815 | KBPZM221-07 | GQ482891 |
| *Uragus sibiricus* | ZMMU RYA 1766 | KBPZM198-07 | GQ482892 |
| *Uragus sibiricus* | ZMMU RYA 1767 | KBPZM199-07 | GQ482893 |
| *Uragus sibiricus* | UWBM 47075 | KBPBK082-08 | GQ482894 |
| *Uragus sibiricus* | UWBM 47303 | KBPBK264-08 | GQ482895 |
| *Uragus sibiricus* | UWBM 73755 | KBPBK346-08 | GQ482896 |
| *Uragus sibiricus* | UWBM 66320 | KBPBK207-08 | GQ482897 |
| *Uragus sibiricus* | UWBM 74818 | KBPBK271-08 | GQ482898 |
| *Uragus sibiricus* | UWBM 59876 | KBPBK098-08 | GQ482899 |
| *Uria lomvia* | UWBM 44177 | KBPBU355-06 | GQ482900 |
| *Urosphena squameiceps* | UWBM 74744 | KBPBR399-07 | GQ482901 |
| *Urosphena squameiceps* | UWBM 71692 | KBPBR238-07 | GQ482902 |
| *Urosphena squameiceps* | UWBM 72190 | KBPBR489-07 | GQ482903 |
| *Urosphena squameiceps* | UWBM 47107 | KBPBR165-07 | GQ482904 |
| *Urosphena squameiceps* | UWBM 47559 | KBPBR517-07 | GQ482905 |
| *Vanellus vanellus* | UWBM 46383 | KBPBU136-06 | GQ482906 |
| *Vanellus vanellus* | UWBM 46397 | KBPBU137-06 | GQ482907 |
| *Vanellus vanellus* | UWBM 49187 | KBPBU135-06 | GQ482908 |
| *Vanellus vanellus* | UWBM 59768 | KBPBU138-06 | GQ482909 |
| *Vanellus vanellus* | UWBM 60142 | KBPBU139-06 | GQ482910 |
| *Xenus cinereus* | UWBM 51167 | KBPBU265-06 | GQ482911 |
| *Xenus cinereus* | UWBM 44085 | KBPBU264-06 | GQ482912 |
| *Xenus cinereus* | UWBM 59959 | KBPBU268-06 | GQ482913 |
| *Xenus cinereus* | UWBM 61247 | KBPBU266-06 | GQ482914 |
| *Xenus cinereus* | UWBM 44536 | KBPBU267-06 | GQ482915 |
| *Zosterops erythropleurus* | UWBM 74789 | KBPBR402-07 | GQ482916 |
| *Zosterops erythropleurus* | UWBM 72044 | KBPBR289-07 | GQ482917 |
| *Zosterops erythropleurus* | UWBM 47437 | KBPBR505-07 | GQ482918 |
| *Zosterops erythropleurus* | UWBM 58440 | KBPBR527-07 | GQ482919 |
| *Zosterops erythropleurus* | UWBM 72103 | KBPBR485-07 | GQ482920 |
