# Supplementary material for: Filling the gap - COI barcode resolution in eastern Palearctic birds
Source: Front Zool. 2009 Dec 9;6:29. doi: 10.1186/1742-9994-6-29 (PMC2796652; doi:10.1186/1742-9994-6-29)
Supplement: Additional file 2 — List of sequences acquired from BOLD. Complete list of BOLD process identification numbers and GenBank accession numbers for all sequences used in this study from the "Birds of North America - Phase II" project in BOLD. [file 1742-9994-6-29-S2.DOC]

Table S2.

| **Species** | **Length (bp)** | **BOLD Process ID** | **GenBank Accession** |
| --- | --- | --- | --- |
| *Accipiter cooperii* | 644 | BOTW029-04 | DQ432694 |
| *Accipiter cooperii* | 692 | TZBNA241-03 | AY666285 |
| *Accipiter cooperii* | 653 | TZBNA288-03 | AY666504 |
| *Accipiter gentilis* | 615 | BOTW030-04 | DQ432695 |
| *Accipiter gentilis* | 668 | TZBNA297-03 | AY666492 |
| *Accipiter gentilis* | 679 | KBNA853-04 | DQ433279 |
| *Accipiter gentilis* | 694 | TZBNA293-03 | AY666498 |
| *Accipiter gentilis* | 551 | KBNA791-04 | DQ433278 |
| *Accipiter gentilis* | 636 | KBNA790-04 | DQ433276 |
| *Accipiter gentilis* | 633 | KBNA733-04 | DQ433277 |
| *Accipiter striatus* | 694 | KBNA319-04 | DQ434243 |
| *Accipiter striatus* | 693 | TZBNA238-03 | AY666337 |
| *Accipiter striatus* | 694 | KBNA490-04 | DQ433281 |
| *Accipiter striatus* | 693 | KBNA681-04 | DQ434244 |
| *Actitis macularius* | 692 | TZBNA140-03 | AY666270 |
| *Actitis macularius* | 692 | TZBNA131-03 | AY666330 |
| *Actitis macularius* | 694 | KBNA699-04 | DQ434245 |
| *Actitis macularius* | 694 | KBNA698-04 | DQ434246 |
| *Actitis macularius* | 694 | KBNA697-04 | DQ434247 |
| *Actitis macularius* | 651 | BOTW059-04 | DQ432696 |
| *Aegolius acadicus* | 694 | TZBNA302-03 | AY666406 |
| *Aegolius acadicus* | 679 | KKBNA073-04 | DQ433287 |
| *Aegolius acadicus* | 671 | KKBNA239-05 | DQ433285 |
| *Aegolius acadicus* | 694 | TZBNA294-03 | AY666500 |
| *Aegolius acadicus* | 676 | KKBNA238-05 | DQ433286 |
| *Aegolius funereus* | 666 | TZBNA242-03 | AY666416 |
| *Aegolius funereus* | 668 | TZBNA289-03 | AY666506 |
| *Aix sponsa* | 658 | BRDC055-04 | DQ434254 |
| *Aix sponsa* | 648 | BRDC054-04 | DQ434255 |
| *Aix sponsa* | 648 | BRDC053-04 | DQ434256 |
| *Aix sponsa* | 644 | BRDC052-04 | DQ434257 |
| *Aix sponsa* | 694 | TZBNA268-03 | AY666547 |
| *Aix sponsa* | 664 | BRDC051-04 | DQ434253 |
| *Aix sponsa* | 694 | TZBNA257-03 | AY666569 |
| *Aix sponsa* | 661 | TZBNA295-03 | AY666488 |
| *Aix sponsa* | 589 | CDLSU003-05 | DQ432703 |
| *Alectoris chukar* | 662 | HCBR170-03 | AY666409 |
| *Alectoris chukar* | 606 | BOTW212-04 | DQ432706 |
| *Anas acuta* | 668 | BRDC050-04 | DQ434260 |
| *Anas acuta* | 653 | BRDC049-04 | DQ434262 |
| *Anas acuta* | 688 | BRDC048-04 | DQ434264 |
| *Anas acuta* | 664 | BRDC047-04 | DQ434261 |
| *Anas acuta* | 639 | BRDC046-04 | DQ434263 |
| *Anas acuta* | 643 | BOTW193-04 | DQ432716 |
| *Anas acuta* | 614 | CDLSU007-05 | DQ432717 |
| *Anas americana* | 664 | BRDC030-04 | DQ434268 |
| *Anas americana* | 688 | BRDC029-04 | DQ434265 |
| *Anas americana* | 688 | BRDC028-04 | DQ434266 |
| *Anas americana* | 653 | BRDC027-04 | DQ434269 |
| *Anas americana* | 688 | BRDC026-04 | DQ434267 |
| *Anas americana* | 694 | KBNA545-04 | DQ433309 |
| *Anas americana* | 694 | KBNA544-04 | DQ433310 |
| *Anas americana* | 694 | BOTW007-04 | DQ432718 |
| *Anas carolinensis* | 664 | BRDC035-04 | DQ434276 |
| *Anas carolinensis* | 688 | BRDC034-04 | DQ434275 |
| *Anas carolinensis* | 522 | BRDC033-04 | DQ434279 |
| *Anas carolinensis* | 644 | BRDC032-04 | DQ434277 |
| *Anas carolinensis* | 638 | BRDC031-04 | DQ434278 |
| *Anas carolinensis* | 694 | KBNA230-04 | DQ434281 |
| *Anas carolinensis* | 694 | KBNA229-04 | DQ434280 |
| *Anas carolinensis* | 649 | HCBR167-03 | AY666405 |
| *Anas carolinensis* | 669 | BOTW006-04 | DQ432720 |
| *Anas clypeata* | 693 | TZBNA043-03 | AY666352 |
| *Anas clypeata* | 663 | BRDC045-04 | DQ434271 |
| *Anas clypeata* | 688 | BRDC044-04 | DQ434272 |
| *Anas clypeata* | 688 | BRDC043-04 | DQ434273 |
| *Anas clypeata* | 664 | BRDC042-04 | DQ434270 |
| *Anas clypeata* | 688 | BRDC041-04 | DQ434274 |
| *Anas clypeata* | 693 | TZBNA050-03 | AY666360 |
| *Anas discors* | 664 | BRDC040-04 | DQ434282 |
| *Anas discors* | 648 | BRDC039-04 | DQ434284 |
| *Anas discors* | 664 | BRDC038-04 | DQ434283 |
| *Anas discors* | 648 | BRDC037-04 | DQ434285 |
| *Anas discors* | 648 | BRDC036-04 | DQ434286 |
| *Anas discors* | 692 | TZBNA068-03 | AY666323 |
| *Anas discors* | 694 | TZBNA059-03 | AY666325 |
| *Anas discors* | 652 | BOTW063-04 | DQ432722 |
| *Anas platyrhynchos* | 648 | BOTW198-04 | DQ432724 |
| *Anas platyrhynchos* | 694 | TZBNA296-03 | AY666490 |
| *Anas platyrhynchos* | 694 | TZBNA292-03 | AY666496 |
| *Anas platyrhynchos* | 664 | BRDC015-04 | DQ434289 |
| *Anas platyrhynchos* | 688 | BRDC014-04 | DQ434291 |
| *Anas platyrhynchos* | 664 | BRDC013-04 | DQ434288 |
| *Anas platyrhynchos* | 688 | BRDC012-04 | DQ434287 |
| *Anas platyrhynchos* | 664 | BRDC011-04 | DQ434290 |
| *Anas strepera* | 620 | BOTW194-04 | DQ432726 |
| *Anas strepera* | 664 | BRDC025-04 | DQ434299 |
| *Anas strepera* | 688 | BRDC024-04 | DQ434301 |
| *Anas strepera* | 648 | BRDC023-04 | DQ434300 |
| *Anas strepera* | 688 | BRDC022-04 | DQ434298 |
| *Anas strepera* | 686 | BRDC021-04 | DQ434297 |
| *Anser albifrons* | 694 | KBNA579-04 | DQ433314 |
| *Anser albifrons* | 602 | CDLSU010-05 | DQ432730 |
| *Anthus rubescens* | 657 | TZBNA066-03 | AY666215 |
| *Anthus rubescens* | 694 | BOTW016-04 | DQ432731 |
| *Anthus spragueii* | 694 | CDANS010-05 | DQ433315 |
| *Ardea alba* | 651 | BOTW091-04 | DQ432747 |
| *Ardea herodias* | 668 | TZBNA076-03 | AY666320 |
| *Ardea herodias* | 694 | KBNA315-04 | DQ434302 |
| *Ardea herodias* | 694 | KBNA461-04 | DQ433329 |
| *Ardea herodias* | 652 | BOTW180-04 | DQ432748 |
| *Arenaria interpres* | 696 | TZBNA400-03 | NC_003712 |
| *Arenaria interpres* | 637 | HCBR177-04 | AY666561 |
| *Arenaria interpres* | 619 | BOTW060-04 | DQ432749 |
| *Arenaria melanocephala* | 692 | TZBNA213-03 | AY666260 |
| *Asio flammeus* | 694 | KBNA458-04 | DQ433332 |
| *Asio flammeus* | 640 | KKBNA068-04 | DQ433331 |
| *Asio flammeus* | 650 | KKBNA164-04 | DQ433330 |
| *Asio flammeus* | 693 | KBNA507-04 | DQ433334 |
| *Asio flammeus* | 694 | KBNA005-04 | DQ433333 |
| *Asio otus* | 693 | TZBNA260-03 | AY666568 |
| *Asio otus* | 689 | KBNA460-04 | DQ433336 |
| *Asio otus* | 597 | CDLSU014-05 | DQ432752 |
| *Asio otus* | 669 | KKBNA063-04 | DQ433335 |
| *Asio otus* | 694 | KBNA478-04 | DQ433337 |
| *Athene cunicularia* | 652 | BOTW069-04 | DQ432754 |
| *Athene cunicularia* | 672 | KKBNA053-04 | DQ433340 |
| *Aythya affinis* | 694 | BRDC157-05 | DQ434308 |
| *Aythya affinis* | 694 | BRDC156-05 | DQ434307 |
| *Aythya affinis* | 694 | BRDC155-05 | DQ434306 |
| *Aythya affinis* | 653 | BRDC075-04 | DQ434305 |
| *Aythya affinis* | 664 | BRDC074-04 | DQ434303 |
| *Aythya affinis* | 664 | BRDC073-04 | DQ434304 |
| *Aythya affinis* | 687 | BRDC072-04 | DQ434309 |
| *Aythya affinis* | 688 | BRDC071-04 | DQ434310 |
| *Aythya affinis* | 636 | BOTW008-04 | DQ432756 |
| *Aythya americana* | 696 | TZBNA402-03 | NC_000877 |
| *Aythya americana* | 694 | BRDC148-05 | DQ434314 |
| *Aythya americana* | 694 | BRDC147-05 | DQ434313 |
| *Aythya americana* | 694 | BRDC145-05 | DQ434316 |
| *Aythya americana* | 694 | BRDC144-05 | DQ434315 |
| *Aythya americana* | 667 | BRDC060-04 | AY666572 |
| *Aythya americana* | 684 | BRDC059-04 | DQ434311 |
| *Aythya americana* | 688 | BRDC057-04 | DQ434317 |
| *Aythya americana* | 653 | BRDC056-04 | DQ434312 |
| *Aythya collaris* | 694 | BRDC162-05 | DQ434324 |
| *Aythya collaris* | 694 | BRDC161-05 | DQ434323 |
| *Aythya collaris* | 694 | BRDC160-05 | DQ434322 |
| *Aythya collaris* | 694 | TZBNA074-03 | DQ434325 |
| *Aythya collaris* | 581 | BOTW196-04 | DQ432758 |
| *Aythya collaris* | 666 | BRDC080-04 | DQ434320 |
| *Aythya collaris* | 666 | BRDC079-04 | DQ434318 |
| *Aythya collaris* | 666 | BRDC078-04 | DQ434319 |
| *Aythya collaris* | 438 | BRDC077-04 | DQ434321 |
| *Aythya collaris* | 688 | BRDC076-04 | DQ434326 |
| *Aythya fuligula* | 668 | KKBNA350-05 | DQ433344 |
| *Aythya fuligula* | 617 | KKBNA449-05 | DQ433343 |
| *Aythya fuligula* | 672 | KKBNA617-05 | DQ433345 |
| *Aythya marila* | 624 | BOTW199-04 | DQ432759 |
| *Aythya marila* | 694 | BRDC154-05 | DQ434334 |
| *Aythya marila* | 694 | BRDC153-05 | DQ434333 |
| *Aythya marila* | 694 | BRDC152-05 | DQ434332 |
| *Aythya marila* | 694 | BRDC150-05 | DQ434331 |
| *Aythya marila* | 688 | BRDC070-04 | DQ434327 |
| *Aythya marila* | 491 | BRDC069-04 | DQ434330 |
| *Aythya marila* | 656 | BRDC068-04 | DQ434329 |
| *Aythya marila* | 688 | BRDC067-04 | DQ434335 |
| *Aythya marila* | 664 | BRDC066-04 | DQ434328 |
| *Aythya valisineria* | 652 | BOTW065-04 | DQ432760 |
| *Aythya valisineria* | 668 | BRDC065-04 | DQ434336 |
| *Aythya valisineria* | 653 | BRDC064-04 | DQ434338 |
| *Aythya valisineria* | 651 | BRDC063-04 | DQ434339 |
| *Aythya valisineria* | 651 | BRDC062-04 | DQ434337 |
| *Aythya valisineria* | 648 | BRDC061-04 | DQ434340 |
| *Bombycilla cedrorum* | 657 | TZBNA037-03 | AY666345 |
| *Bombycilla cedrorum* | 671 | KBNA797-04 | DQ433347 |
| *Bombycilla cedrorum* | 678 | KKBNA881-05 | DQ433349 |
| *Bombycilla cedrorum* | 608 | KKBNA880-05 | DQ433348 |
| *Bombycilla garrulus* | 656 | HCBR163-03 | AY666413 |
| *Bonasa umbellus* | 694 | KBNA473-04 | DQ433351 |
| *Bonasa umbellus* | 682 | TZBNA580-04 | AY666563 |
| *Bonasa umbellus* | 696 | TZBNA167-03 | AY666214 |
| *Bonasa umbellus* | 694 | KBNA264-04 | DQ434343 |
| *Bonasa umbellus* | 631 | BOTW214-04 | DQ432768 |
| *Brachyramphus brevirostris* | 609 | CDAMH015-05 | DQ432770 |
| *Brachyramphus brevirostris* | 600 | CDAMH014-05 | DQ432771 |
| *Brachyramphus brevirostris* | 670 | KKBNA264-05 | DQ433355 |
| *Brachyramphus brevirostris* | 694 | KKBNA263-05 | DQ433358 |
| *Brachyramphus brevirostris* | 671 | KKBNA262-05 | DQ433354 |
| *Brachyramphus brevirostris* | 670 | KKBNA261-05 | DQ433356 |
| *Brachyramphus marmoratus* | 694 | KBNA591-04 | DQ433360 |
| *Brachyramphus marmoratus* | 694 | KBNA590-04 | DQ433361 |
| *Bucephala albeola* | 694 | TZBNA263-03 | AY666564 |
| *Bucephala albeola* | 688 | BRDC095-04 | DQ434487 |
| *Bucephala albeola* | 688 | BRDC094-04 | DQ434491 |
| *Bucephala albeola* | 688 | BRDC093-04 | DQ434488 |
| *Bucephala albeola* | 656 | BRDC092-04 | DQ434490 |
| *Bucephala albeola* | 651 | BRDC091-04 | DQ434489 |
| *Bucephala albeola* | 626 | TZBNA061-03 | AY666424 |
| *Bucephala albeola* | 694 | BOTW009-04 | DQ432777 |
| *Bucephala clangula* | 623 | BOTW202-04 | DQ432778 |
| *Bucephala clangula* | 491 | BRDC085-04 | DQ434496 |
| *Bucephala clangula* | 668 | BRDC084-04 | DQ434493 |
| *Bucephala clangula* | 688 | BRDC083-04 | DQ434492 |
| *Bucephala clangula* | 666 | BRDC082-04 | DQ434494 |
| *Bucephala clangula* | 663 | BRDC081-04 | DQ434495 |
| *Bucephala islandica* | 694 | BRDC164-05 | DQ434502 |
| *Bucephala islandica* | 694 | BRDC163-05 | DQ434501 |
| *Bucephala islandica* | 694 | KBNA523-04 | DQ433387 |
| *Bucephala islandica* | 694 | KBNA518-04 | DQ433386 |
| *Bucephala islandica* | 688 | BRDC090-04 | DQ434497 |
| *Bucephala islandica* | 688 | BRDC089-04 | DQ434498 |
| *Bucephala islandica* | 656 | BRDC088-04 | DQ434500 |
| *Bucephala islandica* | 688 | BRDC087-04 | DQ434499 |
| *Bucephala islandica* | 688 | BRDC086-04 | DQ434503 |
| *Bucephala islandica* | 652 | BOTW066-04 | DQ432779 |
| *Buteo albicaudatus* | 670 | KKBNA952-05 | DQ433389 |
| *Buteo brachyurus* | 652 | BOTW342-05 | DQ432783 |
| *Buteo jamaicensis* | 694 | KBNA667-04 | DQ434504 |
| *Buteo lagopus* | 694 | TZBNA285-03 | AY666502 |
| *Buteo lagopus* | 552 | TZBNA210-03 | AY666290 |
| *Buteo lineatus* | 652 | BOTW024-04 | DQ432786 |
| *Buteo lineatus* | 670 | TZBNA003-03 | AY666276 |
| *Buteo nitidus* | 694 | KKBNA814-05 | DQ433339 |
| *Buteo platypterus* | 694 | TZBNA334-03 | AY666390 |
| *Buteo platypterus* | 688 | TZBNA328-03 | AY666398 |
| *Buteo regalis* | 644 | KBNA789-04 | DQ433390 |
| *Buteo swainsoni* | 624 | BOTW203-04 | DQ432788 |
| *Buteo swainsoni* | 652 | BOTW344-05 | DQ432789 |
| *Buteo swainsoni* | 673 | KBNA737-04 | DQ433391 |
| *Butorides virescens* | 694 | KBNA455-04 | DQ433392 |
| *Calcarius lapponicus* | 575 | BOTW103-04 | DQ432794 |
| *Calcarius lapponicus* | 694 | CDANS021-05 | DQ433397 |
| *Calcarius lapponicus* | 694 | CDANS020-05 | DQ433398 |
| *Calcarius mccownii* | 669 | KKBNA492-05 | DQ433400 |
| *Calcarius mccownii* | 694 | KKBNA491-05 | DQ433401 |
| *Calcarius mccownii* | 679 | KKBNA490-05 | DQ433402 |
| *Calcarius mccownii* | 686 | CDANS019-05 | DQ433399 |
| *Calcarius ornatus* | 670 | KKBNA489-05 | DQ433407 |
| *Calcarius ornatus* | 669 | KKBNA488-05 | DQ433405 |
| *Calcarius ornatus* | 669 | KKBNA485-05 | DQ433406 |
| *Calcarius ornatus* | 575 | CDLSU019-05 | DQ432795 |
| *Calcarius ornatus* | 670 | KKBNA484-05 | DQ433408 |
| *Calcarius ornatus* | 694 | CDANS017-05 | DQ433403 |
| *Calcarius ornatus* | 678 | KBNA838-04 | DQ433404 |
| *Calcarius pictus* | 599 | CDLSU020-05 | DQ432796 |
| *Calcarius pictus* | 673 | KKBNA662-05 | DQ433409 |
| *Calidris alba* | 652 | BOTW049-04 | DQ432797 |
| *Calidris alba* | 694 | TZBNA106-03 | AY666412 |
| *Calidris alba* | 694 | TZBNA097-03 | AY666377 |
| *Calidris alpina* | 652 | BOTW050-04 | DQ432798 |
| *Calidris alpina* | 694 | TZBNA115-03 | AY666371 |
| *Calidris alpina* | 654 | HCBR154-03 | AY666415 |
| *Calidris bairdii* | 692 | TZBNA124-03 | AY666373 |
| *Calidris bairdii* | 694 | TZBNA111-03 | AY666355 |
| *Calidris canutus* | 692 | TZBNA133-03 | AY666343 |
| *Calidris canutus* | 644 | BOTW061-04 | DQ432799 |
| *Calidris fuscicollis* | 692 | TZBNA151-03 | AY666305 |
| *Calidris fuscicollis* | 530 | TZBNA142-03 | AY666319 |
| *Calidris himantopus* | 694 | TZBNA141-03 | AY666359 |
| *Calidris himantopus* | 694 | CDANS023-05 | DQ433410 |
| *Calidris mauri* | 592 | CDLSU021-05 | DQ432802 |
| *Calidris mauri* | 652 | BOTW324-05 | DQ432803 |
| *Calidris mauri* | 692 | TZBNA211-03 | AY666261 |
| *Calidris melanotos* | 560 | TZBNA105-03 | AY666262 |
| *Calidris melanotos* | 694 | TZBNA087-03 | AY666264 |
| *Calidris melanotos* | 692 | TZBNA116-03 | AY666286 |
| *Calidris minutilla* | 644 | BOTW052-04 | DQ432804 |
| *Calidris minutilla* | 692 | TZBNA114-03 | AY666246 |
| *Calidris minutilla* | 693 | TZBNA096-03 | AY666272 |
| *Calidris ptilocnemis* | 653 | HCBR152-03 | DQ433413 |
| *Calidris ptilocnemis* | 670 | HCBR151-03 | DQ433414 |
| *Calidris pusilla* | 644 | BOTW053-04 | DQ432805 |
| *Calidris pusilla* | 692 | TZBNA132-03 | AY666222 |
| *Calidris pusilla* | 673 | TZBNA123-03 | AY666224 |
| *Caprimulgus carolinensis* | 565 | TZBNA271-03 | AY666541 |
| *Caprimulgus carolinensis* | 639 | BOTW086-04 | DQ432814 |
| *Caprimulgus vociferus* | 615 | CDLSU022-05 | DQ432815 |
| *Caprimulgus vociferus* | 694 | TZBNA206-03 | AY666179 |
| *Caprimulgus vociferus* | 694 | TZBNA283-03 | AY666512 |
| *Caprimulgus vociferus* | 663 | HCBR150-03 | AY666421 |
| *Carduelis flammea* | 693 | TZBNA312-03 | AY666474 |
| *Carduelis flammea* | 667 | KKBNA894-05 | DQ433427 |
| *Carduelis hornemanni* | 670 | KKBNA278-05 | DQ433429 |
| *Carduelis hornemanni* | 671 | KKBNA279-05 | DQ433431 |
| *Carduelis hornemanni* | 679 | KKBNA312-05 | DQ433432 |
| *Carduelis hornemanni* | 672 | KKBNA313-05 | DQ433430 |
| *Carduelis hornemanni* | 670 | KKBNA540-05 | DQ433428 |
| *Carduelis lawrencei* | 694 | TZBNA138-03 | AY666229 |
| *Carduelis lawrencei* | 588 | CDMVZ003-05 | DQ432820 |
| *Carduelis lawrencei* | 588 | CDMVZ002-05 | DQ432819 |
| *Carduelis pinus* | 658 | HCBR147-03 | AY666429 |
| *Carduelis pinus* | 677 | KBNA301-04 | DQ434513 |
| *Carduelis pinus* | 694 | KBNA298-04 | DQ434512 |
| *Carduelis pinus* | 694 | KBNA396-04 | DQ434511 |
| *Carduelis pinus* | 623 | BOTW116-04 | DQ432821 |
| *Carduelis pinus* | 694 | TZBNA276-03 | AY666535 |
| *Carduelis tristis* | 694 | KBNA302-04 | DQ434516 |
| *Carduelis tristis* | 694 | TZBNA062-03 | AY666200 |
| *Carduelis tristis* | 694 | TZBNA033-03 | AY666192 |
| *Carduelis tristis* | 694 | KBNA147-04 | DQ434514 |
| *Carduelis tristis* | 657 | TZBNA407-03 | AY666581 |
| *Carduelis tristis* | 693 | KBNA054-04 | DQ434517 |
| *Carduelis tristis* | 626 | BOTW108-04 | DQ432822 |
| *Carduelis tristis* | 694 | KBNA053-04 | DQ434515 |
| *Carpodacus cassinii* | 673 | KKBNA447-05 | DQ433436 |
| *Carpodacus cassinii* | 670 | KKBNA496-05 | DQ433433 |
| *Carpodacus cassinii* | 670 | KKBNA545-05 | DQ433435 |
| *Carpodacus cassinii* | 670 | KKBNA483-05 | DQ433434 |
| *Carpodacus mexicanus* | 641 | BOTW104-04 | DQ432823 |
| *Carpodacus mexicanus* | 564 | HCBR144-03 | AY666596 |
| *Carpodacus mexicanus* | 694 | KBNA370-04 | DQ434518 |
| *Carpodacus mexicanus* | 693 | TZBNA179-03 | AY666266 |
| *Carpodacus mexicanus* | 694 | KBNA148-04 | DQ434519 |
| *Carpodacus purpureus* | 656 | HCBR145-03 | AY666431 |
| *Carpodacus purpureus* | 694 | KBNA296-04 | DQ434520 |
| *Carpodacus purpureus* | 694 | KBNA397-04 | DQ434521 |
| *Carpodacus purpureus* | 693 | TZBNA042-03 | AY666335 |
| *Carpodacus purpureus* | 683 | KBNA151-04 | DQ434522 |
| *Carpodacus purpureus* | 694 | TZBNA041-03 | AY666382 |
| *Cepphus columba* | 694 | KBNA594-04 | DQ433467 |
| *Cepphus columba* | 694 | KBNA593-04 | DQ433468 |
| *Cepphus grylle* | 694 | KBNA588-04 | DQ433470 |
| *Cepphus grylle* | 606 | KBNA587-04 | DQ433469 |
| *Certhia americana* | 662 | HCBR141-03 | AY666487 |
| *Certhia americana* | 662 | HCBR140-03 | AY666473 |
| *Certhia americana* | 694 | KBNA032-04 | DQ434534 |
| *Certhia americana* | 647 | KBNA031-04 | DQ434536 |
| *Certhia americana* | 508 | KBNA799-04 | DQ433476 |
| *Certhia americana* | 694 | KBNA334-04 | DQ434535 |
| *Charadrius alexandrinus* | 609 | CDLSU023-05 | DQ432842 |
| *Charadrius alexandrinus* | 692 | TZBNA110-03 | AY666380 |
| *Charadrius alexandrinus* | 692 | TZBNA101-03 | AY666381 |
| *Charadrius melodus* | 692 | TZBNA092-03 | AY666253 |
| *Charadrius melodus* | 694 | TZBNA083-03 | AY666273 |
| *Charadrius melodus* | 694 | KBNA612-04 | DQ433490 |
| *Charadrius melodus* | 694 | KBNA611-04 | DQ433491 |
| *Charadrius melodus* | 694 | KBNA610-04 | DQ433488 |
| *Charadrius melodus* | 694 | KBNA609-04 | DQ433489 |
| *Charadrius montanus* | 694 | TZBNA156-03 | AY666249 |
| *Charadrius montanus* | 694 | TZBNA147-03 | AY666259 |
| *Charadrius semipalmatus* | 655 | HCBR137-03 | AY666435 |
| *Charadrius semipalmatus* | 652 | BOTW041-04 | DQ432846 |
| *Charadrius semipalmatus* | 694 | KBNA615-04 | DQ433493 |
| *Charadrius semipalmatus* | 694 | KBNA614-04 | DQ433494 |
| *Charadrius vociferus* | 623 | BOTW042-04 | DQ432847 |
| *Charadrius vociferus* | 692 | TZBNA128-03 | AY666172 |
| *Charadrius vociferus* | 692 | TZBNA119-03 | AY666173 |
| *Charadrius wilsonia* | 694 | TZBNA127-03 | AY666175 |
| *Charadrius wilsonia* | 652 | BOTW314-05 | DQ432848 |
| *Chlidonias niger* | 670 | KKBNA149-04 | DQ433499 |
| *Chlidonias niger* | 694 | TZBNA091-03 | AY666251 |
| *Cinclus mexicanus* | 652 | BOTW320-05 | DQ432853 |
| *Cinclus mexicanus* | 694 | KKBNA296-05 | DQ433511 |
| *Cinclus mexicanus* | 670 | KKBNA295-05 | DQ433508 |
| *Cinclus mexicanus* | 694 | KKBNA294-05 | DQ433512 |
| *Cinclus mexicanus* | 678 | KBNA696-04 | DQ434541 |
| *Cinclus mexicanus* | 670 | KKBNA507-05 | DQ433509 |
| *Cinclus mexicanus* | 670 | KKBNA506-05 | DQ433507 |
| *Cinclus mexicanus* | 658 | KKBNA188-05 | DQ433510 |
| *Circus cyaneus* | 673 | KBNA928-04 | DQ433513 |
| *Circus cyaneus* | 600 | CDLSU025-05 | DQ432854 |
| *Circus cyaneus* | 605 | HCBR135-03 | AY666437 |
| *Circus cyaneus* | 605 | HCBR134-03 | AY666427 |
| *Clangula hyemalis* | 694 | BRDC166-05 | DQ434548 |
| *Clangula hyemalis* | 694 | BRDC165-05 | DQ434547 |
| *Clangula hyemalis* | 491 | BRDC100-04 | DQ434546 |
| *Clangula hyemalis* | 688 | BRDC099-04 | DQ434543 |
| *Clangula hyemalis* | 667 | BRDC098-04 | DQ434544 |
| *Clangula hyemalis* | 618 | BRDC097-04 | DQ434545 |
| *Clangula hyemalis* | 694 | BRDC169-05 | DQ434551 |
| *Clangula hyemalis* | 694 | BRDC168-05 | DQ434550 |
| *Clangula hyemalis* | 694 | BRDC167-05 | DQ434549 |
| *Coccothraustes vespertinus* | 658 | HCBR130-03 | AY666443 |
| *Coccothraustes vespertinus* | 661 | HCBR129-03 | AY666433 |
| *Columba livia* | 694 | TZBNA301-03 | AY666494 |
| *Columba livia* | 650 | BOTW080-04 | DQ432860 |
| *Corvus brachyrhynchos* | 678 | TZBNA303-03 | AY666408 |
| *Corvus brachyrhynchos* | 687 | TZBNA304-03 | AY666482 |
| *Corvus brachyrhynchos* | 652 | BOTW110-04 | DQ432866 |
| *Corvus corax PS-1* | 671 | KBNA004-04 | DQ433551 |
| *Corvus corax PS-1* | 628 | KBNA785-04 | DQ433552 |
| *Corvus corax PS-1* | 643 | BOTW106-04 | DQ432868 |
| *Corvus cryptoleucus* | 621 | CDAMH033-05 | DQ432872 |
| *Corvus ossifragus* | 652 | BOTW317-05 | DQ432873 |
| *Corvus ossifragus* | 652 | BOTW107-04 | DQ432874 |
| *Dryocopus pileatus* | 693 | TZBNA379-03 | AY666418 |
| *Dryocopus pileatus* | 693 | TZBNA374-03 | AY666388 |
| *Dryocopus pileatus* | 694 | KBNA483-04 | DQ433594 |
| *Egretta caerulea* | 652 | BOTW354-05 | DQ432900 |
| *Egretta caerulea* | 652 | BOTW353-05 | DQ432901 |
| *Egretta tricolor* | 652 | BOTW356-05 | DQ432903 |
| *Egretta tricolor* | 652 | BOTW355-05 | DQ432904 |
| *Egretta tricolor* | 624 | CDAMH041-05 | DQ432905 |
| *Eremophila alpestris* | 651 | BOTW167-04 | DQ432916 |
| *Eremophila alpestris* | 693 | HCBR101-03 | AY666513 |
| *Falco columbarius* | 694 | KBNA513-04 | DQ434594 |
| *Falco columbarius* | 624 | BOTW185-04 | DQ432924 |
| *Falco columbarius* | 663 | KBNA263-04 | DQ434593 |
| *Falco columbarius* | 687 | TZBNA281-03 | AY666522 |
| *Falco mexicanus* | 696 | TZBNA265-03 | AY666553 |
| *Falco mexicanus* | 652 | BOTW188-04 | DQ432926 |
| *Falco peregrinus* | 696 | TZBNA403-03 | NC_000878 |
| *Falco peregrinus* | 696 | TZBNA278-03 | AY666529 |
| *Falco rusticolus* | 678 | KBNA872-04 | DQ433640 |
| *Falco rusticolus* | 652 | BOTW186-04 | DQ432927 |
| *Falco sparverius* | 694 | KBNA317-04 | DQ434595 |
| *Falco sparverius* | 578 | KBNA927-04 | DQ433641 |
| *Falco sparverius* | 653 | HCBR099-03 | AY666467 |
| *Falco sparverius* | 694 | BOTW005-04 | DQ432928 |
| *Falco sparverius* | 655 | HCBR098-03 | AY666469 |
| *Fratercula cirrhata* | 694 | TZBNA226-03 | AY666344 |
| *Fratercula cirrhata* | 670 | KKBNA162-04 | DQ433644 |
| *Fratercula corniculata* | 594 | CDAMH046-05 | DQ432930 |
| *Fratercula corniculata* | 694 | TZBNA227-03 | AY666340 |
| *Fringilla montifringilla* | 694 | TZBNA219-03 | AY666258 |
| *Fulica americana* | 669 | KKBNA377-05 | DQ433648 |
| *Fulica americana* | 670 | KKBNA576-05 | DQ433647 |
| *Fulica americana* | 669 | KKBNA197-05 | DQ433649 |
| *Fulica americana* | 694 | KBNA676-04 | DQ434598 |
| *Fulica americana* | 611 | CDLSU036-05 | DQ432932 |
| *Gallinago delicata* | 694 | BOTW001-04 | DQ432935 |
| *Gallinago delicata* | 693 | KBNA672-04 | DQ434599 |
| *Gallinago delicata* | 694 | TZBNA214-03 | AY666304 |
| *Gallinago delicata* | 666 | TZBNA195-03 | AY666292 |
| *Gallinago delicata* | 652 | BOTW245-05 | DQ432934 |
| *Gallinula chloropus* | 694 | KBNA671-04 | DQ434600 |
| *Gallinula chloropus* | 648 | BOTW076-04 | DQ432936 |
| *Gallinula chloropus* | 681 | KBNA890-04 | DQ433657 |
| *Gallinula chloropus* | 678 | KBNA889-04 | DQ433654 |
| *Gallinula chloropus* | 694 | KBNA003-04 | DQ433656 |
| *Gavia adamsii* | 678 | KBNA918-04 | DQ433663 |
| *Gavia adamsii* | 640 | KKBNA336-05 | DQ433658 |
| *Gavia adamsii* | 679 | KKBNA335-05 | DQ433662 |
| *Gavia adamsii* | 671 | KKBNA334-05 | DQ433660 |
| *Gavia adamsii* | 672 | KKBNA283-05 | DQ433661 |
| *Gavia adamsii* | 669 | KKBNA333-05 | DQ433659 |
| *Gavia immer* | 679 | KBNA892-04 | DQ433665 |
| *Gavia immer* | 597 | CDLSU038-05 | DQ432937 |
| *Gavia immer* | 656 | TZBNA188-03 | AY666287 |
| *Gavia pacifica* | 671 | KKBNA751-05 | DQ433668 |
| *Gavia pacifica* | 652 | BOTW265-05 | DQ432938 |
| *Gavia pacifica* | 432 | KKBNA519-05 | DQ433670 |
| *Gavia pacifica* | 678 | KKBNA467-05 | DQ433669 |
| *Gavia stellata* | 658 | HCBR096-03 | AY666477 |
| *Gavia stellata* | 654 | HCBR097-03 | AY666471 |
| *Haematopus palliatus* | 677 | TZBNA118-03 | AY666244 |
| *Haematopus palliatus* | 694 | TZBNA084-03 | AY666233 |
| *Hirundo rustica* | 668 | HCBR090-03 | AY666485 |
| *Hirundo rustica* | 668 | HCBR089-03 | AY666483 |
| *Hirundo rustica* | 694 | KBNA439-04 | DQ434603 |
| *Histrionicus histrionicus* | 694 | KBNA693-04 | DQ434604 |
| *Histrionicus histrionicus* | 694 | KBNA692-04 | DQ434605 |
| *Ixobrychus exilis* | 671 | KKBNA898-05 | DQ433699 |
| *Ixobrychus exilis* | 669 | KKBNA897-05 | DQ433698 |
| *Lagopus lagopus* | 694 | KBNA960-04 | DQ433710 |
| *Lagopus lagopus* | 694 | KBNA959-04 | DQ433711 |
| *Lagopus lagopus* | 694 | KBNA505-04 | DQ433712 |
| *Lagopus leucura* | 670 | KKBNA742-05 | DQ433714 |
| *Lagopus leucura* | 672 | KKBNA739-05 | DQ433718 |
| *Lagopus leucura* | 678 | KKBNA254-05 | DQ433716 |
| *Lagopus leucura* | 673 | KKBNA253-05 | DQ433717 |
| *Lagopus leucura* | 670 | KKBNA555-05 | DQ433715 |
| *Lagopus muta* | 512 | KKBNA786-05 | DQ433735 |
| *Lagopus muta* | 676 | KKBNA785-05 | DQ433732 |
| *Lagopus muta* | 669 | KKBNA784-05 | DQ433730 |
| *Lagopus muta* | 675 | KKBNA783-05 | DQ433733 |
| *Lagopus muta* | 680 | KKBNA782-05 | DQ433738 |
| *Lagopus muta* | 670 | KKBNA781-05 | DQ433726 |
| *Lagopus muta* | 657 | KKBNA438-05 | DQ433731 |
| *Lagopus muta* | 680 | KKBNA779-05 | DQ433736 |
| *Lagopus muta* | 673 | KKBNA778-05 | DQ433734 |
| *Lagopus muta* | 648 | KKBNA434-05 | DQ433722 |
| *Lagopus muta* | 680 | KKBNA776-05 | DQ433737 |
| *Lagopus muta* | 648 | KKBNA775-05 | DQ433721 |
| *Lagopus muta* | 649 | KKBNA433-05 | DQ433720 |
| *Lagopus muta* | 634 | KKBNA774-05 | DQ433723 |
| *Lagopus muta* | 605 | KKBNA773-05 | DQ433724 |
| *Lagopus muta* | 605 | KKBNA772-05 | DQ433725 |
| *Lagopus muta* | 678 | KBNA814-04 | DQ433719 |
| *Lagopus muta* | 679 | KBNA958-04 | DQ433739 |
| *Lanius excubitor* | 693 | TZBNA184-03 | AY666268 |
| *Lanius excubitor* | 496 | CDLSU042-05 | DQ432961 |
| *Lanius excubitor* | 694 | KBNA481-04 | DQ433740 |
| *Lanius ludovicianus* | 659 | TZBNA329-03 | AY666464 |
| *Lanius ludovicianus* | 639 | BOTW140-04 | DQ432962 |
| *Lanius ludovicianus* | 623 | CDUSM062-05 | DQ432965 |
| *Lanius ludovicianus* | 623 | CDUSM061-05 | DQ432964 |
| *Lanius ludovicianus* | 692 | TZBNA337-03 | AY666394 |
| *Larus atricilla* | 644 | BOTW033-04 | DQ432968 |
| *Larus atricilla* | 610 | CDUSM017-05 | DQ432975 |
| *Larus atricilla* | 616 | CDUSM016-05 | DQ432974 |
| *Larus atricilla* | 627 | CDUSM015-05 | DQ432969 |
| *Larus atricilla* | 620 | CDUSM014-05 | DQ432970 |
| *Larus atricilla* | 617 | CDUSM013-05 | DQ432972 |
| *Larus atricilla* | 619 | CDUSM012-05 | DQ432971 |
| *Larus californicus* | 694 | KBNA624-04 | DQ433744 |
| *Larus californicus* | 652 | BOTW034-04 | DQ432976 |
| *Larus californicus* | 622 | CDUSM009-05 | DQ432977 |
| *Larus canus* | 694 | TZBNA155-03 | AY666353 |
| *Larus canus* | 630 | BOTW035-04 | DQ432980 |
| *Larus canus* | 694 | KBNA655-04 | DQ433746 |
| *Larus canus* | 601 | KBNA654-04 | DQ433745 |
| *Larus delawarensis* | 657 | HCBR085-03 | AY666493 |
| *Larus delawarensis* | 652 | BOTW036-04 | DQ432981 |
| *Larus delawarensis* | 694 | KBNA660-04 | DQ433747 |
| *Larus glaucescens* | 627 | KKBNA177-04 | DQ433750 |
| *Larus glaucescens* | 622 | KKBNA176-04 | DQ433751 |
| *Larus glaucescens* | 650 | BOTW176-04 | DQ432986 |
| *Larus glaucescens* | 567 | KBNA637-04 | DQ433749 |
| *Larus glaucoides* | 694 | TZBNA006-03 | AY666303 |
| *Larus heermanni* | 694 | KBNA643-04 | DQ433752 |
| *Larus heermanni* | 694 | KBNA642-04 | DQ433753 |
| *Larus heermanni* | 694 | KBNA641-04 | DQ433754 |
| *Larus hyperboreus* | 694 | KBNA634-04 | DQ433756 |
| *Larus hyperboreus* | 653 | TZBNA313-03 | AY666476 |
| *Larus hyperboreus* | 577 | CDLSU067-05 | DQ432987 |
| *Larus hyperboreus* | 694 | KBNA635-04 | DQ433755 |
| *Larus marinus* | 652 | BOTW037-04 | DQ432988 |
| *Larus marinus* | 594 | TZBNA363-03 | AY666436 |
| *Larus marinus* | 694 | KBNA640-04 | DQ433757 |
| *Larus occidentalis* | 694 | KBNA666-04 | DQ433758 |
| *Larus occidentalis* | 694 | KBNA665-04 | DQ433759 |
| *Larus occidentalis* | 694 | KBNA664-04 | DQ433760 |
| *Larus occidentalis* | 582 | CDLSU044-05 | DQ432989 |
| *Larus philadelphia* | 635 | KKBNA140-04 | DQ433761 |
| *Larus philadelphia* | 666 | KKBNA139-04 | DQ433763 |
| *Larus philadelphia* | 578 | KKBNA138-04 | DQ433762 |
| *Larus philadelphia* | 599 | CDLSU068-05 | DQ432990 |
| *Larus smithsonianus* | 617 | BOTW032-04 | DQ432966 |
| *Larus smithsonianus* | 688 | TZBNA034-03 | AY666375 |
| *Larus smithsonianus* | 694 | KBNA646-04 | DQ433741 |
| *Larus smithsonianus* | 694 | KBNA645-04 | DQ433742 |
| *Larus smithsonianus* | 694 | KBNA644-04 | DQ433743 |
| *Larus thayeri* | 595 | CDLSU045-05 | DQ432996 |
| *Larus thayeri* | 639 | TZBNA079-03 | AY666191 |
| *Leucosticte tephrocotis* | 574 | KKBNA635-05 | DQ433768 |
| *Leucosticte tephrocotis* | 670 | KKBNA726-05 | DQ433769 |
| *Leucosticte tephrocotis* | 664 | KKBNA725-05 | DQ433770 |
| *Leucosticte tephrocotis* | 671 | KKBNA724-05 | DQ433771 |
| *Limnodromus griseus* | 694 | TZBNA159-03 | AY666454 |
| *Limnodromus griseus* | 625 | CDUSM034-05 | DQ433001 |
| *Limnodromus griseus* | 694 | TZBNA086-03 | AY666297 |
| *Limnodromus griseus* | 621 | CDUSM033-05 | DQ433002 |
| *Limnodromus griseus* | 644 | BOTW055-04 | DQ432999 |
| *Limnodromus scolopaceus* | 694 | TZBNA216-03 | AY666210 |
| *Limnodromus scolopaceus* | 692 | TZBNA215-03 | AY666209 |
| *Limosa fedoa* | 694 | TZBNA103-03 | AY666307 |
| *Limosa fedoa* | 690 | TZBNA094-03 | AY666327 |
| *Limosa fedoa* | 672 | KKBNA160-04 | DQ433773 |
| *Limosa haemastica* | 694 | TZBNA120-03 | AY666318 |
| *Limosa haemastica* | 694 | TZBNA112-03 | AY666302 |
| *Limosa lapponica* | 678 | TZBNA218-03 | AY666279 |
| *Limosa lapponica* | 694 | TZBNA217-03 | AY666291 |
| *Loxia curvirostra* | 652 | BOTW323-05 | DQ433004 |
| *Loxia curvirostra* | 668 | KKBNA899-05 | DQ433774 |
| *Loxia curvirostra* | 693 | TZBNA038-03 | AY666331 |
| *Loxia leucoptera* | 623 | KKBNA900-05 | DQ433775 |
| *Loxia leucoptera* | 692 | TZBNA011-03 | AY666278 |
| *Melanitta fusca* | 477 | BRDC117-04 | DQ434647 |
| *Melanitta fusca* | 567 | BRDC116-04 | DQ434646 |
| *Melanitta fusca* | 614 | BRDC114-04 | DQ434644 |
| *Melanitta fusca* | 653 | BRDC113-04 | DQ434643 |
| *Melanitta fusca* | 611 | BRDC112-04 | DQ434645 |
| *Melanitta nigra* | 664 | BRDC106-04 | DQ434650 |
| *Melanitta nigra* | 690 | TZBNA343-03 | AY666338 |
| *Melanitta nigra* | 665 | BRDC111-04 | DQ434648 |
| *Melanitta nigra* | 603 | BRDC110-04 | DQ434652 |
| *Melanitta nigra* | 664 | BRDC109-04 | DQ434649 |
| *Melanitta nigra* | 656 | BRDC108-04 | DQ434651 |
| *Melanitta nigra* | 522 | BRDC107-04 | DQ434653 |
| *Melanitta perspicillata* | 680 | BRDC119-04 | DQ434654 |
| *Melanitta perspicillata* | 509 | BRDC118-04 | DQ434655 |
| *Mergus merganser* | 649 | BRDC004-04 | DQ434669 |
| *Mergus merganser* | 651 | BRDC003-04 | DQ434668 |
| *Mergus merganser* | 688 | BRDC002-04 | DQ434673 |
| *Mergus merganser* | 688 | BRDC001-04 | DQ434666 |
| *Mergus merganser* | 694 | BRDC140-05 | DQ434672 |
| *Mergus merganser* | 694 | BRDC139-05 | DQ434671 |
| *Mergus merganser* | 694 | BRDC138-05 | DQ434670 |
| *Mergus merganser* | 686 | BRDC137-05 | DQ434667 |
| *Mergus merganser* | 583 | CDLSU047-05 | DQ433020 |
| *Mergus serrator* | 694 | TZBNA016-03 | AY666208 |
| *Mergus serrator* | 694 | BRDC143-05 | DQ434677 |
| *Mergus serrator* | 694 | BRDC142-05 | DQ434676 |
| *Mergus serrator* | 694 | BRDC141-05 | DQ434675 |
| *Mergus serrator* | 693 | TZBNA245-03 | AY666588 |
| *Mergus serrator* | 664 | BRDC005-04 | DQ434674 |
| *Motacilla tschutschensis* | 641 | KKBNA436-05 | DQ433817 |
| *Motacilla tschutschensis* | 679 | KKBNA607-05 | DQ433819 |
| *Nucifraga columbiana* | 680 | KKBNA602-05 | DQ433842 |
| *Nucifraga columbiana* | 674 | KKBNA547-05 | DQ433840 |
| *Nucifraga columbiana* | 673 | KKBNA252-05 | DQ433841 |
| *Nucifraga columbiana* | 652 | BOTW381-05 | DQ433044 |
| *Nucifraga columbiana* | 652 | BOTW380-05 | DQ433045 |
| *Nucifraga columbiana* | 670 | KKBNA723-05 | DQ433838 |
| *Nucifraga columbiana* | 670 | KKBNA606-05 | DQ433839 |
| *Numenius americanus* | 694 | TZBNA130-03 | AY666560 |
| *Numenius americanus* | 694 | TZBNA129-03 | AY666422 |
| *Numenius phaeopus* | 694 | KBNA314-04 | DQ434683 |
| *Oenanthe oenanthe* | 665 | TZBNA375-03 | AY666389 |
| *Onychoprion anaethetus* | 652 | BOTW412-05 | DQ433203 |
| *Onychoprion anaethetus* | 652 | BOTW411-05 | DQ433204 |
| *Onychoprion fuscatus* | 620 | CDUSM026-05 | DQ433208 |
| *Onychoprion fuscatus* | 652 | BOTW262-05 | DQ433210 |
| *Otus flammeolus* | 627 | HCBR066-03 | AY666497 |
| *Otus flammeolus* | 663 | HCBR065-03 | AY666515 |
| *Passer domesticus* | 694 | KBNA372-04 | DQ434706 |
| *Passer domesticus* | 694 | KBNA371-04 | DQ434705 |
| *Passer domesticus* | 689 | TZBNA014-03 | AY666316 |
| *Passer domesticus* | 670 | KKBNA109-04 | DQ433868 |
| *Passer montanus* | 694 | TZBNA162-03 | AY666248 |
| *Passer montanus* | 687 | TZBNA089-03 | AY666250 |
| *Perisoreus canadensis* | 685 | TZBNA057-03 | AY666354 |
| *Perisoreus canadensis* | 688 | TZBNA048-03 | AY666342 |
| *Phalacrocorax auritus* | 679 | TZBNA347-03 | AY666386 |
| *Phalacrocorax auritus* | 663 | TZBNA346-03 | AY666385 |
| *Phalacrocorax auritus* | 553 | CDLSU051-05 | DQ433077 |
| *Phalacrocorax pelagicus* | 669 | KKBNA753-05 | DQ433903 |
| *Phalacrocorax pelagicus* | 670 | KKBNA272-05 | DQ433905 |
| *Phalacrocorax pelagicus* | 670 | KKBNA271-05 | DQ433907 |
| *Phalacrocorax pelagicus* | 679 | KKBNA469-05 | DQ433909 |
| *Phalacrocorax pelagicus* | 670 | KKBNA015-04 | DQ433906 |
| *Phalacrocorax pelagicus* | 633 | KKBNA014-04 | DQ433902 |
| *Phalacrocorax pelagicus* | 678 | KKBNA013-04 | DQ433908 |
| *Phalacrocorax pelagicus* | 653 | KBNA938-04 | DQ433901 |
| *Phalacrocorax penicillatus* | 618 | CDAMH071-05 | DQ433079 |
| *Phalacrocorax penicillatus* | 672 | KKBNA300-05 | DQ433912 |
| *Phalacrocorax penicillatus* | 672 | KKBNA299-05 | DQ433913 |
| *Phalacrocorax penicillatus* | 671 | KKBNA298-05 | DQ433911 |
| *Phalacrocorax penicillatus* | 604 | KKBNA297-05 | DQ433910 |
| *Phalacrocorax urile* | 496 | CDAMH073-05 | DQ433080 |
| *Phalaropus fulicarius* | 694 | TZBNA134-03 | AY666308 |
| *Phalaropus fulicarius* | 694 | TZBNA125-03 | AY666310 |
| *Phalaropus lobatus* | 628 | KKBNA431-05 | DQ433919 |
| *Phalaropus lobatus* | 658 | KKBNA430-05 | DQ433921 |
| *Phalaropus lobatus* | 658 | KKBNA429-05 | DQ433922 |
| *Phalaropus lobatus* | 652 | BOTW273-05 | DQ433082 |
| *Phalaropus lobatus* | 669 | KKBNA210-05 | DQ433920 |
| *Phalaropus tricolor* | 695 | TZBNA158-03 | AY666195 |
| *Phalaropus tricolor* | 694 | TZBNA149-03 | AY666194 |
| *Phasianus colchicus* | 650 | HCBR056-03 | AY666562 |
| *Phasianus colchicus* | 658 | TZBNA052-03 | AY666332 |
| *Philomachus pugnax* | 564 | BOTW461-05 | DQ433086 |
| *Pica nuttalli* | 652 | BOTW399-05 | DQ433089 |
| *Pica nuttalli* | 652 | BOTW398-05 | DQ433090 |
| *Pica nuttalli* | 624 | CDAMH077-05 | DQ433088 |
| *Picoides albolarvatus* | 652 | BOTW276-05 | DQ433091 |
| *Picoides albolarvatus* | 670 | KKBNA306-05 | DQ433944 |
| *Picoides albolarvatus* | 679 | KKBNA305-05 | DQ433946 |
| *Picoides albolarvatus* | 658 | KKBNA212-05 | DQ433941 |
| *Picoides albolarvatus* | 679 | KKBNA516-05 | DQ433945 |
| *Picoides albolarvatus* | 670 | KKBNA464-05 | DQ433942 |
| *Picoides albolarvatus* | 670 | KKBNA453-05 | DQ433943 |
| *Picoides arcticus* | 753 | GTGC493-03 | AF394276 |
| *Picoides arcticus* | 651 | HCBR053-03 | AY666521 |
| *Picoides arizonae* | 670 | KKBNA744-05 | DQ433947 |
| *Picoides dorsalis* | 673 | KKBNA946-05 | DQ433950 |
| *Picoides dorsalis* | 674 | KKBNA945-05 | DQ433949 |
| *Picoides dorsalis* | 670 | KKBNA944-05 | DQ433948 |
| *Picoides dorsalis* | 692 | TZBNA029-03 | AY666363 |
| *Picoides nuttallii* | 676 | KKBNA565-05 | DQ433952 |
| *Picoides nuttallii* | 670 | KKBNA564-05 | DQ433951 |
| *Picoides nuttallii* | 607 | CDAMH079-05 | DQ433093 |
| *Picoides nuttallii* | 584 | CDAMH078-05 | DQ433092 |
| *Picoides pubescens* | 753 | GTGC512-03 | AF394295 |
| *Picoides pubescens* | 753 | GTGC511-03 | AF394294 |
| *Picoides pubescens* | 679 | KKBNA877-05 | DQ433953 |
| *Picoides pubescens* | 694 | TZBNA163-03 | AY666228 |
| *Picoides pubescens* | 694 | KBNA215-04 | DQ434712 |
| *Picoides scalaris* | 669 | KKBNA680-05 | DQ433954 |
| *Picoides scalaris* | 671 | KKBNA679-05 | DQ433955 |
| *Picoides villosus* | 753 | GTGC519-03 | AF394302 |
| *Picoides villosus* | 753 | GTGC518-03 | AF394301 |
| *Picoides villosus* | 694 | KBNA670-04 | DQ434714 |
| *Picoides villosus* | 694 | TZBNA267-03 | AY666557 |
| *Picoides villosus* | 670 | KKBNA439-05 | DQ433957 |
| *Picoides villosus* | 694 | KBNA287-04 | DQ434713 |
| *Picoides villosus* | 692 | TZBNA305-03 | AY666484 |
| *Picoides villosus* | 670 | KKBNA556-05 | DQ433956 |
| *Pinicola enucleator* | 694 | TZBNA198-03 | AY666321 |
| *Pinicola enucleator* | 694 | TZBNA197-03 | AY666314 |
| *Pluvialis dominica* | 694 | KBNA608-04 | DQ433962 |
| *Pluvialis dominica* | 694 | KBNA607-04 | DQ433963 |
| *Pluvialis dominica* | 694 | KBNA606-04 | DQ433964 |
| *Pluvialis dominica* | 694 | TZBNA136-03 | AY666317 |
| *Pluvialis fulva* | 652 | BOTW045-04 | DQ433123 |
| *Pluvialis squatarola* | 679 | TZBNA145-03 | AY666202 |
| *Pluvialis squatarola* | 652 | BOTW046-04 | DQ433124 |
| *Podiceps auritus* | 645 | BOTW095-04 | DQ433125 |
| *Podiceps grisegena* | 671 | KKBNA033-04 | DQ433966 |
| *Podiceps grisegena* | 670 | KKBNA032-04 | DQ433967 |
| *Podiceps grisegena* | 630 | KKBNA031-04 | DQ433965 |
| *Podiceps nigricollis* | 670 | KKBNA147-04 | DQ433968 |
| *Podiceps nigricollis* | 554 | CDLSU054-05 | DQ433126 |
| *Poecile atricapillus* | 694 | TZBNA024-03 | AY666351 |
| *Poecile atricapillus* | 694 | KBNA272-04 | DQ434723 |
| *Poecile atricapillus* | 648 | BOTW163-04 | DQ433128 |
| *Poecile carolinensis* | 652 | BOTW242-05 | DQ433130 |
| *Poecile carolinensis* | 646 | BOTW171-04 | DQ433129 |
| *Poecile gambeli PS-2* | 666 | KKBNA480-05 | DQ433978 |
| *Poecile gambeli PS-2* | 617 | KKBNA307-05 | DQ433976 |
| *Poecile gambeli PS-2* | 670 | KKBNA216-05 | DQ433979 |
| *Poecile gambeli PS-2* | 611 | KKBNA012-04 | DQ433977 |
| *Poecile hudsonica* | 524 | HCBR060-03 | AY666505 |
| *Poecile hudsonica* | 673 | KKBNA011-04 | DQ433980 |
| *Poecile rufescens* | 679 | KKBNA324-05 | DQ433986 |
| *Poecile rufescens* | 669 | KKBNA323-05 | DQ433981 |
| *Poecile rufescens* | 652 | BOTW275-05 | DQ433132 |
| *Poecile rufescens* | 670 | KKBNA217-05 | DQ433983 |
| *Poecile rufescens* | 670 | KKBNA578-05 | DQ433982 |
| *Poecile rufescens* | 680 | KKBNA509-05 | DQ433985 |
| *Poecile rufescens* | 676 | KKBNA508-05 | DQ433984 |
| *Poecile sclateri* | 624 | CDAMH065-05 | DQ433133 |
| *Porzana carolina* | 694 | TZBNA331-03 | AY666466 |
| *Porzana carolina* | 694 | KBNA129-04 | DQ433993 |
| *Porzana carolina* | 649 | BOTW079-04 | DQ433143 |
| *Rallus elegans* | 696 | TZBNA080-03 | AY666315 |
| *Rallus limicola* | 594 | BOTW177-04 | DQ433163 |
| *Rallus limicola* | 694 | CDANS099-05 | DQ434041 |
| *Regulus calendula* | 656 | HCBR043-03 | AY666517 |
| *Regulus calendula* | 668 | KKBNA923-05 | DQ434042 |
| *Regulus calendula* | 670 | KKBNA921-05 | DQ434043 |
| *Regulus calendula* | 643 | BOTW156-04 | DQ433166 |
| *Regulus satrapa* | 597 | KKBNA925-05 | DQ434045 |
| *Regulus satrapa* | 641 | KKBNA924-05 | DQ434044 |
| *Regulus satrapa* | 672 | KKBNA922-05 | DQ434047 |
| *Regulus satrapa* | 645 | KBNA022-04 | DQ434732 |
| *Regulus satrapa* | 694 | KBNA021-04 | DQ434731 |
| *Regulus satrapa* | 667 | KKBNA892-05 | DQ434046 |
| *Regulus satrapa* | 650 | BOTW210-04 | DQ433167 |
| *Scolopax minor* | 694 | KBNA472-04 | DQ434062 |
| *Scolopax minor* | 683 | KBNA471-04 | DQ434063 |
| *Scolopax minor* | 694 | KBNA470-04 | DQ434061 |
| *Scolopax minor* | 693 | TZBNA039-03 | AY666265 |
| *Scolopax minor* | 644 | BOTW054-04 | DQ433172 |
| *Sitta canadensis* | 672 | KKBNA920-05 | DQ434088 |
| *Sitta canadensis* | 548 | KKBNA919-05 | DQ434085 |
| *Sitta canadensis* | 538 | KKBNA917-05 | DQ434087 |
| *Sitta canadensis* | 539 | KKBNA916-05 | DQ434086 |
| *Sitta canadensis* | 679 | KKBNA915-05 | DQ434090 |
| *Sitta canadensis* | 670 | KKBNA914-05 | DQ434089 |
| *Sitta canadensis* | 657 | CDANS111-05 | DQ434084 |
| *Sitta carolinensis* | 662 | HCBR034-03 | AY666543 |
| *Sitta carolinensis* | 694 | KBNA200-04 | DQ434745 |
| *Sitta carolinensis* | 661 | KBNA199-04 | DQ434746 |
| *Sitta carolinensis* | 665 | KKBNA890-05 | DQ434092 |
| *Sitta carolinensis* | 612 | KKBNA878-05 | DQ434091 |
| *Sitta pusilla* | 670 | KKBNA224-05 | DQ434093 |
| *Sitta pusilla* | 652 | BOTW315-05 | DQ433182 |
| *Sitta pygmaea* | 667 | KKBNA427-05 | DQ434096 |
| *Sitta pygmaea* | 652 | BOTW233-05 | DQ433183 |
| *Sitta pygmaea* | 670 | KKBNA482-05 | DQ434097 |
| *Sitta pygmaea* | 667 | KKBNA481-05 | DQ434095 |
| *Sitta pygmaea* | 648 | KKBNA411-05 | DQ434094 |
| *Somateria fischeri* | 670 | KKBNA374-05 | DQ434099 |
| *Somateria fischeri* | 616 | KKBNA373-05 | DQ434098 |
| *Somateria fischeri* | 671 | KKBNA372-05 | DQ434102 |
| *Somateria fischeri* | 623 | CDAMH102-05 | DQ433184 |
| *Somateria fischeri* | 627 | CDAMH101-05 | DQ433185 |
| *Somateria mollissima* | 648 | TZBNA577-04 | AY666570 |
| *Somateria mollissima* | 656 | BRDC105-04 | DQ434751 |
| *Somateria mollissima* | 493 | TZBNA576-04 | AY666580 |
| *Somateria mollissima* | 664 | BRDC104-04 | DQ434748 |
| *Somateria mollissima* | 688 | BRDC103-04 | DQ434747 |
| *Somateria mollissima* | 660 | BRDC102-04 | DQ434750 |
| *Somateria mollissima* | 661 | BRDC101-04 | DQ434749 |
| *Somateria mollissima* | 693 | TZBNA019-03 | AY666247 |
| *Somateria mollissima* | 587 | CDLSU058-05 | DQ433186 |
| *Somateria mollissima* | 688 | TZBNA349-03 | AY666387 |
| *Somateria spectabilis* | 652 | BOTW271-05 | DQ433187 |
| *Somateria spectabilis* | 694 | KBNA559-04 | DQ434106 |
| *Stercorarius longicaudus* | 660 | KKBNA435-05 | DQ434144 |
| *Stercorarius longicaudus* | 659 | KKBNA432-05 | DQ434145 |
| *Stercorarius pomarinus* | 621 | CDUSM024-05 | DQ433198 |
| *Stercorarius pomarinus* | 616 | CDUSM023-05 | DQ433200 |
| *Stercorarius pomarinus* | 617 | CDUSM022-05 | DQ433199 |
| *Stercorarius pomarinus* | 670 | KBNA749-04 | DQ434148 |
| *Sterna forsteri* | 694 | KBNA630-04 | DQ434162 |
| *Sterna forsteri* | 650 | BOTW169-04 | DQ433207 |
| *Sterna hirundo* | 678 | KKBNA910-05 | DQ434164 |
| *Sterna hirundo* | 670 | KKBNA909-05 | DQ434163 |
| *Sterna paradisaea* | 648 | TZBNA143-03 | AY666237 |
| *Sternula antillarum* | 652 | BOTW292-05 | DQ433206 |
| *Sternula antillarum* | 645 | BOTW187-04 | DQ433205 |
| *Streptopelia decaocto* | 617 | BOTW082-04 | DQ433219 |
| *Streptopelia decaocto* | 652 | BOTW312-05 | DQ433220 |
| *Strix nebulosa* | 667 | TZBNA361-03 | AY666432 |
| *Strix nebulosa* | 679 | KKBNA058-04 | DQ434173 |
| *Strix nebulosa* | 652 | BOTW416-05 | DQ433221 |
| *Strix nebulosa* | 652 | BOTW415-05 | DQ433222 |
| *Strix occidentalis* | 652 | BOTW291-05 | DQ433223 |
| *Strix occidentalis* | 680 | KKBNA964-05 | DQ434177 |
| *Strix occidentalis* | 652 | BOTW252-05 | DQ433224 |
| *Strix occidentalis* | 668 | KKBNA963-05 | DQ434174 |
| *Strix occidentalis* | 680 | KKBNA962-05 | DQ434178 |
| *Strix occidentalis* | 670 | KKBNA961-05 | DQ434175 |
| *Strix occidentalis* | 671 | KKBNA284-05 | DQ434176 |
| *Strix varia* | 669 | KKBNA050-04 | DQ434179 |
| *Strix varia* | 642 | BOTW074-04 | DQ433225 |
| *Strix varia* | 615 | TZBNA371-03 | AY666428 |
| *Strix varia* | 694 | KBNA479-04 | DQ434180 |
| *Sturnus vulgaris* | 694 | KBNA358-04 | DQ434774 |
| *Sturnus vulgaris* | 687 | TZBNA053-03 | AY666174 |
| *Sturnus vulgaris* | 679 | KKBNA125-04 | DQ434182 |
| *Sturnus vulgaris* | 670 | KKBNA124-04 | DQ434181 |
| *Sturnus vulgaris* | 694 | KBNA447-04 | DQ434772 |
| *Sturnus vulgaris* | 615 | BOTW459-05 | DQ433228 |
| *Sturnus vulgaris* | 694 | KBNA153-04 | DQ434773 |
| *Synthliboramphus antiquus* | 694 | TZBNA236-03 | AY666374 |
| *Synthliboramphus antiquus* | 694 | KBNA584-04 | DQ434183 |
| *Synthliboramphus hypoleucus* | 670 | KKBNA330-05 | DQ434184 |
| *Thalasseus elegans* | 673 | KKBNA632-05 | DQ434159 |
| *Thalasseus elegans* | 672 | KKBNA631-05 | DQ434161 |
| *Thalasseus elegans* | 673 | KKBNA630-05 | DQ434160 |
| *Thalasseus elegans* | 673 | KKBNA629-05 | DQ434158 |
| *Thalasseus elegans* | 673 | KKBNA628-05 | DQ434157 |
| *Thalasseus maximus* | 621 | CDUSM029-05 | DQ433211 |
| *Thalasseus maximus* | 618 | CDUSM028-05 | DQ433213 |
| *Thalasseus maximus* | 667 | KKBNA470-05 | DQ434165 |
| *Thalasseus maximus* | 619 | CDUSM095-05 | DQ433212 |
| *Thalasseus sandvicensis* | 679 | KKBNA229-05 | DQ434171 |
| *Thalasseus sandvicensis* | 608 | CDUSM031-05 | DQ433217 |
| *Thalasseus sandvicensis* | 670 | KKBNA472-05 | DQ434170 |
| *Thalasseus sandvicensis* | 632 | KKBNA768-05 | DQ434169 |
| *Thalasseus sandvicensis* | 652 | BOTW298-05 | DQ433218 |
| *Thalasseus sandvicensis* | 609 | CDUSM096-05 | DQ433216 |
| *Tringa flavipes* | 664 | TZBNA121-03 | AY666309 |
| *Tringa flavipes* | 621 | BOTW058-04 | DQ433241 |
| *Tringa melanoleuca* | 694 | TZBNA139-03 | AY666263 |
| *Tringa melanoleuca* | 651 | BOTW057-04 | DQ433242 |
| *Tringa semipalmata* | 644 | BOTW056-04 | DQ432831 |
| *Tringa solitaria PS-1* | 694 | TZBNA148-03 | AY666198 |
| *Tringa solitaria PS-1* | 655 | TZBNA557-03 | AY666532 |
| *Tringa solitaria PS-1* | 666 | TZBNA556-03 | AY666526 |
| *Tringa solitaria PS-1* | 660 | TZBNA555-03 | AY666524 |
| *Tringa solitaria PS-1* | 652 | BOTW012-04 | DQ433243 |
| *Troglodytes aedon* | 694 | KBNA198-04 | DQ434785 |
| *Troglodytes aedon* | 694 | KBNA060-04 | DQ434787 |
| *Troglodytes aedon* | 694 | KBNA059-04 | DQ434786 |
| *Troglodytes troglodytes PS-1* | 657 | TZBNA319-03 | AY666410 |
| *Troglodytes troglodytes PS-1* | 694 | KBNA101-04 | DQ434789 |
| *Troglodytes troglodytes PS-1* | 666 | TZBNA320-03 | AY666470 |
| *Troglodytes troglodytes PS-1* | 647 | KBNA432-04 | DQ434790 |
| *Troglodytes troglodytes PS-1* | 694 | KBNA102-04 | DQ434788 |
| *Troglodytes troglodytes PS-2* | 672 | KKBNA473-05 | DQ434198 |
| *Troglodytes troglodytes PS-2* | 673 | KKBNA765-05 | DQ434197 |
| *Turdus migratorius* | 692 | TZBNA187-03 | AY666254 |
| *Turdus migratorius* | 695 | TZBNA107-03 | AY666256 |
| *Turdus migratorius* | 647 | BOTW184-04 | DQ433244 |
| *Turdus migratorius* | 614 | KBNA026-04 | DQ434793 |
| *Turdus migratorius* | 646 | KBNA025-04 | DQ434792 |
| *Turdus migratorius* | 694 | KBNA221-04 | DQ434791 |
| *Turdus migratorius* | 669 | BOTW010-04 | DQ433245 |
| *Uria aalge* | 694 | KBNA517-04 | DQ434794 |
| *Uria aalge* | 652 | BOTW294-05 | DQ433250 |
| *Uria lomvia* | 694 | KBNA604-04 | DQ434214 |
| *Uria lomvia* | 694 | KBNA603-04 | DQ434215 |
| *Uria lomvia* | 694 | TZBNA237-03 | AY666277 |
